# New triterpenes from *Cimicifuga yunnanensis* down-regulating the mRNA expression of CD147, MMP-2, and MMP-9†

**DOI:** 10.1039/d1ra07828c

**Published:** 2021-11-17

**Authors:** Ni-Hong Lu, Jie Li, Yong-Rui Yang, Hong-Lu Liu, Ying-Rong Du

**Affiliations:** Department of Respiratory Medicine, The Third People's Hospital of Kunming Yunnan 650041 People's Republic of China 602157606@qq.com

## Abstract

Eleven new 9,19-cycloartane triterpenes (1–9, 11–12) and one undescribed lanostane-type aglycone (10) were identified from the aerial parts of *Cimicifuga yunnanensis*. The new structures were elucidated by analysis of spectroscopic data. Compounds 3–5, 7–9, and 11, without obvious cytotoxicity at 50 μM, were evaluated for inhibiting the mRNA expressions of atherosclerosis-related factors of CD147 (extracellular matrix metalloproteinase inducer, EMMPRIN), matrix metalloproteinase 2 (MMP-2) and MMP-9 in phorbol-12-myristate-13-acetate (PMA) induced Human monocytic THP-1 cells by using a quantitative real-time PCR method (q-PCR). Among them, aglycones 7 and 8 showed potent activities, whereas all tested glycosides were inactive. Compounds 7 and 8 suppressed the mRNA expression of CD147 in a dose-dependent manner, with an IC_50_ value of 3.38 ± 0.27 μM and 8.25 ± 0.33 μM, respectively. Besides, 7 dose-related down-regulated the mRNA expression of MMP-2, and MMP-9, having an IC_50_ value of 6.32 ± 0.31 μM and 11.57 ± 0.23 μM, respectively. Meanwhile, 8 at 10 μM reduced the mRNA expression of MMP-2 and MMP-9 by 35% and 25%, respectively. Significantly, the migration ability of the induced THP-1 cells was potently and dose-dependently inhibited by 7, with an IC_50_ value of 5.87 ± 0.27 μM.

## Abstract 2

Eleven new 9,19-cycloartane triterpenes (CTs) (1–9, 11–12) and one undescribed lanostane-type aglycone (10) were identified from *Cimicifuga yunnanensis*. CTs aglycones 7 and 8 potently down-regulated the mRNA expression of CD147, MMP-2, and MMP-9.

## Graphical abstract

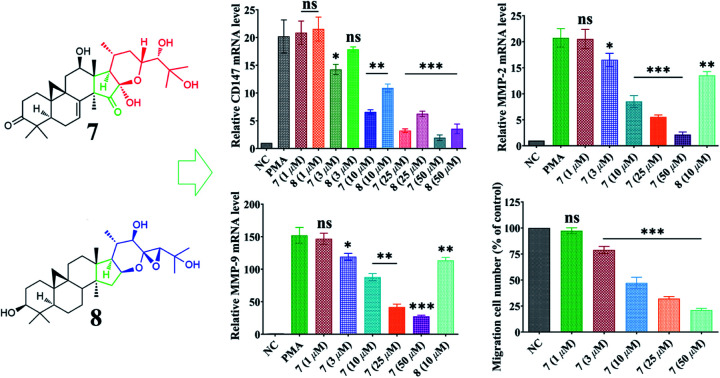

## 1. Introduction

Cardiovascular diseases (CVDs) are leading causes of death globally, by which more than 17.0 million people die every year.^1,2^ Pathologically, atherosclerosis, a chronic inflammatory disease, is a critical causing factor of CVDs.^1,3^ Patients who can maintain stability of atherosclerotic plaques will adapt to stable angina pectoris. Otherwise, a life-threatening acute coronary syndrome, including acute myocardial infarction (AMI) and unstable angina pectoris (UA) would happen to them.^4–6^ CD147, extracellular matrix metalloproteinase inducer, and metalloproteinases (MMPs), such as MMP-2 and MMP-9 (a novel marker of AMI), are overexpressed in advanced atherosclerotic plaques by monocytes/macrophages and have been shown to contribute to AMI and UA through degradation of the extracellular matrix.^7–10^ Of note, CD147 stimulates macrophages to produce MMP-2 and MMP-9 in a paracrine or autocrine way.^4,11^ Therefore, to suppress the expression of CD147 and MMPs represents a promising strategy for anti-atherosclerosis.

**Fig. 1:**
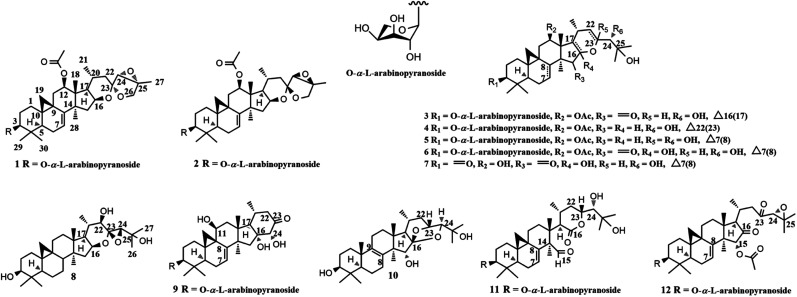
Structures of compounds 1–12.

Plants of *Cimicifuga* genus (*C. racemosa*, *C. foetida*, and *C. simplex*) are famous herb medicines in Europe, the United States, and East Asia.^12,13^ These herbs mainly contain 9,19-cycloartane triterpenes (CTs) with diverse bioactivities, such as cytotoxicity,^14,15^ anti-angiogenic,^16^ anti-inflammatory,^17,18^ and neuro-protective.^19,20^ Recently, we identified two CTs, yunnanterpene G (YG) and 12β-hydroxycimiacerol (HC), with anti-atherosclerosis potentials by potently suppressing the mRNA expressions of CD147 and MMPs.^21,22^ As a part of our successive program to explore bioactive CTs from *Cimicifuga* spp, nine unreported CTs glycosides (1–6, 9, and 11–12) and two new aglycones (7 and 8), together with one undescribed lanostane-type triterpene (10) were identified from the aerial parts of *C. yunnanensis* (Fig. 1), an indigenous species distributed in the southwest region of China.^16^ Significantly, the q-PCR experiments showed that aglycones 7 and 8 dose-dependently attenuated the mRNA expression of CD147, with an IC_50_ value of 3.38 ± 0.27 μM and 8.25 ± 0.33 μM, respectively, in PMA-induced THP-1 cells. Of note, the CD147 mRNA inhibitory effect of 7 at 10 μM is more potent than that of YG (positive control). While, 8 has comparable activity as YG at this concentration. Moreover, 7 dose-dependently down-regulated the mRNA expression of MMP-2, and MMP-9 and suppressed the migration ability of the induced THP-1 cells, having an IC_50_ value of 6.32 ± 0.31 μM, 11.57 ± 0.23 μM, and 5.87 ± 0.27 μM, respectively. Conversely, all tested glycosides (3–5, 9 and 11) were inactive at 10 μM. Described herein are the isolation, structure elucidation, and biological activities of compounds 1–12.

## 2. Results and discussion

### 2.1. Structural elucidation of compounds 1–12

The same molecular formula C_37_H_54_O_10_ for compounds 1 and 2 were determined by the HRESIMS ([M]^+^*m*/*z* 658.3703, calcd 658.3717 for 1, and [M]^+^*m*/*z* 658.3694, calcd 658.3717 for 2, respectively). The IR spectra showed absorptions for OH (3443 cm^−1^ for 1 and 3441 for 2), and carbonyl (1733 cm^−1^ both for 1 and 2) groups. In the ^1^H NMR (Table 1) spectrum of 1, downfield shifted cyclopropane methylene signals at *δ*_H_ 0.57 (1H, d, *J* = 3.9 Hz) and 1.07 (1H, overlapped), a secondary methyl group at *δ*_H_ 0.95 (d, *J* = 6.0 Hz), six tertiary methyl groups at *δ*_H_ 0.95–1.47, and an anomeric proton at *δ*_H_ 4.79 (1H, d, *J* = 7.2 Hz) were observed. The ^13^C spectrum of 1 (Table 2) showed the existence of an ester carbonyl group at *δ*_C_ 171.1 (s), two olefinic carbons at *δ*_C_ 114.5 (C-7, d) and 148.2 (C-7, s), and six oxygenated carbon atoms at *δ*_C_ 88.3 (C-3, d), 77.1 (C-12, d), 73.5 (C-16, d), 63.6 (C-24, d), 63.7 (C-25, s), and 69.2 (C-26, t), respectively. These data suggested that 1 was a highly oxygen-bearing CTs glycoside with a seven-ring skeleton.

**Table 1:** ^1^H NMR Spectroscopic Data of Compounds 1-12 (*δ* in ppm, *J* in Hz) in Pyridine-*d*_5_

Position	1^a^	2^a^	3^b^	4^b^	5^b^	6^b^	7^b^	8^a^	9^b^	10^a^	11^b^	12^a^
1	1.57 m	1.54 m	1.53 m	1.50 m	1.55 m	1.46 m	1.81 m	1.55m	2.77 m	1.72 m	1.61 m	1.64 m
	1.16 m	1.11 m	1.08 m	1.10 m	1.12 m	1.12^c^	1.53^c^	1.21 m	1.71 m	1.26 m	1.21^c^	1.28^c^
2	2.30 m	2.24 m	2.37 m	2.33 m	2.27 m	2.27 m	2.70 ddd (27.8, 13.9, 6.2)	2.00 m	2.46 m	1.92 m (2H)	2.37 m	2.35 m
	1.90 m	1.85 m	1.90 m	1.89 m	1.81^a^	1.89 m	2.24 m	1.90 m	2.11 m		1.95 m	2.23^c^
3	3.45 dd (11.7, 4.2)	3.39 m	3.47 dd (11.5,4.1)	3.48 dd (11.7, 4.2)	3.43 dd (11.7, 4.2)	3.45 dd (11.5, 3.9)		3.55 m	3.59 dd (11.4, 3.9)	3.48 m	3.46 dd (11.5, 3.9)	3.48 m
4												
5	1.19 m	1.11 m	1.27^c^	1.24 m	1.18 m	1.19 m	1.53^c^	1.30 dd (12.6, 4.1)	1.36^c^	1.21^c^	1.25^a^	1.24 m
6	1.82 m	1.73 m	2.50 m	1.46 m	1.81^c^	1.97 m	1.83 m	1.56 m	1.92 m	1.76 m	1.89 m	1.77 m
	1.54 m	1.44^c^	1.05 m	0.71 m	1.54 m	1.61^c^	1.75 m	0.75 m	1.76 m	1.55 m	1.00^c^	1.44 m
7	5.15^c^	5.08^c^	1.57 m	1.25 m	5.13 d (7.2)	6.73 brd (6.3)	6.75 d (7.1)	1.57 m	5.21 brd (6.3)	2.66 m	5.25 brd (5.8)	5.31 d (6.1)
			0.73 dd (25.0,12.5)	0.94 m				1.03 m		2.46 m		
8			2.00 dd (12.5,4.0)					1.55 m				
9												
10												
11	2.96 dd (16.1, 9.1)	2.91 dd (15.7, 9.3)	2.97 dd (16.2, 9.4)	2.66 dd (15.9, 9.4)	2.93 dd (16.0, 9.3)	2.91 dd (16.2, 8.9)	2.82 dd (15.3, 8.6)	1.98 m	4.60 brd (6.6)	2.08 m	2.06 m	2.09 m
	1.27 m	1.23^c^	1.16 d (15.3)	1.16 m	1.25 m	1.36 m	1.57^c^	1.10^c^		2.02 m	1.55 m	1.14 m
12	5.23 d (8.5)	5.20 d (8.4)	5.74 d (7.8)	5.34 dd (9.3, 4.0)	5.23 d (8.9)	5.49 d (8.5)	4.53 d (8.5)	1.59 m (2H)	2.85 dd (13.6, 9.5)	1.73 m	1.92 m	1.85 m (2H)
									2.08 m	1.26 m	1.76 m	
13												
14												
15	2.16 m	2.09 m (2H)		1.97 m	2.17^c^			1.94 m	2.56 m		9.82 s	5.88 s
	2.03 m			1.88 m	2.05 dd (13.6, 5.9)			1.66 m	2.27 d (7.2)			
16	4.63 dd (14.1, 7.4)	4.31^c^		4.23 m	4.89 m			5.01 dd (16.2, 7.9)		4.54 d (8.7)		
17	1.79 m	1.76 m		2.10 m	1.81^c^	2.38 d (7.0)	2.47 d (7.4)	1.61 m	2.25 d (4.8)	1.50^c^	2.72 d (4.4)	2.35 m
18	1.41 s	1.46 s	1.67	1.35 s	1.42 s	1.00 s	1.64 s	1.24 s	1.26 s	0.92 s	1.54 s	1.22 s
19	1.07^c^	0.99^c^	0.58 d (4.0)	0.59 d (3.7)	1.02^c^	1.07^c^	1.32^c^	0.51 d (3.9)	2.01 d (3.4)	1.07 s	0.89 d (3.8)	0.97^c^
	0.57 d (3.9)	0.47 brs	0.26 d (4.0)	0.20 d (4.1)	0.52 d (3.8)	0.57 d (4.0)	0.92 d (4.3)	0.24 d (4.1)	1.02 d (3.4)		0.49 d (3.8)	0.50 d (3.9)
20	1.82 m	2.22 m	3.37 dd (13.8,7.0)	2.48 m	1.95 m	2.02 m	1.58^c^	2.31 m	2.17 m	1.68 m	2.29 m	2.59 m
21	0.95 d (6.0)	0.98 d (6.2)	1.61 d (6.9)	1.15 d (7.2)	0.99 brd (4.0)	1.11 d (6.2)	1.53 d (6.2)	1.26 d (6.4)	0.90 d (5.9)	0.91 d (6.9)	1.00^c^	1.06 d (6.6)
22	2.19 m	1.55 m	2.71 2H m	5.22 d (3.2)	2.85 brd (11.7)	2.53 d (13.0)	2.61 d (12.8)	3.94 d (10.7)	2.51 m	2.29 m	2.09 dd (13.5, 6.9)	3.57 dd (15.0, 3.2)
	1.65 m	1.42 m			1.58 m	1.68 d (15.5)	1.72 m		2.43 m	1.05 m	1.89 m	2.74 dd (18.0, 8.2)
23			4.64 m			4.64 brd (10.4)	4.71 brd (9.9)			4.80 d (9.0)	5.13 brd (11.5)	
24	3.73 s	3.66 s	3.64 s	4.35 brs	3.78 brs	3.59 brs	3.59 brs	4.22 s	4.51 s	3.82 s	3.74 d (4.6)	3.72 s
25												
26	3.97 d (10.0)	4.03 d (10.0)	1.60 s	1.63 s	1.84 s	1.58 s	1.59^c^	1.81 s		1.50 s	1.66 s	1.33 s
	3.91 d (10.0)	3.60 d (10.0)										
27	1.47 s	1.46 s	1.67 s	1.68 s	1.78 s	1.59 s	1.61^c^	1.72 s		1.52 s	1.71 s	1.33 s
28	1.07 s	1.02 s	1.31 s	0.88 s	1.05 s	1.54 s	1.60^c^	0.87 s	1.60 s	1.29 s	1.59 s	1.29 s
29	0.99 s	0.93 s	1.27 s	1.01 s	0.99^c^	1.29 s	1.11 s	1.09 s	1.38 s	1.08 s	1.25 s	1.02 s
30	1.32 s	1.26 s	0.97 s	1.32 s	1.29 s	1.50 s	1.02 s	1.24 s	1.12 s	1.21 s	0.99 s	1.30 s
1′	4.79 d (7.2)	4.74 d (7.1)	4.79 d (7.0)	4.81 d (7.1)	4.76 d (7.2)	4.76 d (7.1)			4.82 d (7.0)		4.77 d (7.0)	4.78 d (7.2)
2′	4.49 m	4.44 t (7.8)	4.47 m	4.49 t (8.8)	4.45 t (8.0)	4.45 t (8.0)			4.47 m		4.45 t (5.9)	4.47 m
3′	4.19 m	4.14 brd (7.3)	4.16 dd (8.8, 3.0)	4.19 dd (9.0, 3.4)	4.14 dd (8.8, 3.2)	4.15 dd (8.8,3.1)			4.17 dd (8.7,3.0)		4.17 brd (8.7)	4.16 dd (8.9, 3.2)
4′	4.34 brs	4.30 brs	4.31 m	4.34 brs	4.30 brs	4.31 m			4.32 m		4.33 m	4.32 brs
5′	4.32 m	4.29^c^	4.28 m	4.32 m	4.29 brd (10.9)	4.28 m			4.28 m		4.30 m	4.30 m
	3.80 m	3.76 d (11.2)	3.79 d (10.6)	3.82 m	3.77 brd (9.6)	3.79 m			3.79 m		3.80 brd (10.8)	3.79 m
12-OCOCH3	2.22 s	2.17 s	2.28 s	2.14 s	2.16 s	2.24 s						
15-OCOCH3	3.36 s											2.23 s

a Recorded at 500 MHz in pyridine-*d*_5_.

b Recorded at 600 MHz in pyridine-*d*_5_.

c Signals overlapped.

**Table 2:** ^13^C NMR Spectroscopic Data (*δ* in ppm) of Compounds 1-12 in Pyridine-*d*_5_^a^

Position	1	2	3	4	5	6	7	8	9	10	11	12
1	30.6 t	30.2 t	32.1 t	32.4 t	30.3 t	30.1 t	31.8 t	32.8 t	27.3 t	36.5 t	31.0 t	30.2 t
2	29.9 t	29.5 t	29.8 t	30.3 t	29.4 t	29.3 t	36.7 t	31.7 t	29.7 t	29.2 t	29.3 t	29.4 t
3	88.3 d	87.9 d	87.9 d	87.9 d	87.9 d	87.7 d	214.6 s	78.3 d	88.3 d	78.5 d	87.7 d	88.1 d
4	40.8 s	40.4 s	41.1 s	41.7 s	40.4 s	40.2 s	48.7 s	41.5 s	40.6 s	39.9 s	40.2 s	40.4 s
5	42.8 d	42.4 d	46.8 d	47.5 d	42.5 d	41.4 d	43.1 d	47.8 d	44.0 d	51.3 d	40.4 d	42.5 d
6	22.2 t	21.8 t	25.6 t	20.8 t	21.9 t	21.5 t	21.7 t	21.7 t	21.9 t	19.1 t	22.2 t	21.9 t
7	114.5 d	114.1 d	20.4 t	26.1 t	114.0 d	117.2 d	116.7 d	27.2 t	113.6 d	28.5 t	122.0 d	115.2 d
8	148.2 s	147.7 s	40.6 d	47.1 d	147.9 s	140.3 s	141.3 s	48.1 d	147.4 s	134.9 s	140.2 s	146.1 s
9	21.7 s	21.2 s	19.9 s	20.9 s	21.3 s	21.2 s	29.8 s	20.1 s	27.3 s	136.0 s	18.8 s	21.4 s
10	28.7 s	28.2 s	26.8 s	27.6 s	28.3 s	28.3 s	22.6 s	26.7 s	29.0 s	38.0 s	28.4 s	28.7 s
11	36.9 t	36.6 t	36.8 t	36.9 t	36.7 t	35.6 t	39.2 t	26.9 t	63.3 d	21.2 t	24.6 t	24.9 t
12	77.1 d	76.8 d	71.0 d	77.4 d	76.9 d	76.1 d	71.3 d	33.9 t	48.8 t	32.8 t	30.8 t	33.3 t
13	48.5 s	48.1 s	49.4 s	49.4 s	48.1 s	43.6 s	45.1 s	45.7 s	46.2 s	41.6 s	42.9 s	41.4 s
14	51.1 s	50.5 s	54.5 s	48.3 s	50.7 s	54.6 s	57.8 s	47.3 s	50.7 s	49.5 s	59.3 s	48.5 s
15	42.9 t	43.0 t	207.3 s	46.2 t	42.7 t	211.6 s	212.2 s	43.8 t	48.4 t	76.3 d	200.3 d	81.6 d
16	73.5 d	74.5 d	148.6 s	75.2 d	71.8 d	95.9 s	96.1 s	72.8 d	81.9 s	112.7 s	173.7 s	214.0 s
17	57.2 d	56.6 d	151.0 s	52.8 d	57.4 d	55.6 d	56.9 d	52.8 d	63.5 d	58.7 d	55.3 d	58.9 d
18	14.3 q	14.2 q	25.7 q	13.6 q	14.9 q	13.9 q	15.1 q	21.2 q	21.0 q	18.2 q	21.9 q	21.7 q
19	29.1 t	28.8 t	31.3 t	30.3 t	28.8 t	28.0 t	27.6 t	30.9 t	18.5 t	19.6 q	28.5 t	27.9 t
20	26.2 d	23.1 d	28.0 d	25.1 d	25.9 d	25.4 d	26.1 d	35.2 d	25.7 d	24.9 d	27.9 d	27.9 d
21	22.1 q	21.4 q	17.3 q	26.1 q	21.2 q	23.4 q	23.7 q	17.9 q	20.5 q	20.5 d	24.7 q	20.8 q
22	37.1 t	37.2 t	40.7 t	106.2 d	42.1 t	32.4 t	32.5 t	87.4 d	44.7 t	38.6 t	36.2 t	46.5 t
23	106.4 s	105.9 s	68.3 d	154.2 s	102.5 s	75.7 d	76.1 d	106.5 s	211.2 s	72.5 d	78.0 d	205.5 s
24	63.6 d	62.3 d	78.7 d	79.9 d	77.5 d	78.7 d	78.5 d	83.7 d	82.3 d	90.7 d	79.7 d	65.8 d
25	63.7 s	62.5 s	73.6 s	73.5 s	75.1 s	72.6 s	72.8 s	84.0 s		71.4 s	72.2 s	60.8 s
26	69.2 t	68.1 t	27.7 q	27.9 q	29.4 q	26.1 q	28.5 q	28.2 q		27.6 q	25.8 q	24.6 q
27	15.2 q	14.8 q	27.1 q	26.4 q	28.9 q	28.5 q	26.2 q	25.4 q		25.8 q	29.0 q	18.4 q
28	27.2 q	26.9 q	23.6 q	21.3 q	26.8 q	24.2 q	24.2 q	20.2 q	28.0 q	18.3 q	18.6 q	19.5 q
29	14.6 q	14.3 q	25.5 q	15.7 q	14.2 q	25.4 q	22.3 q	14.9 q	25.8 q	16.9 q	25.3 q	14.2 q
30	26.2 q	25.8 q	15.2 q	26.3 q	25.7 q	15.7 q	19.8 q	26.2 q	14.4 q	29.0 q	13.7 q	25.8 q
1′	108.0 d	107.3 d	107.4 d	108.1 d	107.3 d	107.4 d			107.4 d		107.3 d	107.4 d
2′	73.5 d	72.9 d	72.9 d	73.4 d	72.9 d	72.8 d			72.8 d		72.8 d	72.9 d
3′	75.2 d	74.6 d	74.6 d	75.1 d	74.6 d	74.5 d			74.5 d		74.5 d	74.7 d
4′	70.2 d	69.4 d	69.5 d	70.1 d	69.5 d	69.5 d			69.5 d		69.4 d	69.5 d
5′	67.5 t	66.6 t	66.8 t	67.4 t	66.7 t	66.8 d			66.8 t		66.7 t	66.8 t
12-OC̲OCH_3_	171.2 s	170.8 s	170.8 s	171.2 s	170.6 s	170.7 s						
12-OCOC̲H_3_	21.4 q	21.6 q	21.4 q	21.7 q	21.6 q	21.2 q						
15-OC̲OCH_3_												170.2 s
15-OCOC̲H_3_												20.9 q

a ^13^C Recorded at 150 MHz in pyridine-*d*_5_.

**Fig. 2:**
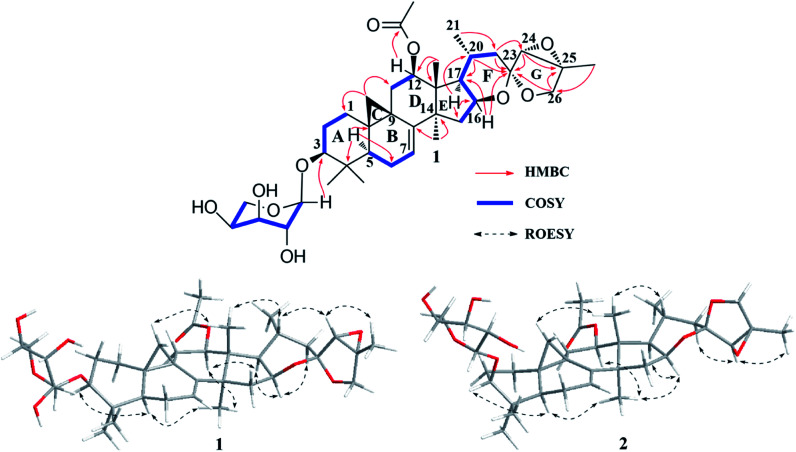
Major HMBC, ^1^H–^1^H COSY of compound 1, and key ROESY correlations of 1 and 2.

The ^1^H–^1^H COSY (Fig. 2) spin system of –CH_2_CHCHCH(CH_3_)CH_2_– (for C-15 to C-17, C-20 to C-22), together with the diagnostic ketal signal at *δ*_C_ 106.4 (C-23, s), as well as the pair of geminal signals for CH_2_-26 at *δ*_H_ 3.91 and 3.97 (each 1H, d, *J* = 10.0 Hz), indicated that 1 was a acteol-type CTs.^12,23^ HMBC couplings from H-16 (*δ*_H_ 4.63), H-22 (*δ*_H_ 1.65 and 2.19) to C-23 (*δ*_C_ 106.4), H-22 (*δ*_H_ 1.65 and 2.19) to C-24 (*δ*_C_ 63.6), and H-26 (*δ*_H_ 3.91 and 3.97) and H-24 (*δ*_H_ 3.73) to C-23 (*δ*_C_ 109.5) and C-25 (*δ*_C_ 83.4), further supported this deduction. The location of sugar unit at C-3 was inferred from HMBC correlation between the anomeric proton at *δ*_H_ 4.85 (1H, d, *J* = 8.6 Hz) and the methine signal at *δ*_C_ 88.5 (C-3). In addition, the sugar was determined as l-arabinose by comparing its TLC and specific rotation with a standard after acid hydrolysis. Structurally, 1 resembles to 26-deoxyactein,^23^ with main differences as that an acetoxy group substituted at C-12 and the presence of a double bond at C-7 and C-8. These elucidations were confirmed by HMBC association from H-12 (*δ*_H_ 5.23) to the ester carbonyl group (*δ*_C_ 171.1), and the ^1^H–^1^H COSY correlation of the olefinic proton resonance (*δ*_H_ 5.15) and H-6 (*δ*_H_ 1.54 and 1.82).

In the ROESY spectrum (Fig. 2), cross-peaks of H-3 with H-5 (biogenetically α-oriented), H-16 and H-17 with CH_3_-28 (biogenetically α-oriented), and H-20 with CH_3_-18 (biogenetically β-oriented) were observed, which helped to establish the relative configuration of the core structure of 1. Moreover, the characteristic ROESY correlations of H-21/H-24 and H-24/CH_3_-27 further decided the configuration of ring F and G, as well as the ternary epoxy ring and ring G as shown (Fig. 2). Intensive analysis of 1 D and 2D NMR spectra demonstrated that compound 2 had the same core structure as that of 1, with the major differences being at C-24, C-25 and C26. Diagnostically, the ROESY correlation of H-21/H-24 was absence in 2, instead, the association of H-22α/H-24 was observed. Thus, the configuration of ring F and G of 2 was determined as same to 23-*epi*-26-deoxyactein (Fig. 2).^23^ Finally, the structure of 1 and 2 were determined as 7(8)-en-acteol-3-*O*-α-l-arabinopyranoside and 7(8)-en-23-*epi*-acteol-3-*O*-α-l-arabinopyranoside, respectively.

Compound 3, white powder, had molecular formula C_37_H_58_O_12_ based on the HRTOF-ESIMS at *m*/*z* 717.3823 [M + Na + H_2_O]^+^ (calcd 717.3826). The ^1^H NMR spectrum (Table 1) showed characteristic cyclopropane methylene signals at *δ*_H_ 0.26 (1H, d, *J* = 4.0 Hz) and 0.58 (1H, d, *J* = 4.0 Hz), and an anomeric proton at *δ*_H_ 4.79 (1H, d, *J* = 7.2 Hz). The ^13^C NMR spectrum (Table 2) indicated that 3 had resonances corresponding to an ester carbonyl group at *δ*_C_ 171.1 (s), and an α,β-unsaturated ketone unit at *δ*_C_ 207.3 (C-15, s), 148.6 (C-16, s) and 151.0 (C-17, s). Aforementioned data indicated that 3 was a CTs glycoside. The sugar unite was determined as l-arabinose by the same way as that of 1. The NMR data of aglycone part of 3 resembled that of 24-*O*-acetyl-16(17)-en-hydroshengmanol-3-*O*-β-d-xylopyranoside,^20^ except that the acetoxy group was changed to C-12 and the OH-16 was replaced by a carbonyl group. HMBC correlations of H-12 (*δ*_H_ 5.74) with the ester carbonyl group (*δ*_C_ 170.8), and CH_3_-28 (*δ*_H_ 1.31) with the carbonyl carbon (*δ*_C_ 207.3) further confirmed these elucidations. ROESY cross-peaks of H-3/H5, H-12/CH_3_-28, and H-20/CH_3_-18 in 3 suggested the α-orientation of H-3, H-12, and CH_3_-21. The β-orientation of H-24 was deduced by the ROESY correlation of H-24/H-20. In addition, identical to isodahurinyl-type molecules, H-24 of 3 was a singlet in ^1^H NMR spectrum, suggesting *S* configuration of C-24 (the coupling constant of H-24 and H-23 of dahurinyl-type compounds, with *R* configuration of C-24, is around 6–9 Hz).^15^ Therefore, the structure of 3 was determined as 12β-acetoxy-16(17)-en-isodahurinyl-3-*O*-α-l-arabinopyranoside.

Compound 4 possessed the molecular formula of C_37_H_58_O_10_ based on the HREIMS at *m*/*z* 662.4058 [M]^+^ (calcd 662.4030). The NMR spectroscopic data for 4 resembled those of (16*S*,20*S*,24*R*)-12β-acetoxy-16,23-epoxy-24,25-dihydroxy-3β-(β-d-xylopyranosyloxy)-9,19-cyclolanost-22(23)-ene (AC),^24^ with major differences in sugar unit. In addition, the sugar moiety was attached to C-3 and determined as l-arabinose by the same way as that of 1. A α-orientation of H-3, H-12, H-16, H-17, and CH_3_-21 were determined by ROESY couplings of H-3 with H-5, H-12, H-16, and H-17 with CH_3_-28, and H-20 with H-17, respectively. Whereas, a β-orientation of H-8 was deduced by the correlation of H-8/CH_3_-18. In addition, identical to that of AC (16*S*,20*S*,24*R*)-12β-acetoxy-16,23-epoxy-24,25-dihydroxy-3β-(β-d-xylopyranosylo-xy)-9,19-cyclolanost-22(23)-ene, the characteristic ROESY association of CH_3_-18/CH_3_-26 was observed in 4, indicating it shares the same configuration at C-24 as *R* in AC (As shown in Fig S100,† when configuration of C-24 is *S*, it is impossible to see the cross-peak of CH_3_-18/CH_3_-26). Thus, the structure of 4 was determined as 12β-acetoxy-22(23)-en-15-deoxy-isodahurinyl-3-*O*-α-l-arabinopyranoside.

Compound 5 was purified as white powder, with the molecular formula C_37_H_58_O_11_, given by the HREIMS ([M]^+^*m*/*z* 678.3990, calcd 678.3979). The IR spectrum showed the presence of hydroxyl (3431 cm^−1^), carbonyl (1730 cm^−1^) and olefinic (1629 cm^−1^) groups. The NMR data of aglycone part for 5 (Tables 1 and 2) were similar to those of actaeaepoxide-3-*O*-α-d-xylopyranoside.^25^ The main differences were that a methine (C-22) at *δ*_C_ 86.6 was absent, instead, there's another methylene (*δ*_C_ 42.1), and the upfield shifts of C-23, C-24, and C-25 by 3.1 ppm, 5.4 ppm and 8.6 ppm, respectively. These changes could be explained as that, in 5, a methylene replaced a methine at C-22, and two hydroxy groups instead of the ternary epoxy ring at C-23 and C-24. These deductions were further confirmed by the HMBC coupling of H-20 (*δ*_H_ 4.81)/C-22 (*δ*_C_ 42.1). The sugar unit was connected to C-3 and identified as l-arabinose using same approaches as those of 1. In addition, the orientations of core structure and the configuration of C-24 of 5 were determined on the basis of the ROESY associations as those of 4. Therefore, the structure of 5 was determined as 12β-acetoxy-23,24-dihydroxy-7(8)-en-15-deoxy-isodahurinyl-3-*O*-α-l-arabinopyranoside.

On the basis of the HRTOF-ESIMS peak at *m*/*z* 715.3666 [M + Na]^+^ (calcd 715.3670), the molecular formula of 6 was determined as C_37_H_56_O_12_. ^1^H NMR resonances due to a downfield shifted cyclopropane methylene at *δ*_H_ 0.57 (1H, d, *J* = 4.0 Hz) and 1.07 (1H, overlapped), an olefinic proton at *δ*_H_ 6.73 (brd, *J* = 6.3 Hz), an acetyl methyl group at *δ*_H_ 2.24, a secondary methyl signal at *δ*_H_ 1.11 (d, *J* = 6.2 Hz), six singlet methyl groups at *δ*_H_ 1.00–1.59, as well as an anomeric proton at *δ*_H_ 4.76 (1H, d, *J* = 7.1 Hz) were observed (Table 1), indicating 6 is a CTs glycoside with an acetoxy group and a double bond. The sugar unit was connected to C-3 and deduced as l-arabinose by using same approaches as those of 1. Comparison of NMR data of 6 and hydroxyshengmanol-7(8)-en-15-one-3-*O*-β-d-xylopyranoside^26^ revealed the aglycone part of the two compounds were identical, except for an acetoxy group substituted at C-12 in 6, which further supported by the HMBC correlation of H-12 (*δ*_H_ 5.49) and the ester carbonyl group (*δ*_C_ 170.7). A α-orientation of the substituents at C-3, and C-12 were determined by ROESY correlations of H-3/H-5 and H-12/CH_3_-28. Whereas, correlations of H-20/CH_3_-18 and H-23/H-20 indicated the β-orientation of H-23. The stereochemistry of C-24 was elucidated as *S* by comparison of coupling constant of H-24 with known compounds (*S*, *J* ≤ 2 Hz; *R*, *J* ≈ 6).^15,26^ The molecular formula C_30_H_44_O_7_ of 7 was deduce from its HRTOF-ESIMS at *m*/*z* 539.2988 [M + Na]^+^ (calcd 539.2985). The spectroscopic features of 7 were identical to 6 except for a carbonyl group and a hydroxy group at C-3 and C-12, respectively. HMBC correlation of CH_3_-29 (*δ*_H_ 1.11) with the carbonyl carbon (*δ*_C_ 214.6) and the upfield shift of C-12 by 4.8 ppm further confirmed these elucidations. Same orientations of H-8, H-12, H-17, and H-23, as well as the configuration of C-24 between 7 and 6 were determined on the basis of the ROESY associations and comparison of coupling constant of H-24 with known compounds. Thus, the structure of 6 and 7 were determined as 12β-acetoxy-7(8)-en-15-one-hydroxyshengmanol-3-*O*-α-l-arabinopyranoside and 12β-hydroxy-7(8)-en-3,15-dione-hydroxyshengmanol, respectively.

The molecular composition of compound 8, C_30_H_48_O_5_, was established by HREIMS ([M]^+^*m*/*z* 488.3499, calcd 488.3502), indicating 7 degrees of unsaturation. The 30 carbon signals of 8 were similar to the aglycone resonances of actaeaepoxide-3-*O*-β-d-xylopyranoside.^25^ The main differences were that no double bond at C-7 and C-8, and the absence of an acetoxy group at C-12 in 8. Moreover, ^1^H–^1^H COSY correlations of H-6 (each 1H, *δ*_H_ 0.75 and 1.56) with H-7 (each 1H, *δ*_H_ 1.03 and 1.57), H-11 (each 1H, *δ*_H_ 1.10 and 1.98) with H-12 (2H, *δ*_H_ 1.59) confirmed these deductions. The orientations of H-16, H-17, and H-22 were assigned as α by analysis of ROESY spectrum. Besides, the characteristic ROESY correlation of H-22/H-24 further suggested the configuration between ring E and the ternary epoxy ring as shown (Fig S100†). Therefore, the structure of 8 was determined as actaeaepol.

The molecular formula of compound 9 was determined as C_32_H_48_O_9_ from HRTOF-ESIMS at *m*/*z* 599.3188 [M + Na]^+^ (calcd 599.3196). In the ^1^H NMR spectrum (Table 1), signals due to an extremely downfield shifted cyclopropane methylene at *δ*_H_ 1.02 (1H, d, *J* = 3.4 Hz) and 2.01 (1H, d, *J* = 3.9 Hz), an anomeric proton at *δ*_H_ 4.82 (d, *J* = 7.0 Hz), an olefinic hydrogen atom at *δ*_H_ 5.21 (brd, *J* = 6.3 Hz), four tertiary methyl groups at *δ*_H_ 1.12–1.60, and a secondary methyl signal at *δ*_H_ 0.90 (d, *J* = 5.9 Hz), were observed. The ^1^H–^1^H COSY spectrum indicated that 9 had part structure of –CHCH(CH_3_)CH_2_– (for C-17, C-20 to C-22). Aforementioned data together with HMBC associations of H-22 (2H, *δ*_H_ 2.43 and 2.51) with the carbonyl carbon (*δ*_C_ 211.2) and the oxygenated carbon at *δ*_C_ 82.3 (C-24, d), and H-17 (*δ*_H_ 2.25) with the oxygenated carbon at *δ*_C_ 81.9 (C-16, s) and C-24, exhibited that 9 was a foetidonol-type CTs glycoside. The sugar unit was connected to C-3 and identified as l-arabinose using same ways as those of 1. Compound 9 had a similar structure as that of foetidonol-3-*O*-β-d-xylopyranoside,^27^ except for a double bond at C-7 and C-8, and a hydroxy group at C-11. The ^1^H–^1^H COSY coupling of H-6 and the olefinic proton at *δ*_H_ 5.21, and the HMBC correlation of H-11 (*δ*_H_ 4.60) and C-9, as well as the molecule weight further confirmed these elucidations. Therefore, the structure of 9 was determined as 11β-hydroxy-7(8)-en-foetidonol-3-*O*-α-l-arabinopyranoside.

The HREIMS of 10 exhibited a molecular ion at *m*/*z* 488.3508 [M]^+^ (calcd 488.3502) for the molecular formula of C_30_H_48_NO_5_. The 1D NMR spectroscopic data (Table 1) of 10 showed seven tertiary methyl groups at *δ*_H_ 0.92–1.52, and a secondary methyl signal at *δ*_H_ 0.91 (d, *J* = 6.9 Hz). These data indicated that 10 possessed one more tertiary methyl group in the skeleton than a usual CTs. In ^13^C NMR spectrum, a pair of tetrasubstituted olefinic carbons at *δ*_C_ 134.9 (C-8, s) and 136.0 (C-9, s), and the characteristic ketal signal for C-16 of cimigenol-type CTs at *δ*_C_ 112.7 (s) were observed. HMBC associations of H-7/C-8 (*δ*_C_ 134.9, s), H-11/C-9 (*δ*_C_ 136.0, s), H-1/CH_3_-19 (*δ*_C_ 19.6, q), and CH_3_-19/C-9 and C-10, located the double bond at C-8 and C-9, and the CH_3_-19 at C-10, respectively. The rest of NMR resonances of 10 were identical to those of cimigenol.^28^ Therefore, the structure of 10 was determined as 19β-methyl-8(9)-en-cimigenol.

Compound 11 gave a pseudo-molecular ion at *m*/*z* 657.3613 [M + Na]^+^ (calcd 657.3615) in the positive ion HRTOF-ESIMS, corresponding to the molecular formula C_35_H_54_NaO_10_, which is 2 Da less than that of 15,16-seco-14-formyl-16-oxo-hydroshengmanol-3-*O*-α-l-arabinopyranoside.^14^ When its spectroscopic data (Table 1 and 2) were compared with 15,16-seco-14-formyl-16-oxo-hydroshengmanol-3-*O*-α-l-arabinopyranoside, the resonances of a methylene and a methine were absent in 11, showing instead a pair of double bond at C-7 and C-8. The ^1^H–^1^H COSY coupling of H-6 and the olefinic proton at *δ*_H_ 5.25 further supported this deduction. Therefore, the structure of 11 was determined as 15,16-seco-14-formyl-16-oxo-7(8)-en-hydroshengmanol-3-*O*-α-l-arabinopyranoside.

Compound 12 was assigned a molecular formula C_37_H_54_O_10_ from its HREIMS at *m*/*z* 658.3707 [M]^+^ (calcd 658.3717). The spectroscopic features of 12 resembled to those of 15,23-*O*-diacetyl-7(8)-en-shengmanol-3-*O*-α-l-arabinopyranoside^29^ except for the substituent group at C-23. For 12, the oxygenated methine of C-23 (*δ*_C_ 72.3, d) was absent, showing instead a carbonyl carbon at *δ*_C_ 205.5. This difference was due to a carbonyl group at C-23 in 12, which confirmed by the HMBC correlations of H-22 and H-24 with carbonyl signal at *δ*_C_ 205.5. The sugar unit and the relative configuration of 12 were elucidated by the same ways as those of aforementioned compounds. Therefore, the structure of 12 was determined as 15α-acetoxy-23-oxo-7(8)-en-cimicidanol-3-*O*-α-l-arabinopyranoside.

### 2.2. Alteration of morphology and phenotype on PMA-induced THP-1 cells

The normal THP-1 cells are ball-shaped without adhering to the surfaces of the plastic culture plates.^11^ Cultured by adding 100 nM PMA for 24 h, the cells became flat and amoeboid in shape, and adhered to the dish bottom (Fig. S97A and B†). Moreover, the differentiation of monocyte to macrophage was determined on the basis of 97% of the PMA-induced THP-1 cells were CD68 positive and 33% of these cells were CD11b positive with flow cytometry analysis (Fig. S97C and D†).

### 2.3. Cytotoxic activities of compounds 1–12 on PMA-induced THP-1 cells

Before conducting further bioassays, the cytotoxicities of compounds 1–12 on PMA-induced macrophages were tested by MTT assay. As shown in Fig S98,† compounds 1, 2, 6, 10, and 12 indicated notable cytotoxic effect (25% to 75% inhibition) on the cell viability from the concentration of 25 μM. Thus, these molecules were discarded for further studies. Conversely, compounds 3–5, 7–9 and 11 were chose for successive investigations due to their negligible cytotoxicity even at 50 μM (about 10–15% reduction on the cell viability) (Fig. S98†). In addition, the experimental concentration range was set as10 to 50 μM in the present study.

### 2.4. Downregulation of mRNA expression of CD147 and MMPs in PMA-induced THP-1 cells by compounds 3–5, 7–9 and 11

**Fig. 3:**
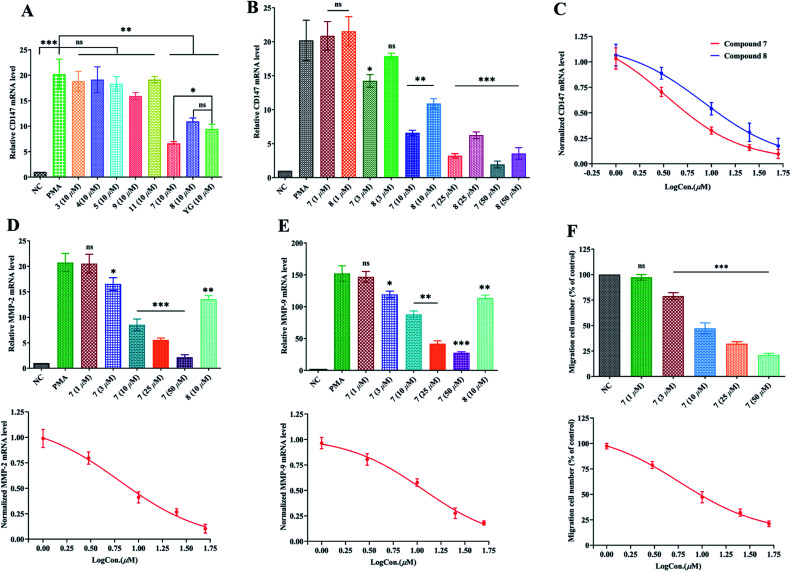
Suppression of compounds 3–5, 7–9 and 11 on mRNA expression of CD147, MMP-2, and MMP-9 and inhibition of compound 7 on the migration of PMA-induced THP-1 cells. (A) Suppression of 3–5, 7–9 and 11 on the mRNA expression of CD147; (B) Dose-related suppression of 7 and 8 on CD147 mRNA expression; (C) dose–response relationships for 7 and 8 to attenuate mRNA expression of CD147. The IC_50_ value of 7 and 8 is 3.38 μM and 8.25 μM, respectively. (D) Up: suppression of MMP-2 mRNA expression by 7 (1 μM to 50 μM) and 8 (10 μM). Down: dose–response relationship for 7 to attenuate mRNA expression of MMP-2. The IC_50_ value of 7 is 6.32 μM. (E) Up: suppression of MMP-9 mRNA expression by 7 (1 μM to 50 μM) and 8 (10 μM). Down: dose–response relationship for 7 to attenuate mRNA expression of MMP-9. The IC_50_ value of 7 is 11.57 μM. (F) Up: inhibition of 7 (1 μM to 50 μM) on the migration of PMA-induced THP-1 cells. Down: dose–response relationship for 7 to inhibit migration of PMA-induced THP-1 cells. The IC_50_ value of 7 is 5.87 μM. Relative quantification of gene expression was performed by the 2^−ΔΔCt^ method. The results showed the means ± SD from three independent experiments. Dose–response curve represents a fit to the Hill equation. Significant difference was compared with PMA group (A–E) or NC group (F): (*) *P* < 0.05, (**) *P* < 0.01, (***) *P* < 0.001; NC (in A, B, D, and E): Negative control (mRNA expression of undifferentiated THP-1 cells); PMA: PMA-differentiated THP-1 cells; YG: Yunnanterpene G. Expression of mRNA is defined as the change in mRNA copy numbers relative to NC group. The migration cell number of PMA-induced THP-1 cell group was defined as NC (F) and set to 100%.

Fig. 3A revealed that, the expression of CD147 was significantly augmented in PMA-induced THP-1 cells as compared with the NC group (*P* < 0.001) with q-PCR 2^−ΔΔCt^ method. Compounds 3–5, 7–9 and 11, and Yunnanterpene G (YG), the previously identified active compound (positive control), were firstly tested at 10 μM in the differentiated THP-1 cells for 24 hours. The aglycones (7 and 8) noticeably suppressed the mRNA expression of CD147 (*P* < 0.001). Conversely, the tested glycosides, 3–5, 7 and 11, were inactive. Of note, 7 is more potent than YG (*P* < 0.05), while, 8 showed same level of activity as YG. Dose–response studies further revealed that 7 and 8 down-regulated the mRNA expression with an IC_50_ value of 3.38 ± 0.27 μM and 8.25 ± 0.33 μM, respectively (Fig. 3B and C). Importantly, CD147 could regulate the expression of MMP-2 and MMP-9 in the activated macrophage.^4,11^ Thus, the effect of 7 and 8 on the mRNA production of these metalloproteinases were further investigated. As shown in Fig. 3D and E, 8 (10 μM) potently decreased the MMP-2 and MMP-9 mRNA expression by 35% (*P* < 0.01) and 25% (*P* < 0.01), respectively. In addition, the mRNA expression of MMP-2, and MMP-9 were significantly and dose-dependently down-regulated by 7, having an IC_50_ value of 6.32 ± 0.31 μM and 11.57 ± 0.23 μM, respectively.

### 2.5. Inhibition of PMA-induced THP-1 cells migration by compound 7

The enhanced migration or invasion of peripheral macrophages is a characteristic feature in pathological process of atherosclerosis.^4,5^ Because of compound 7 showed significant inhibition on the mRNA expression of CD147 and MMPs, the regulatory effect of this molecule on the migration of PMA-induced THP-1 cells was further studied by scratch wound assay. As a result, 7 (after 24 h incubation) potently and dose-related decreased the number of migrated cells with an IC_50_ value of 5.87 ± 0.27 μM (Fig. 3F and S99†).

## 3. Conclusion

As mentioned in the introduction, substances, which inhibit CD147 and MMPs expression may hold great potentials to prevent the development of atherosclerosis. Indeed, anti-atherogenic drugs, such as fluvastatin, attenuating the MMPs and EMMPRIN productions partially contribute to their clinical effects.^30–32^

Natural products (NPs) are important resources of active molecules for modern drug development.^33^ Previously, two undescribed CTs (YG and HC) from *C. foetida*, with notable inhibitions on CD147 and MMPs mRNA expression, were identified. Successive investigations on the aerial parts of *C. yunnanensis* led to characterize eleven new CTs, including nine glycosides (1–6, 9, and 11–12) and two aglycones (7 and 8), along with one undescribed lanostane-type triterpene (10). Compounds 7 and 8, two aglycones, showed noticeable inhibitory effects on the mRNA expression of CD147 and MMPs, as well as migration ability of the induced THP-1 cells. By contrast, all tested glycosides were inactive. It is worth noting that 7 is the most potent molecule among these four active CTs. Given the critical roles of CD147 and MMPs in stabilizing atherosclerotic plaques, 7 may have a promising effect in retarding the development of the vulnerability of the plaque, deserve to conduct more sophisticated animal studies in future.

In summary, our studies show that CTs, specially aglycones, are potential resources of anti-atherosclerosis bioactive agents and deserve further extensive exploration.

## 4. Experimental section

### 4.1. General experimental procedures

Column chromatography (CC) were conducted with Silica gel (200–300 mesh, Qingdao Marine Chemical, Inc.) and Lichroprep RP-18 (40–63 μm, Merck). Waters 2695 liquid chromatography system was applied to run Semipreparative HPLC with a YMC-Pack 10 mm × 250 mm column (Pro C18 RS). Thin-layer chromatography was carried out on precoated TLC plates (200–250 μm thickness, silica gel 60 F_254_, Qingdao Marine Chemical, Inc.). Bruker DRX-500 and Avance III-600 MHz spectrometers (Bruker, Zűrich, Switzerland) were used to record 1D and 2D NMR data with solvent signal as internal reference. ESIMS, HREIMS and HRESIMS were obtained from a Shimadzu LCMS-IT-TOF MS (Shimadzu, Kyoto, Japan), a Waters AutoSpec Premier P776 MS (Waters Corporation, Milford, USA) or an Agilent G6230 TOF MS (Agilent Technologies, Palo Alto, USA). Shimadzu IR-450 instrument was used to evaluate infrared spectra with KBr pellets. A JASCO P-1020 digital polarimeter was applied to test optical rotations, using MeOH as solvent. Quantitative-PCR (q-PCR) was conducted on ProFlex™ PCR system (Thermo Fisher, Shanghai, China).

### 4.2. Materials

#### 4.2.1 Plant materials

The aerial parts of *Cimicifuga yunnanensis* (4.7 kg) were collected from Litang county, Garze Tibetan autonomous prefecture, Sichuan, China, in October 2013. A voucher specimen (No. 20131007) has been deposited at Department of Respiratory Medicine, The Third People's Hospital of Kunming, Yunnan, 650041, P. R. China.

#### 4.2.2 Reagents

Human monocytic THP-1 cells were bought from Kunming Cell Bank, Kunming Institute of Zoology, Chinese Academy of Sciences. Penicillin/streptomycin was added to all reagents used for cell culture. RevertAid™ First Strand cDNA Synthesis Kit, DMEM/F12 medium, FBS (fetal bovine serum), and FITC-labeled anti-human CD68 and CD11b antibodies were purchased from Thermo Fisher (Shanghai, China). Restriction enzymes, Taq polymerase and PrimeScript RT reagent Kit were obtained from Takara Bio (Japan). Phorbol 12-myristate 13-acetate (PMA) was purchased from Sigma Chemical Co (St Louis, USA).

### 4.3. Extraction and isolation

The aerial parts of *Cimicifuga yunnanensis* (4.7 kg) was extracted at room temperature by MeOH (20 L, 3 times, 7 days each). The extract (489.7 g) was obtained after evaporation of MeOH under vacuum at 50 °C. The extract was divided by silica gel CC (12.0 kg, 30 × 200 cm) eluted with CHCl_3_–MeOH [100 : 0 (35 L), 50 : 1 (30 L), 10 : 1 (20 L), 5 : 1 (10 L), 0 : 100 (15 L)] to yield fractions A (52.6 g), B (67.4 g), C (55.7 g), D (29.2 g) and E (23.3 g). Sub-fractions (B.1–B.7) were further obtained by silica CC (4 kg, 10 × 150 cm), eluted with CHCl_3_–Me_2_CO from 40 : 1 gradient to 5 : 1. Compounds 7 (3.2 mg), 8 (2.4 mg) and 10 (2.2 mg) were purified from fraction B.4 (7.9 g) by RP-18 CC (300 g, 5 × 50 cm) eluting with MeOH–H_2_O from 60 : 40 to 100 : 0 and semipreparative HPLC (eluted with CH_3_CN–H_2_O, gradient from 60 : 40 to 80 : 20). Subsequently, further silica CC (2 kg, 10 × 150 cm) on fraction C, eluting with CHCl_3_–Me_2_CO from 20 : 1 gradient to 1 : 1, gave six sub-fractions (C.1–C.6). Another five sub-fractions (C.3.1–C.3.5) were obtained by RP-18 CC (1 kg, 10 × 50 cm), eluting with MeOH–H_2_O from 50 : 50 to 100 : 0. Fraction C.3.3 (7.7 g) gave compounds 1 (2.7 mg), 2 (3.2 mg), 3 (2.1 mg), 4 (2.2 mg), 11 (2.1 mg), and 12 (3.1 mg) by RP-18 CC (250 g, 5 × 50 cm) eluting with MeOH–H_2_O from 60 : 40 to 75 : 25 and semipreparative HPLC (eluted with CH_3_CN–H_2_O, gradient from 50 : 50 to 65 : 35). Compound 5 (2.5 mg), 6 (2.3 mg), and 9 (3.7 mg) were purified from fraction C.3.4 (6.9 g) by successively silica gel CC (120 g, 5 × 40 cm, eluted with CHCl_3_–Me_2_CO from 10 : 1 to 2 : 1), and semipreparative HPLC (eluted with CH_3_CN–H_2_O, 65 : 35).

#### 4.3.1 7(8)-en-Acteol-3-*O*-α-l-arabinopyranoside (1)

White powder; [*α*]^19^_D_ = −97.3 (*c*0.16, MeOH); IR (KBr): *ν*_max_ 3443, 2963, 2876, 1733, 1632, 1453, 1378, 1251, 1084, 1029, 987 cm^−1^; ESIMS (positive) *m*/*z* 681 [M + Na]^+^; HREIMS (positive) *m*/*z* 658.3703 [M]^+^, calcd for C_37_H_54_O_10_, 658.3717; ^1^H NMR (500 MHz, C_5_D_5_N) and ^13^C NMR (150 MHz, C_5_D_5_N) data see Tables 1 and 2.

#### 4.3.2 7(8)-en-23-*epi*-Acteol-3-*O*-α-l-arabinopyranoside (2)

White powder; [*α*]^19^_D_ = −39.1 (*c*0.11, MeOH); IR (KBr): *ν*_max_ 3441, 2962, 2874, 1733, 1630, 1455, 1382, 1247, 1070, 1028 cm^−1^; ESIMS (positive) *m*/*z* 681 [M + Na]^+^; HREIMS (positive) *m*/*z* 658.3694 [M]^+^, calcd for C_37_H_54_O_10_, 658.3717; ^1^H NMR (500 MHz, C_5_D_5_N) and ^13^C NMR (150 MHz, C_5_D_5_N) data see Tables 1 and 2.

#### 4.3.3 12β-Acetoxy-16(17)-en-isodahurinol-3-*O*-α-l-arabinopyranoside (3)

White powder; [*α*]^20^_D_ = −10.9 (0.08, MeOH); IR (KBr) *ν*_max_: 3424, 2931, 2872, 1706, 1642, 1455, 1381, 1137, 946 cm^−1^; ESIMS (positive) *m*/*z* 717 [M + Na + H_2_O]^+^; HRTOF-ESIMS (positive) *m*/*z* 717.3823[M + Na + H_2_O]^+^, calcd for C_37_H_58_N_a_O_12_, 717.3826; ^1^H NMR (600 MHz, C_5_D_5_N) and ^13^C-DEPT (150 MHz, C_5_D_5_N) data see Tables 1 and 2.

#### 4.3.4 12β-Acetoxy-22(23)-en-15-deoxy-isodahurinol-3-*O*-α-l-arabinopyranoside (4)

White powder; [*α*]^19^_D_ = −28.4 (*c*0.14, MeOH); IR (KBr): *ν*_max_ 3440, 2932, 2870, 1734, 1628, 1452, 1383, 1244, 1072, 993 cm^−1^; ESIMS (positive) *m*/*z* 685 [M + Na]^+^; HREIMS (positive) *m*/*z* 662.4058 [M]^+^, calcd for C_37_H_58_O_10_, 662.4030; ^1^H NMR (600 MHz, C_5_D_5_N) and ^13^C NMR (150 MHz, C_5_D_5_N) data see Tables 1 and 2.

#### 4.3.5 12β-Acetoxy-24-hydroxy-7(8)-en-15-deoxy-isodahurinol-3-*O*-α-l-arabinopyranosi-de (5)

White powder; [*α*]^19^_D_ = −29.3 (*c*0.14, MeOH); IR (KBr): *ν*_max_ 3431, 2964, 2876, 1730, 1629, 1451, 1383, 1249, 1069, 1025, 987 cm^−1^; ESIMS (positive) *m*/*z* 701 [M + Na]^+^; HREIMS (positive) *m*/*z* 678.3990 [M]^+^, calcd for C_37_H_58_O_11_, 678.3979; ^1^H NMR (600 MHz, C_5_D_5_N) and ^13^C NMR (150 MHz, C_5_D_5_N) data see Tables 1 and 2.

#### 4.3.6 12β-Acetoxy-7(8)-en-15-one-hydroxyshengmanol-3-*O*-α-l-arabinopyranoside (6)

White powder; [*α*]^19^_D_ = −18.82 (*c*0.17, MeOH); IR (KBr) *ν*_max_: 3423, 2928, 2873, 1736, 1629, 1458, 1384, 1129, 945 cm^−1^; ESIMS (positive) *m*/*z* 715 [M + Na]^+^; HRTOF-ESIMS (positive) *m*/*z* 715.3666 [M + Na]^+^, calcd for C_37_H_56_NaO_12_, 715.3670; ^1^H NMR (600 MHz, C_5_D_5_N) and ^13^C-DEPT (150 MHz, C_5_D_5_N) see Tables 1 and 2.

#### 4.3.7 (23*R*,24*S*)-12β-Hydroxy-7(8)-en-3,15-dione-hydroxyshengmanol (7)

White powder; [*α*]^19^_D_ = −50.78 (*c*0.10, MeOH); IR (KBr) *ν*_max_: 3441, 2925, 2854, 1753, 1631, 1454, 1384, 1141, 997 cm^−1^; ESIMS (positive) *m*/*z* 539 [M + Na]^+^; HRTOF-ESIMS (positive) *m*/*z* 539.2988 [M + Na]^+^, calcd for C_30_H_44_NaO_7_, 539.2985; ^1^H NMR (600 MHz, C_5_D_5_N) and ^13^C-DEPT (150 MHz, C_5_D_5_N) see Tables 1 and 2.

#### 4.3.8 Actaeaepol (8)

White powder; [*α*]^24^_D_ = −304.0 (*c*0.10, MeOH); IR (KBr): *ν*_max_ 3424, 2925, 2873, 1450, 1384, 1068, 1037, 1008 cm^−1^; ESIMS (positive) *m*/*z* 511 [M + Na]^+^; HREIMS (positive) *m*/*z* 488.3499 [M]^+^, calcd for C_30_H_48_NO_5_, 488.3502; ^1^H NMR (500 MHz, C_5_D_5_N) and ^13^C NMR (150 MHz, C_5_D_5_N) see Tables 1 and 2.

#### 4.3.9 11β-Hydroxy-7(8)-en-foetidonol-3-*O*-α-l-arabinopyranoside (9)

White powder; [*α*]^19^_D_ = −40.37 (*c*0.09, MeOH); IR (KBr) *ν*_max_: 3420, 2928, 2855, 1721, 1632, 1451, 1384, 1139, 985 cm^−1^; ESIMS (positive) *m*/*z* 599 [M + Na]^+^; HRTOF-ESIMS (positive) *m*/*z* 599.3188 [M + Na]^+^, calcd for C_32_H_48_NaO_9_ 599.3196; ^1^H NMR (600 MHz, C_5_D_5_N) and ^13^C-DEPT (150 MHz, C_5_D_5_N) see Tables 1 and 2.

#### 4.3.10 19β-Methyl-8(9)-en-cimigenol (10)

White powder; [*α*]^19^_D_ = −38.4 (*c* 0.10, MeOH); IR (KBr): *ν*_max_ 3432, 2932, 2870, 1627, 1455, 1375, 1229, 1076, 1028, 973 cm^−1^; ESIMS (positive) *m*/*z* 511 [M + Na]^+^; HREIMS (positive) *m*/*z* 488.3508 [M]^+^, calcd for C_30_H_48_NO_5_, 488.3502; ^1^H NMR (500 MHz, C_5_D_5_N) and ^13^C NMR (150 MHz, C_5_D_5_N) see Tables 1 and 2.

#### 4.3.11 15,16-*seco*-14-Formyl-16-oxo-7(8)-en-hydroshengmanol-3-*O*-α-l-arabinopyrano-side (11)

White powder; [*α*]^24^_D_ = −20.22 (*c*0.15, MeOH); IR (KBr) *ν*_max_: 3424, 2968, 2877, 1715, 1634, 1454, 1382, 1141, 995 cm^−1^; ESIMS (positive) *m*/*z* 657 [M + Na]^+^; HRTOF-ESIMS (positive) *m*/*z* 657.3613 [M + Na]^+^, calcd for C_35_H_54_NaO_10_ 657.3615; ^1^H NMR (600 MHz, C_5_D_5_N) and ^13^C-DEPT (150 MHz, C_5_D_5_N) see Tables 1 and 2.

#### 4.3.12 15α-Acetoxy-7(8)-en-cimicidanol-3-*O*-α-l-arabinopyranoside (12)

White powder; [*α*]^19^_D_ = −46.4 (*c*0.11, MeOH); IR (KBr): *ν*_max_ 3452, 2967, 2882, 1749, 1454, 1380, 1233, 1063, 1030, 991 cm^−1^; ESIMS (positive) *m*/*z* 681 [M + Na]^+^; HREIMS (positive) *m*/*z* 658.3707 [M]^+^, calcd for C_37_H_54_O_10_, 658.3717; ^1^H NMR (500 MHz, C_5_D_5_N) and ^13^C NMR (150 MHz, C_5_D_5_N) data see Tables 1 and 2.

### 4.4. Cell culture and differentiation

Human monocytic THP-1 cells were cultured at 37 °C by DMEM/F12 medium supplemented with 10% fetal bovine serum and 1% penicillin/streptomycin in a humidified atmosphere of 5% CO_2_. Cells (5 × 10^5^ to 10^6^ per mL) were subjected to differentiation by 100 nM PMA for 24 h with DMEM/F12 serum medium. After incubation, the adherent cells were washed with DMEM/F12. Nonattached cells were removed by aspiration.

### 4.5. Flow cytometry analysis

The expression of CD68 and CD11b on surface of the differentiated THP-1 cells were tested by suing Flow cytometry. In a dark condition, cells (5 × 10^5^) were washed 3 times by phosphate-buffered saline (PBS) and then respectively treated with fluorescein isothiocyanate (FITC)-conjugated anti-CD68 antibody, and FITC-conjugated anti-CD11b antibody, for 20 minutes. Cells were washed by PBS and then tested on a PARTEC CyFlow® Cube flow cytometer. Data were processed by the CytExpert software.

### 4.6. MTT assay

The differentiated THP-1 cells were seeded onto gelatinized 96-well culture plates (5 × 10^5^ cells per mL, 0.1 mL per well), and incubated at 37 °C with 5% CO_2_ for 24 h. Then, 0.1 mL of DMEM/F12 with different concentrations of compounds 1–12 (0, 5, 10, 25, 50, 75, and 100 μM) were added to instead of the original medium, and cultured for another 48 h. Cell viability was evaluated by MTT assay: each well was added with 20 μL of MTT to a final concentration of 0.5 g L^−1^ for 4 h before using 150 μL DMSO to solubilize the reactive dye. Each well was recorded by a Bio-Rad microplate reader to absorbance value of 570 nm. All the experiments were repeated in triplicate.

### 4.7. Isolation of total RNA and RT-PCR

RevertAid™ First Strand cDNA Synthesis Kit was applied to extract total RNA of differentiated THP-1 cells treated with compounds 3–5, 9 and 11 (10 μM), along with 7 and 8 (1, 3, 10, 25, and 50 μM) for 24 h (5 × 10^5^ cells per mL) following the manufacturer's instructions. cDNA was synthesized from the isolated total RNA based on the instruction of the PrimeScript RT reagent Kit. Briefly, PrimeScript RT Enzyme Mix 1 (0.5 μL), 5 × PrimeScriptTM Buffer (2 μL), oligo dt Primer (0.5 μL) and Random 6 mers (0.5 μL) were gently mixed with 1 μg RNA from each sample to a reaction volume of 10 μL with RNase free water, then incubated for 15 minutes at 37 °C to activate the reverse transcriptase enzyme. Finally, the reaction was stopped by 85 °C for 5 seconds.

After reverse transcription, cDNA was used to carry out real-time quantitative RT-PCR on ProFlex™ PCR system by SYBR Premix Ex Taq (Takara). The final volume of TR-PCR reaction is 25 μL, containing 12.5 μL SYBR green master mix, 1 μL cDNA, 0.5 μL each forward and reverse primer, and 10.5 μL nuclease-free water. For information of primers see Table S1.† Thermal cycling conditions for all genes were as follows: template pre-denaturation (10 min at 95 °C), denaturation (15 seconds at 95 °C), annealing and extension (30 seconds at 60 °C) for 40 cycles. Internal reference is *GAPDH* mRNA, and fold changes of mRNA expression for each target relative to *GAPDH* were calculated by the 2^−ΔΔCt^ method. Expression of mRNA is determined as the change in mRNA copy numbers relative to negative control cells (undifferentiated THP-1 cells). All the experiments were carried out in triplicate.

### 4.8. Wound-healing migration assay

The differentiated THP-1 cells were seeded and grown into full confluence in 6 well plates. 2% FBS DMEM/F12 media was used to inactivated cell proliferation for 12 h, then wounded with pipette tips. Fresh DMEM/F12 medium with or without 1, 3, 10, 25, and 50 μM of 7 was added to the scratched monolayers. Images were took after 24 hours using a Nickon inverted microscope (magnification, 10×). The migration cell number of PMA-induced THP-1 cell group was defined as control. All the experiments were performed in triplicate.

### 4.9. Statistical analysis

Data are presented as mean ± SD. Statistical analysis of data was performed with Student's *t*-test. (*) *P* < 0.05, (**) *P* < 0.01, and (***) *P* < 0.001 are considered significant.

## Annotations for abbreviations

CD147: Extracellular matrix metalloproteinase inducer, EMMPRIN
MMP-2: Matrix metalloproteinase 2
MMP-9: Matrix metalloproteinase 9
PMA: Phorbol-12-myristate-13-acetate
q-PCR: Quantitative real-time PCR method
CVDs: Cardiovascular diseases
AMI: Acute myocardial infarction
UA: Unstable angina pectoris
CTs: 9,19-Cycloartane triterpenes
YG: Yunnanterpene
HC: 12β-Hydroxycimiacerol
NPs: Natural products

## Author contributions

Ni-Hong Lu: investigation; methodology; writing-original draft. Jie Li and Yong-Rui Yang: formal analysis; writing-original draft. Hong-Lu Liu: data curation. Ying-Rong Du: supervision; writing – review & editing.

## Conflicts of interest

The authors declare no competing financial interest.

## Supplementary Material

RA-011-D1RA07828C-s001
