# Supplementary material for: New triterpenes from Cimicifuga yunnanensis down-regulating the mRNA expression of CD147, MMP-2, and MMP-9
Source: RSC Adv. 2021 Nov 17;11(58):36978–88. doi: 10.1039/d1ra07828c (PMC9043592; doi:10.1039/d1ra07828c)
Supplement: RA-011-D1RA07828C-s001 [file RA-011-D1RA07828C-s001.pdf]

Electronic Supplementary Material (ESI) for RSC Advances.
This journal is © The Royal Society of Chemistry 2021

New Triterpenes from *Cimicifuga yunnanensis* Down-regulating the mRNA Expression of CD147, MMP-2, and MMP-9

Ni-Hong Lu, Jie Li, Yong-Rui Yang, Hong-Lu Liu, Ying-Rong Du*

Department of Respiratory Medicine, The Third People's Hospital of Kunming, Yunnan, 650041, People's Republic of China

Supporting Information List

Page 3-10, ^{1}H, ^{13}C, HSQC, HMBC, COSY, ROESY NMR spectra, IR spectrum and HREIMS experiment of compound **1**.

Page 11-18, ^{1}H, ^{13}C, HSQC, HMBC, COSY, ROESY NMR spectra, IR spectrum and HRESIMS experiment of compound **2**.

Page 19-26, ^{1}H, ^{13}C, HSQC, HMBC, COSY, ROESY NMR spectra, IR spectrum and HRESIMS experiment of compound **3**.

Page 27-34, ^{1}H, ^{13}C, HSQC, HMBC, COSY, ROESY NMR spectra, IR spectrum and HRESIMS experiment of compound **4**.

Page 35-42, ^{1}H, ^{13}C, HSQC, HMBC, COSY, ROESY NMR spectra, IR spectrum and HREIMS experiment of compound **5**.

Page 43-50, ^{1}H, ^{13}C, HSQC, HMBC, COSY, ROESY NMR spectra, IR spectrum and HREIMS experiment of compound **6**.

Page 51-58, ^{1}H, ^{13}C, HSQC, HMBC, COSY, ROESY NMR spectra, IR spectrum and HRESIMS experiment of compound **7**.

Page 59-66, ^{1}H, ^{13}C, HSQC, HMBC, COSY, ROESY NMR spectra, IR spectrum and HRESIMS experiment of compound **8**.

Page 67-74, ^{1}H, ^{13}C, HSQC, HMBC, COSY, ROESY NMR spectra, IR spectrum and HRESIMS experiment of compound **9**.

Page 75-83, ^{1}H, ^{13}C, HSQC, HMBC, COSY, ROESY NMR spectra, IR spectrum and HRESIMS experiment of compound **10**.

Page 84-91, ^{1}H, ^{13}C, HSQC, HMBC, COSY, ROESY NMR spectra, IR spectrum and HRESIMS experiment of compound **11**.

Page 92-99, ^{1}H, ^{13}C, HSQC, HMBC, COSY, ROESY NMR spectra, IR spectrum and HRESIMS

experiment of compound **12**.

Page 100, Figure S97: Cell morphology and expression of CD11b and CD68 of differentiated and undifferentiated THP-1 cells.

Page 101, Figure S98: Effects of compounds **1-12** on the viability of PMA-induced THP-1 cells.

Page 102, Figure S99: Representative pictures of the migration of PMA-induced THP-1 cells.

Page 103, Figure S100: Key ROESY correlations of compounds **4** (**4a**) and **8** (**8a**)

Page 103, Table S1: Primers' information for CD147 and MMPs in the present study.

Figure S1. ^{1}H NMR Spectrum of **1** in Pyridine-d_5

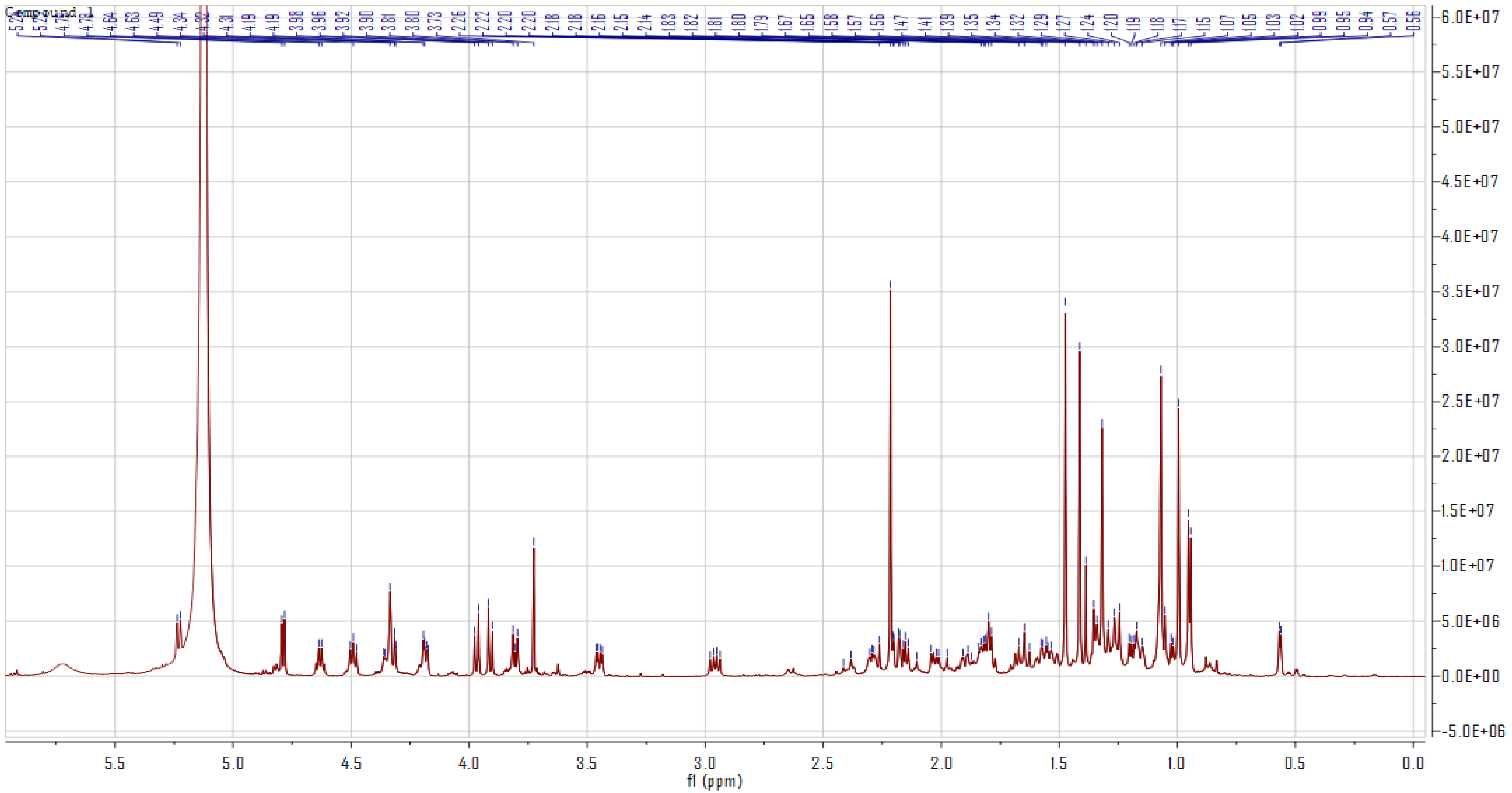

Figure S2. ^{13}C NMR Spectrum of **1** in Pyridine-d_5

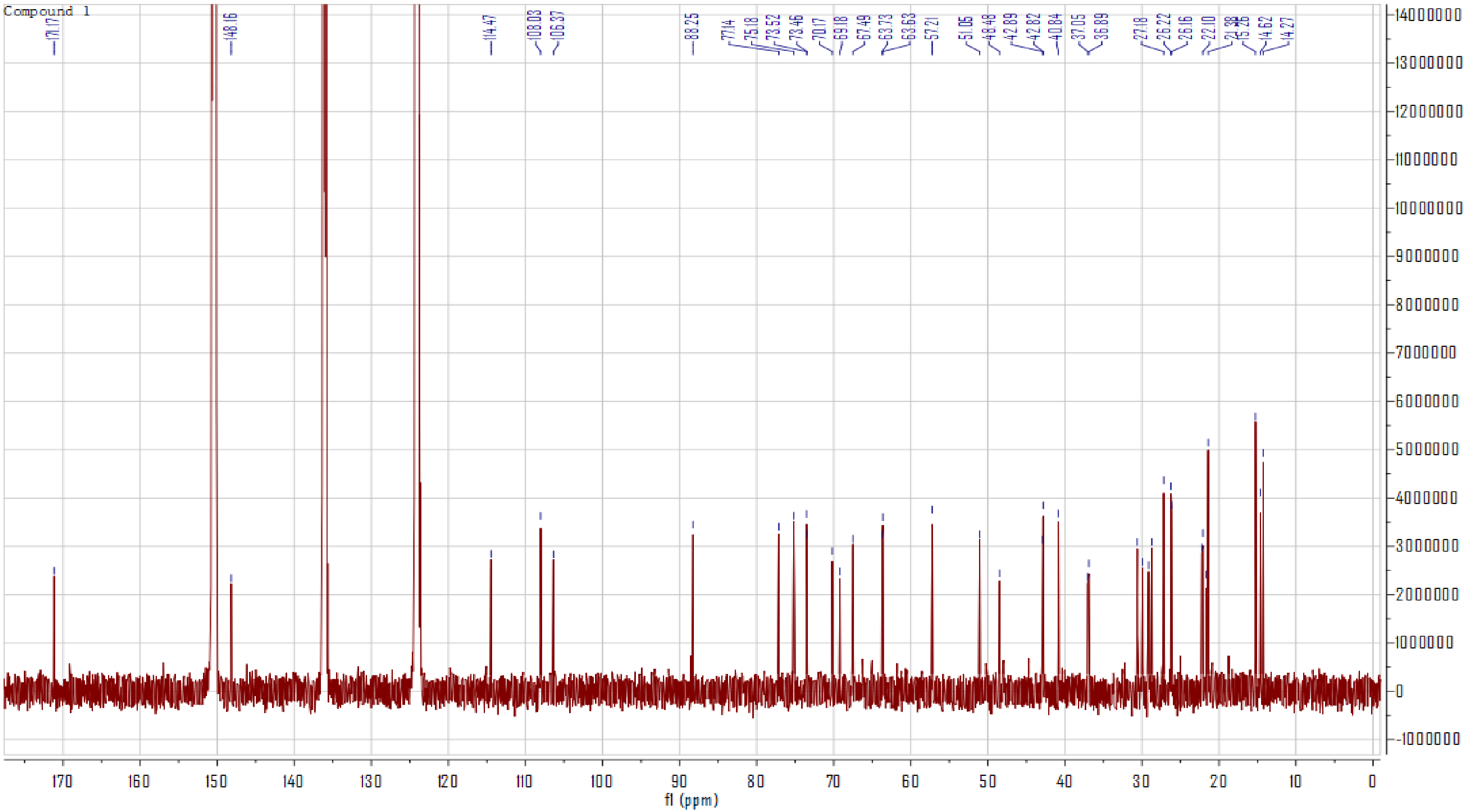

Figure S3. HSQC Spectrum of **1** in Pyridine-d_5

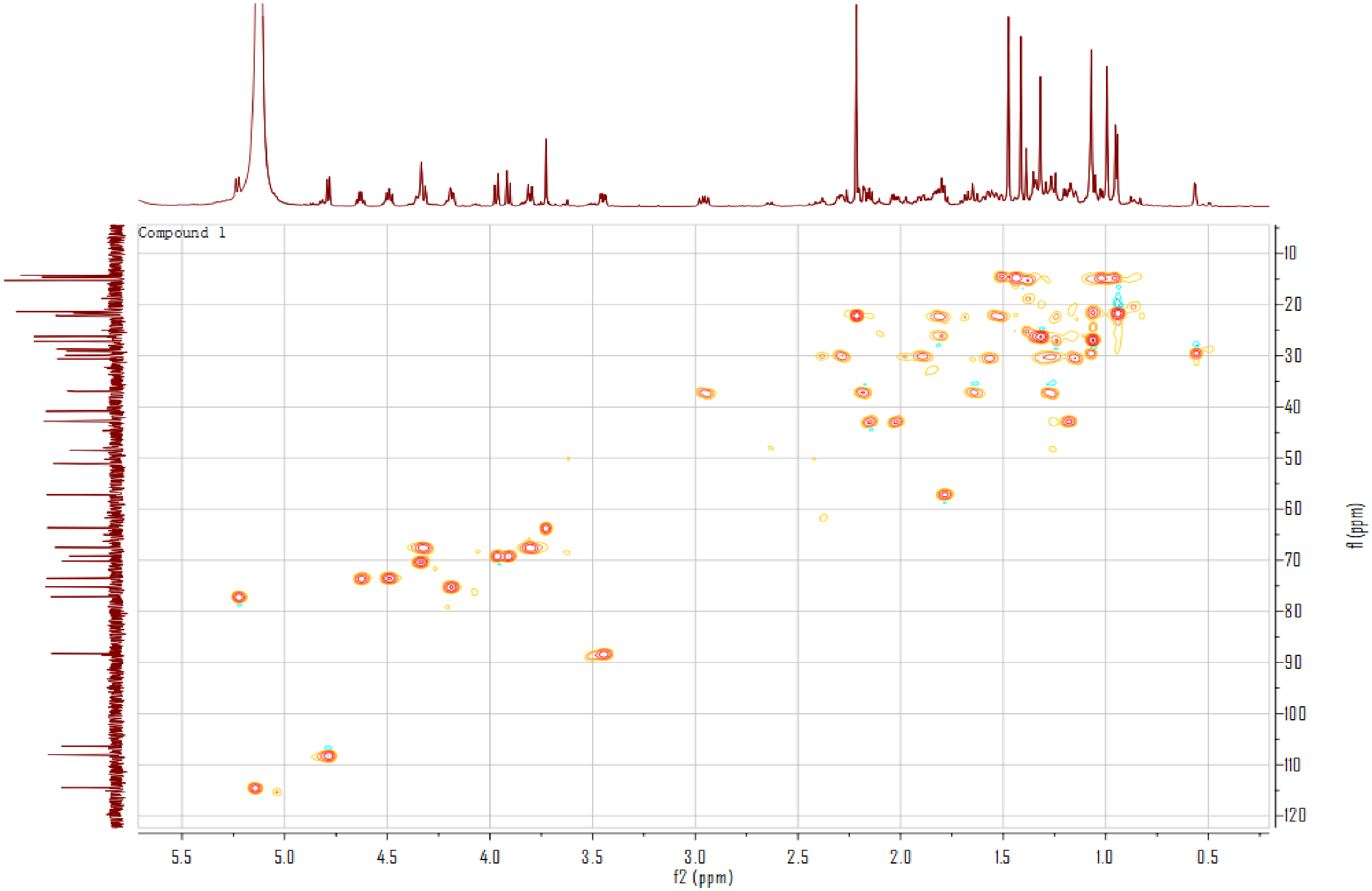

Figure S4. HMBC Spectrum of **1** in Pyridine-d_5

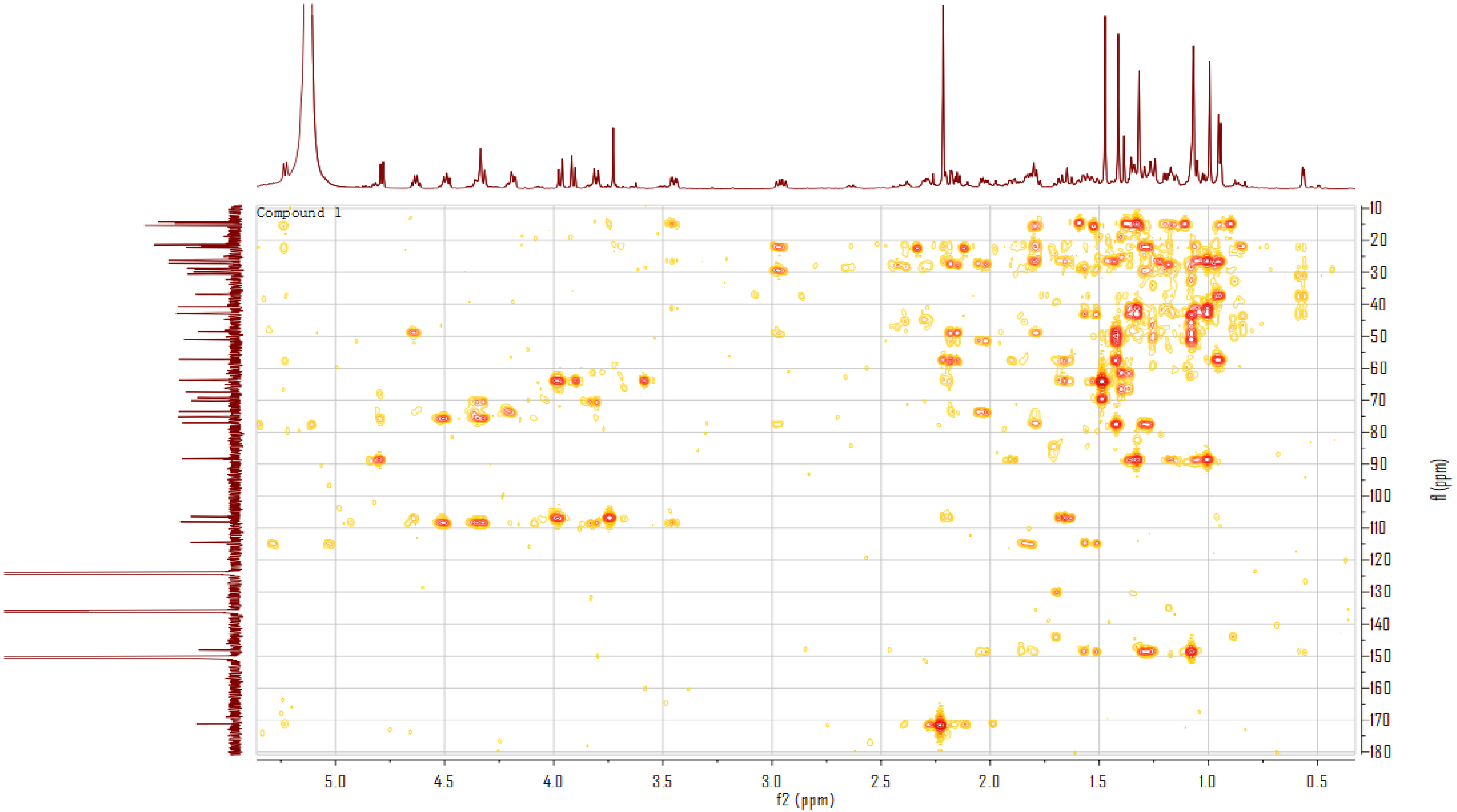

Figure S5. ^{1}H-^{1}H COSY Spectrum of **1** in Pyridine-d_5

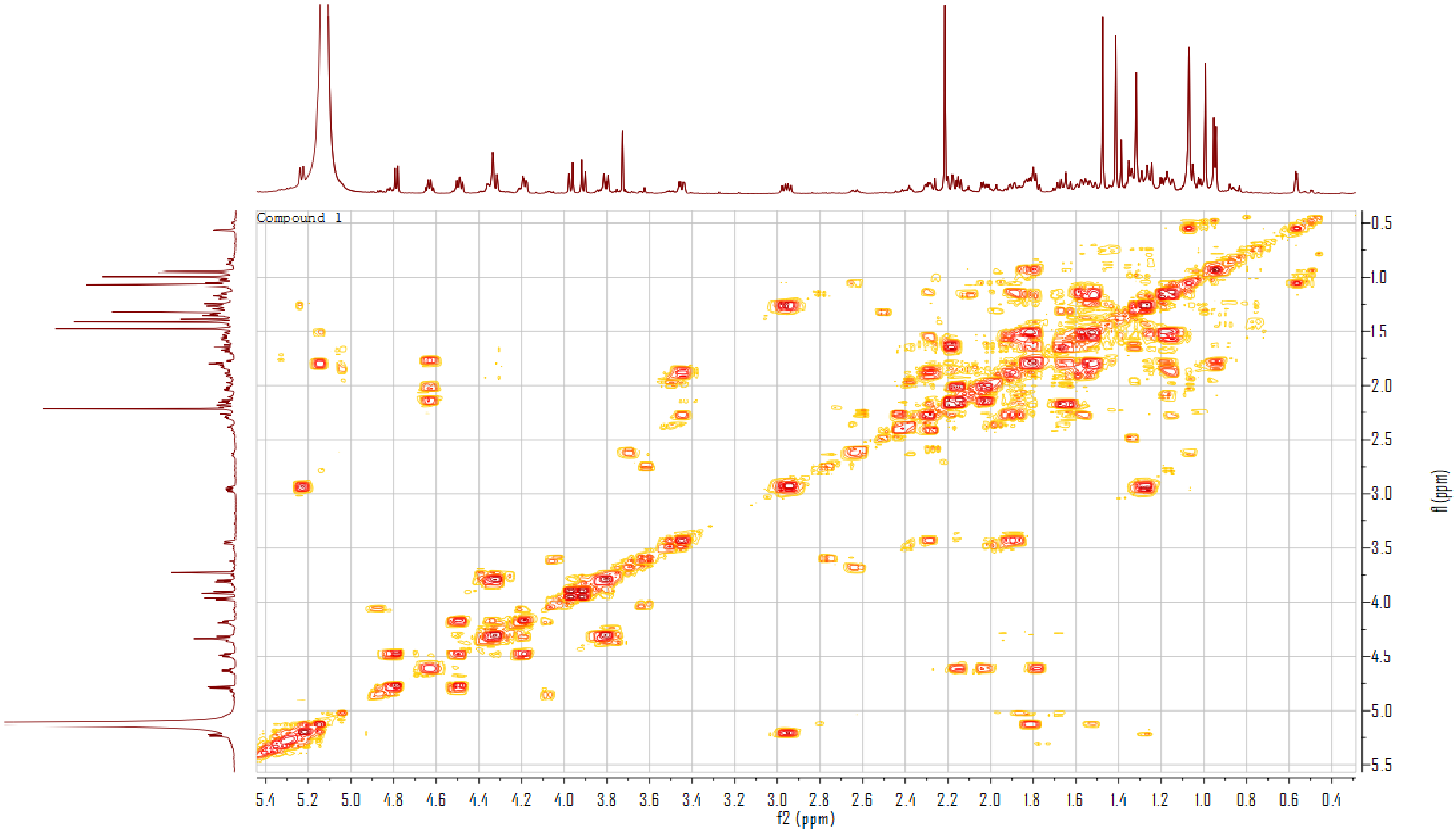

Figure S6. ROESY Spectrum of **1** in Pyridine-d_5

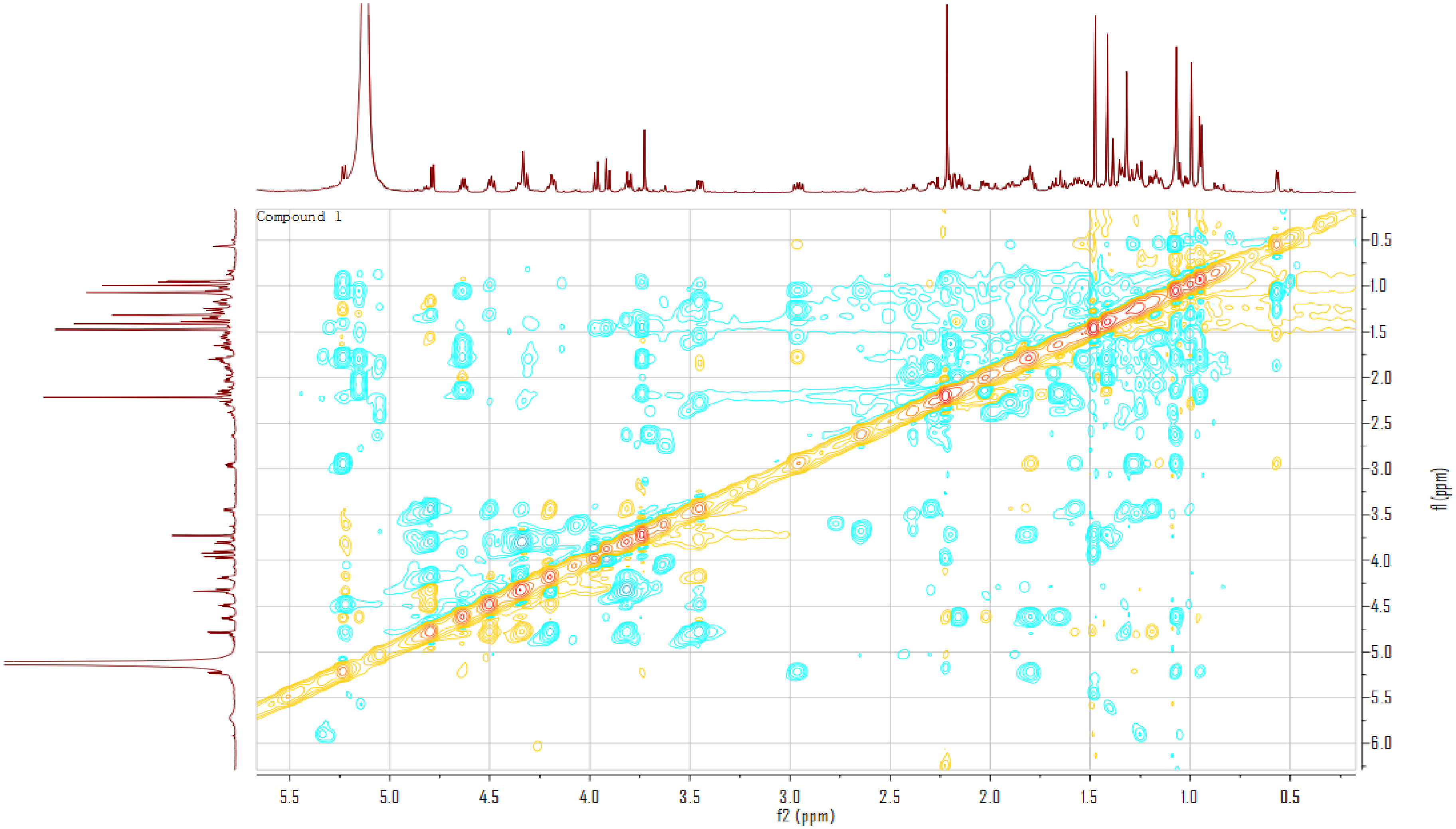

Figure S7. HREIMS of **1**

Elemental Composition Report

Page 1

Single Mass Analysis

Tolerance = 10.0 PPM / DBE: min = -10.0, max = 120.0
Selected filters: None

Monoisotopic Mass, Odd and Even Electron Ions
24 formula(e) evaluated with 1 results within limits (up to 51 closest results for each mass)
Elements Used:
C: 0-200 H: 0-400 O: 9-11

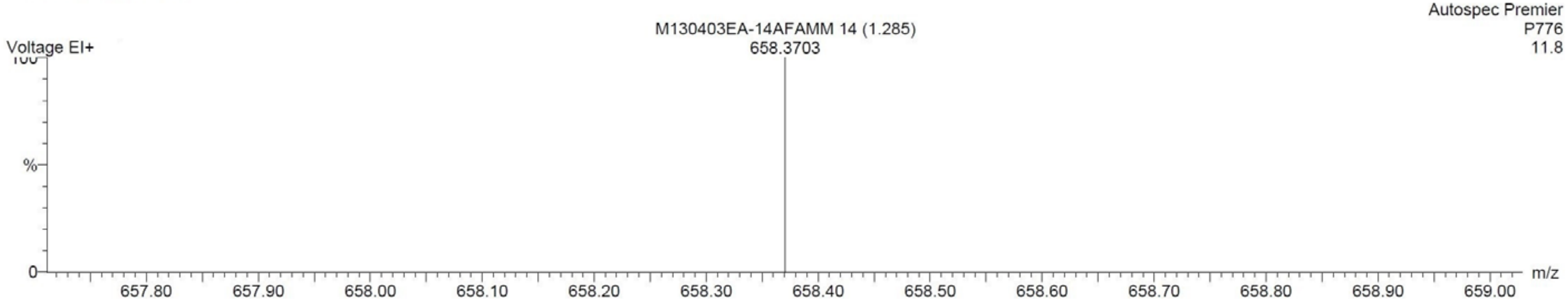

Minimum:				-10.0		
Maximum:		200.0	10.0	120.0		
Mass	Calc. Mass	mDa	PPM	DBE	i-FIT	Formula
658.3703	658.3717	-1.4	-2.1	11.0	5546027.5	C37 H54 O10

Figure S8. IR Spectrum of **1**

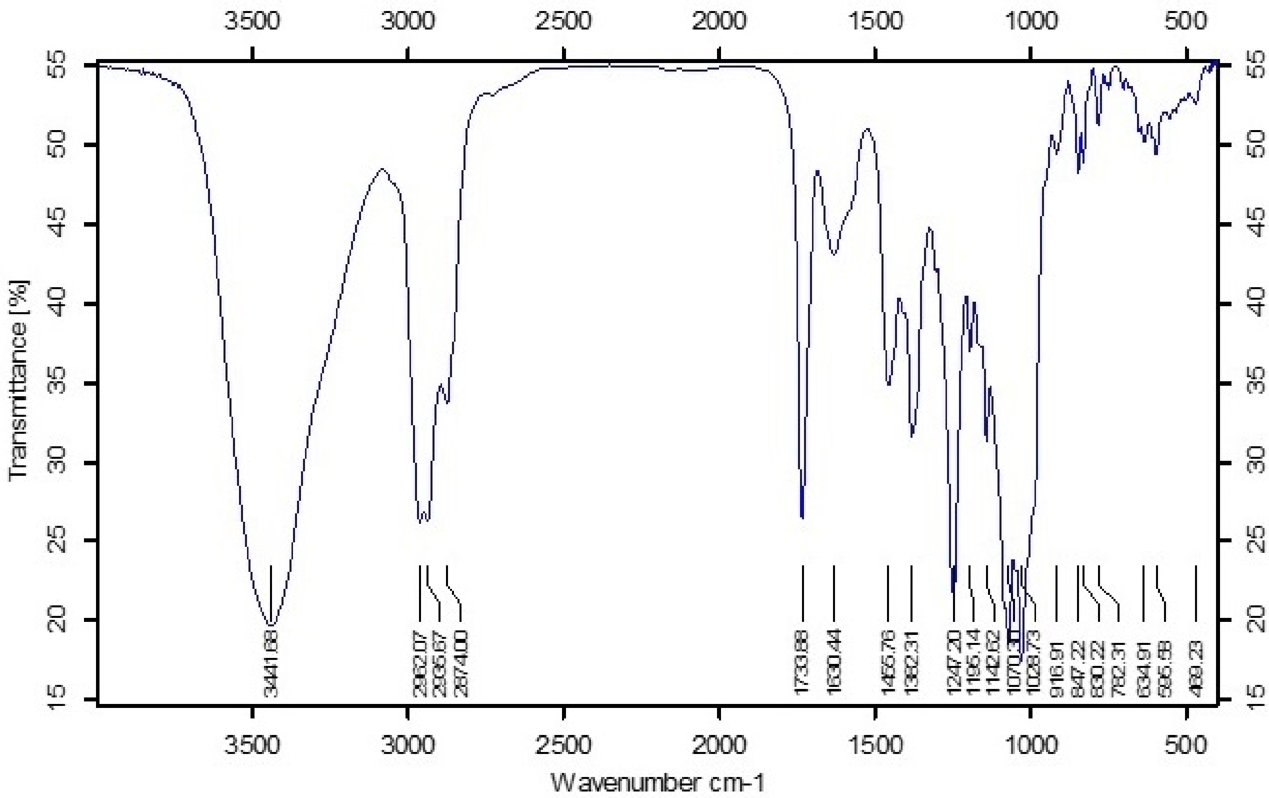

Figure S9. ^{1}H NMR Spectrum of **2** in Pyridine-d_5

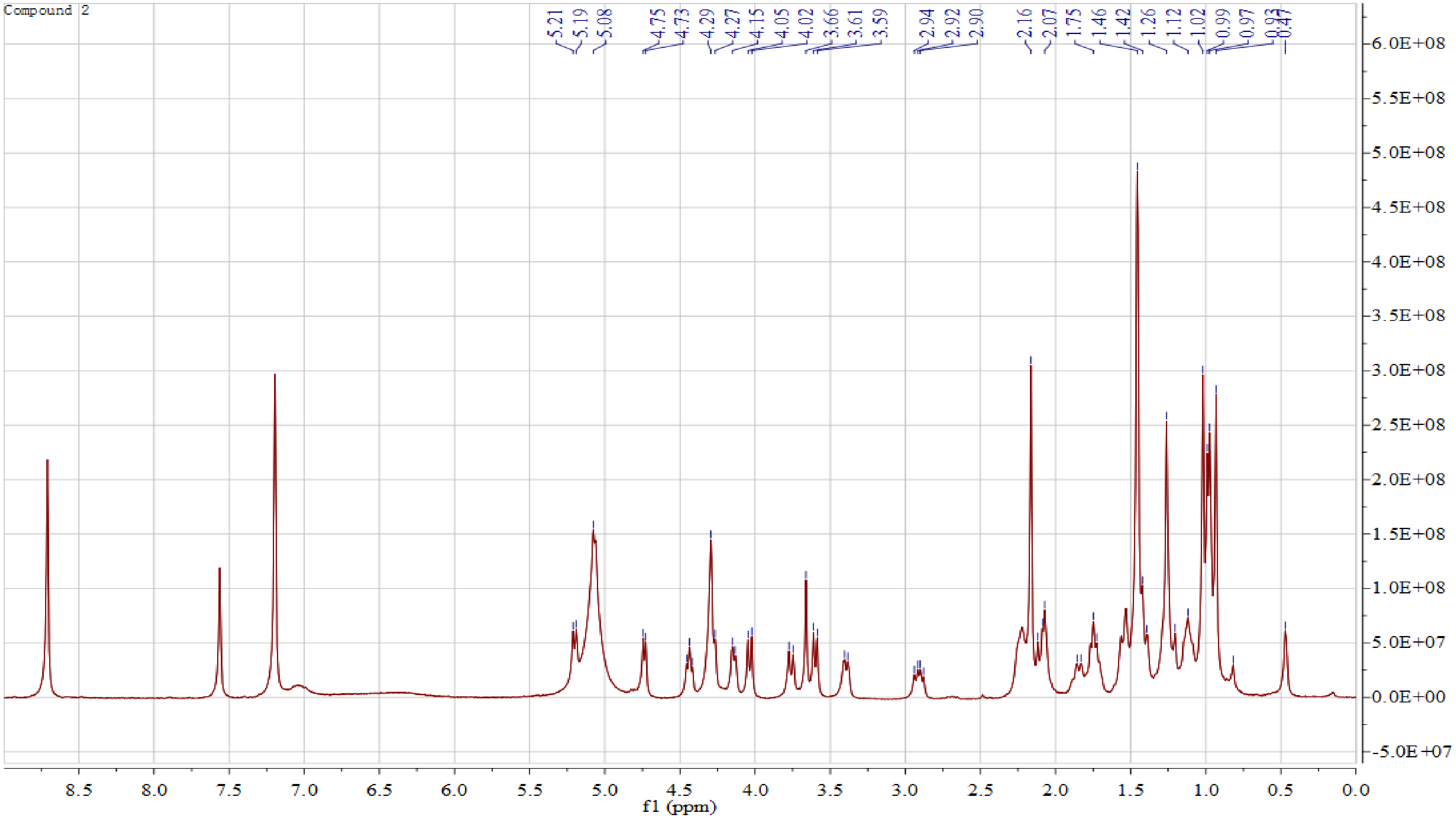

Figure S10. ^{13}C NMR Spectrum of **2** in Pyridine-d_5

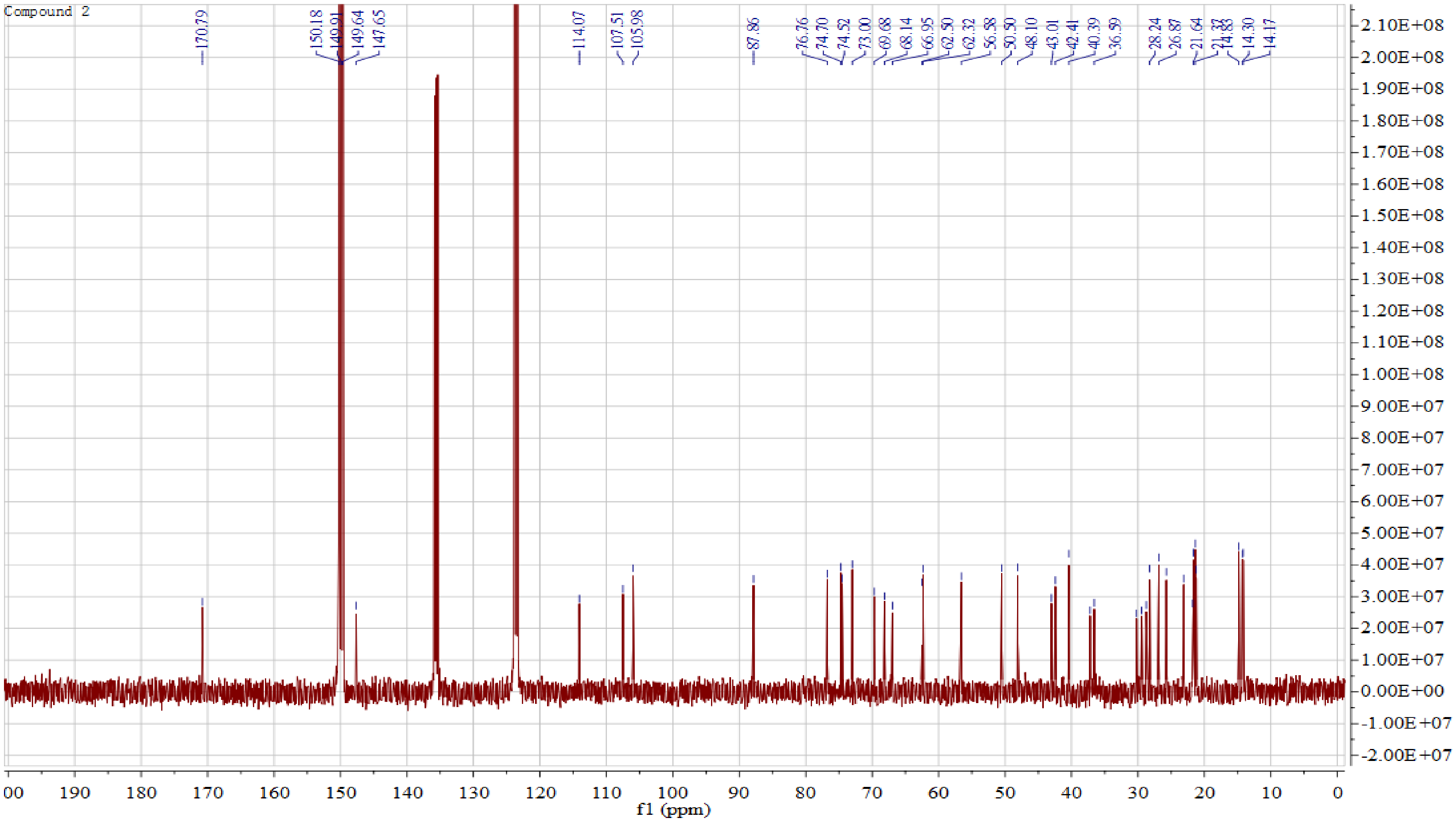

Figure S11. HSQC Spectrum of **2** in Pyridine-d_5

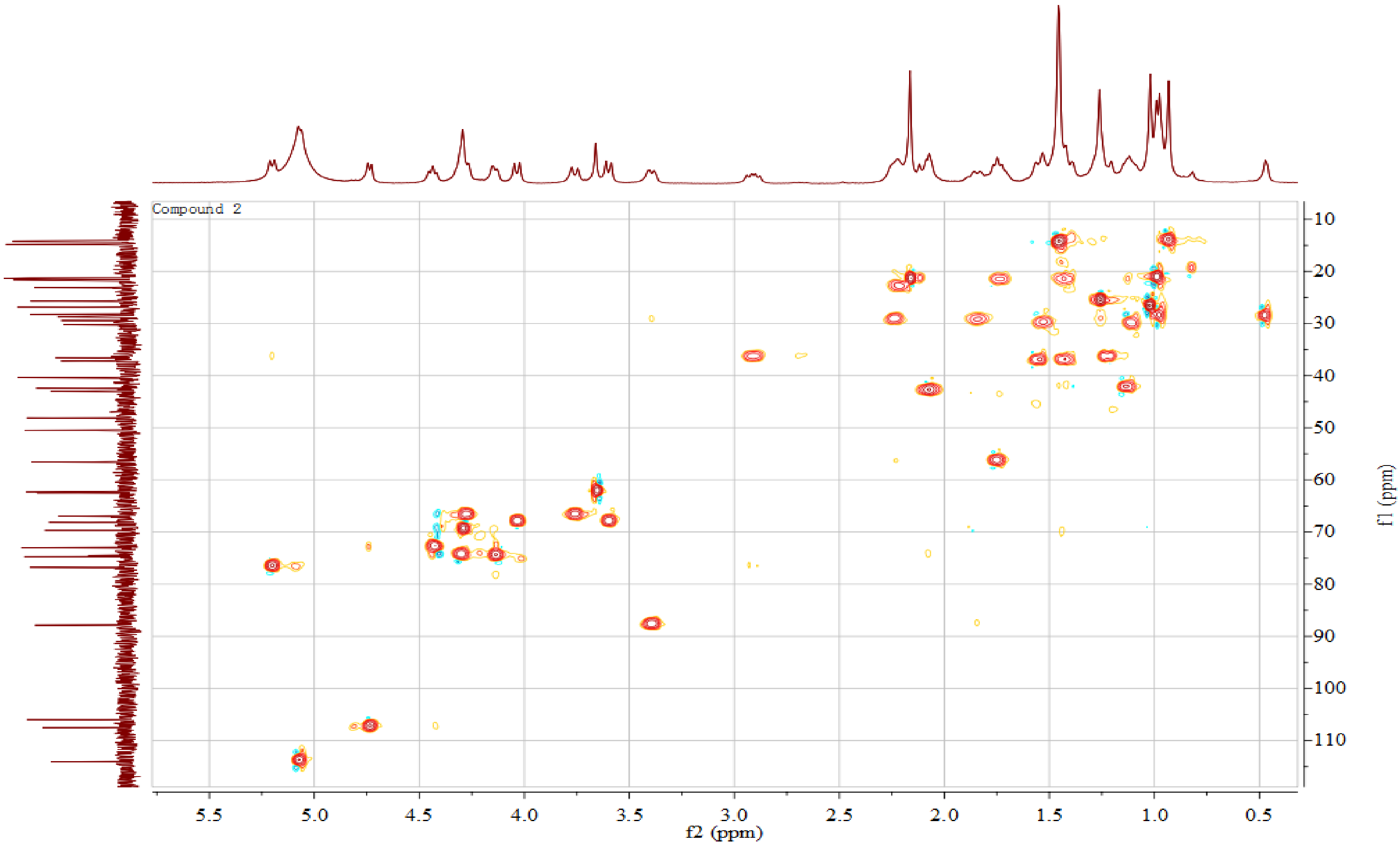

Figure S12. HMBC Spectrum of **2** in Pyridine-d_5

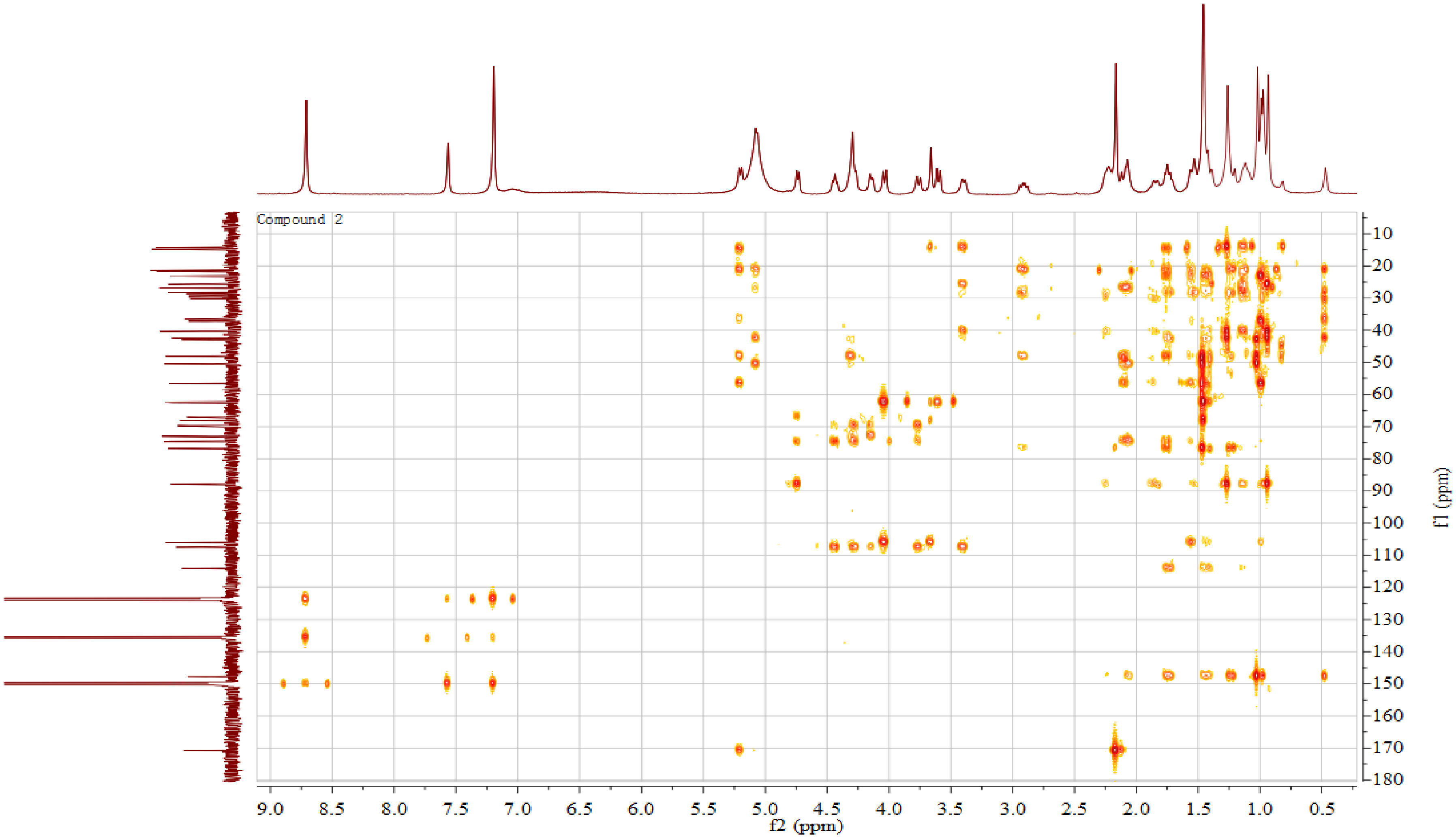

Figure S13. ^{1}H-^{1}H COSY Spectrum of **2** in Pyridine-d_5

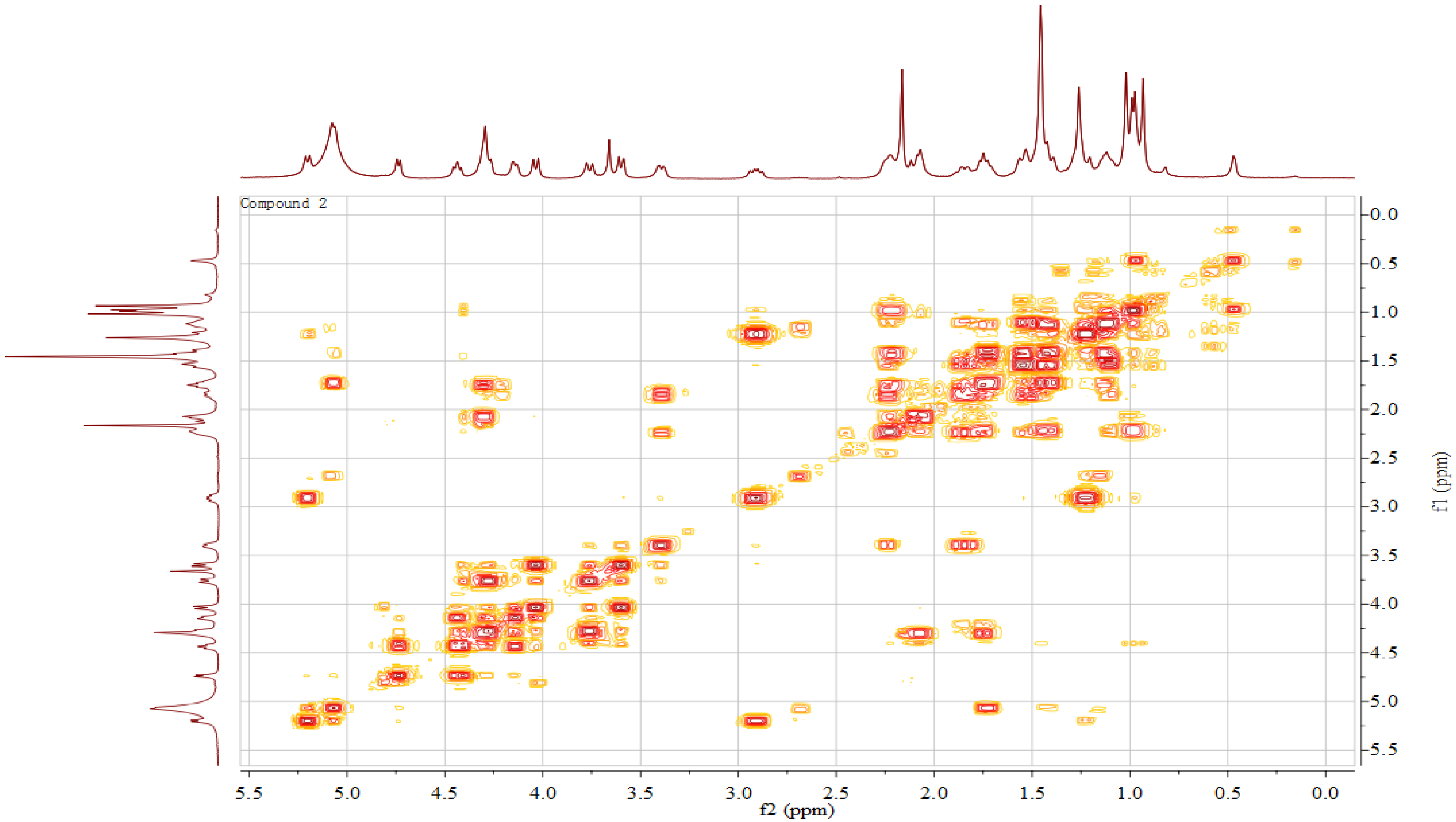

Figure S14. ROESY Spectrum of **2** in Pyridine-d_5

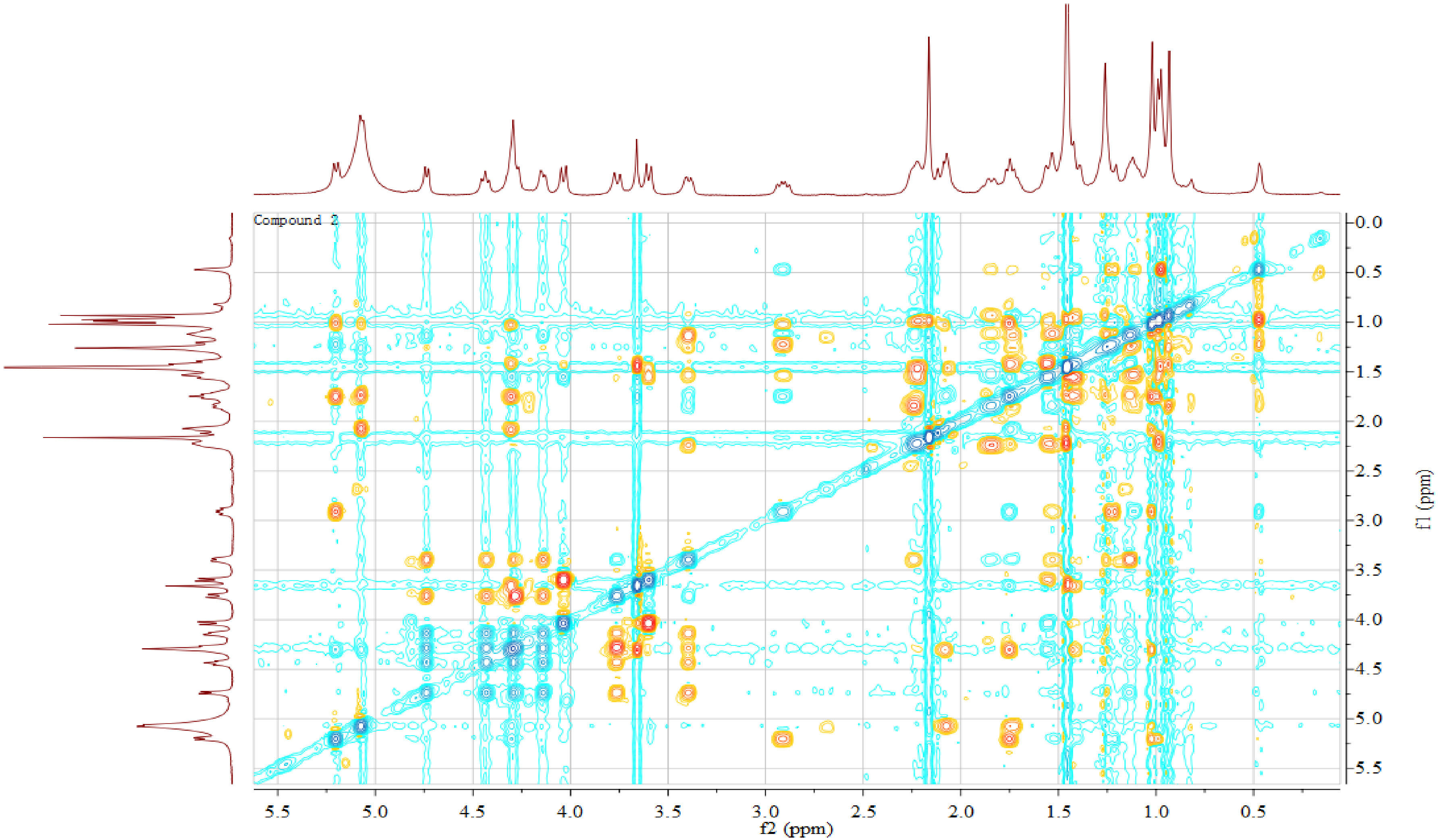

Figure S15. HRESIMS of **2**

Elemental Composition Report

Page 1

Single Mass Analysis

Tolerance = 10.0 PPM / DBE: min = -10.0, max = 120.0
Selected filters: None

Monoisotopic Mass, Odd and Even Electron Ions
24 formula(e) evaluated with 1 results within limits (up to 51 closest results for each mass)
Elements Used:
C: 0-200 H: 0-400 O: 9-11

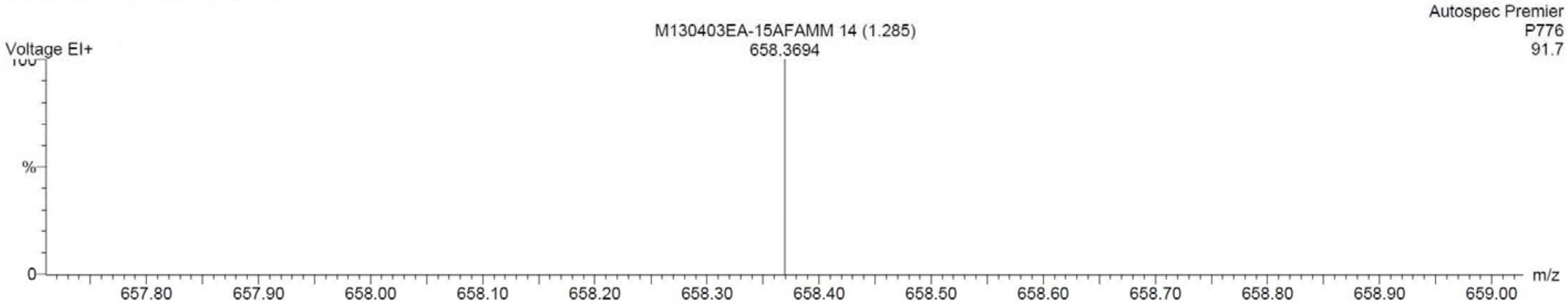

Minimum:				-10.0		
Maximum:		200.0	10.0	120.0		
Mass	Calc. Mass	mDa	PPM	DBE	i-FIT	Formula
658.3694	658.3717	-2.3	-3.5	11.0	5546066.0	C37 H54 O10

Figure S16. IR Spectrum of **2**

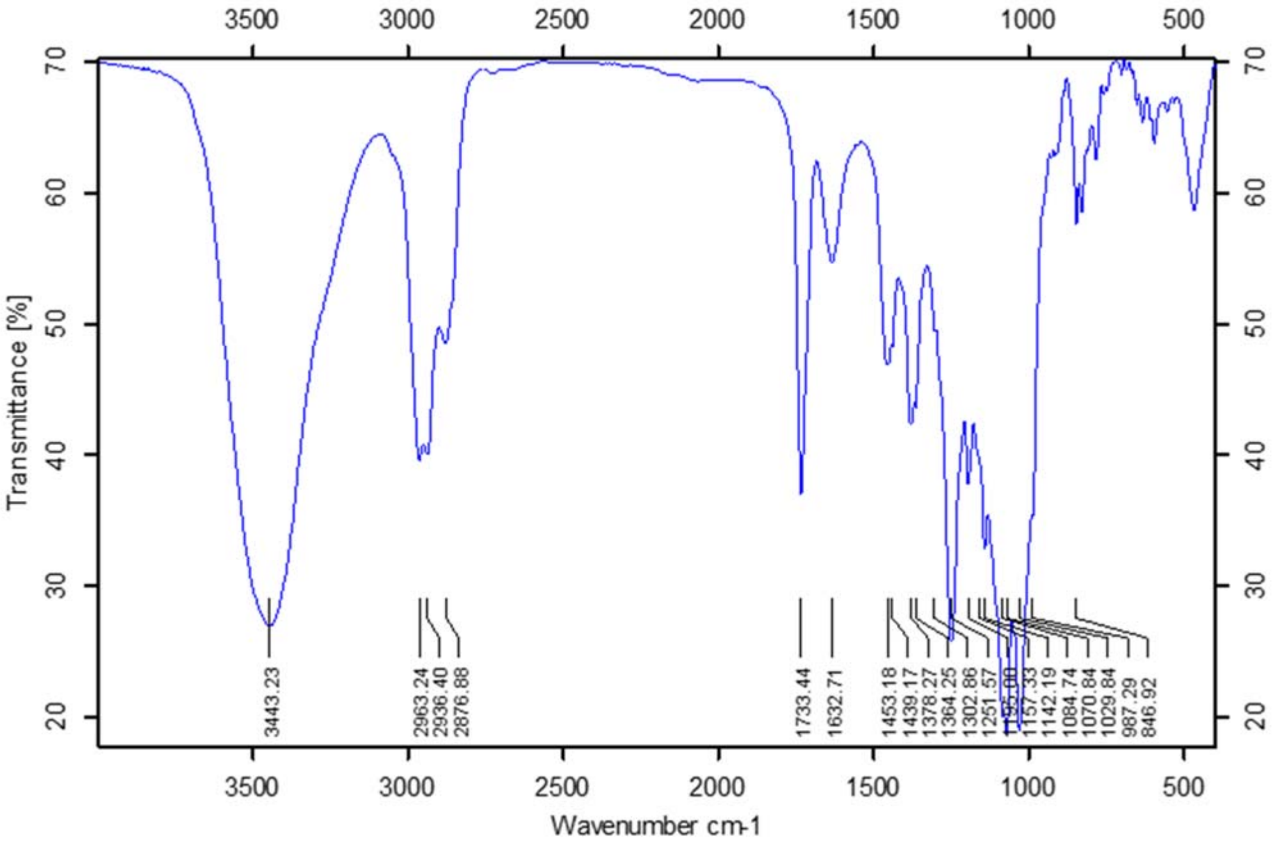

Figure S17. ^{1}H NMR Spectrum of **3** in Pyridine-d_5

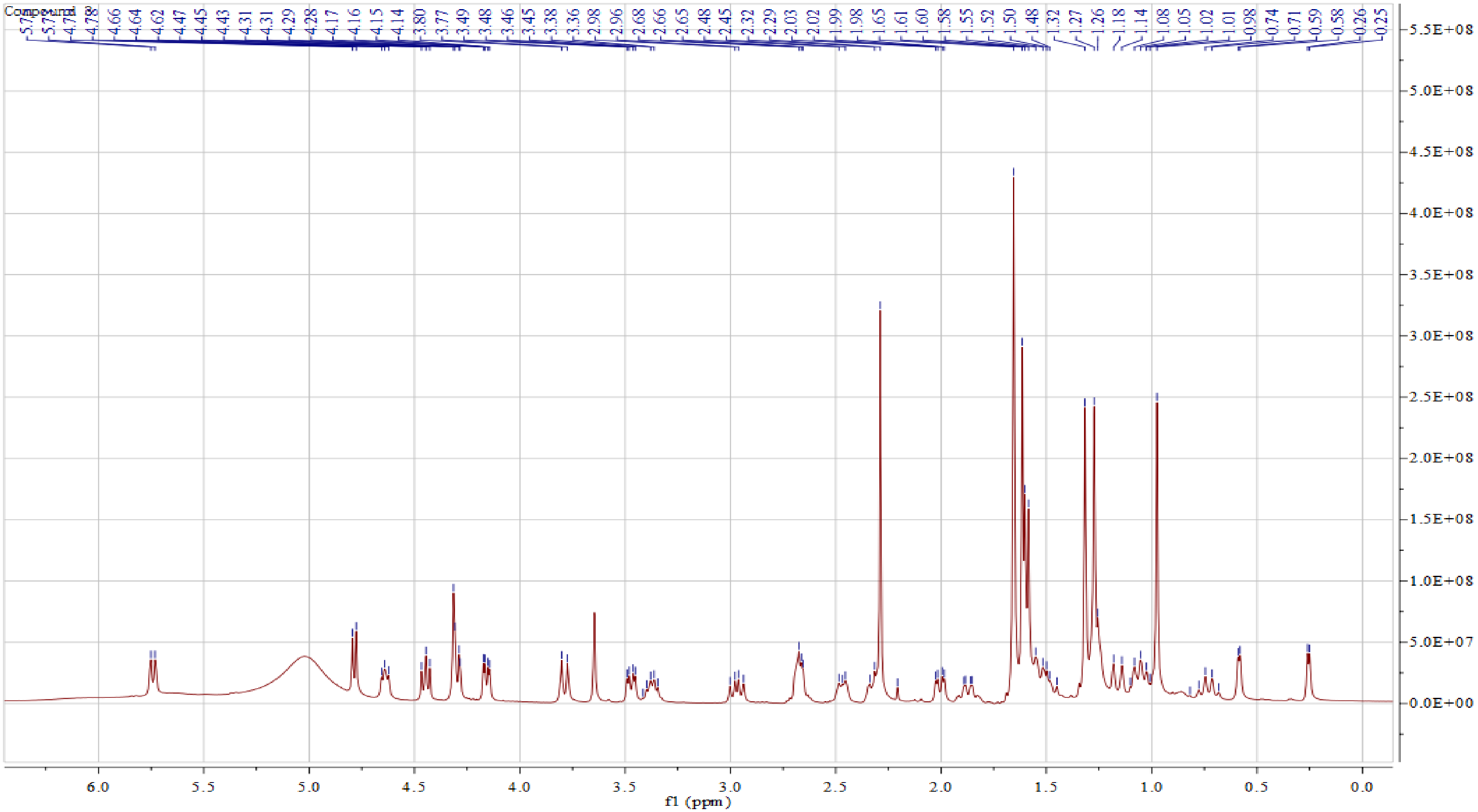

Figure S18. ^{13}C NMR Spectrum of **3** in Pyridine-d_5

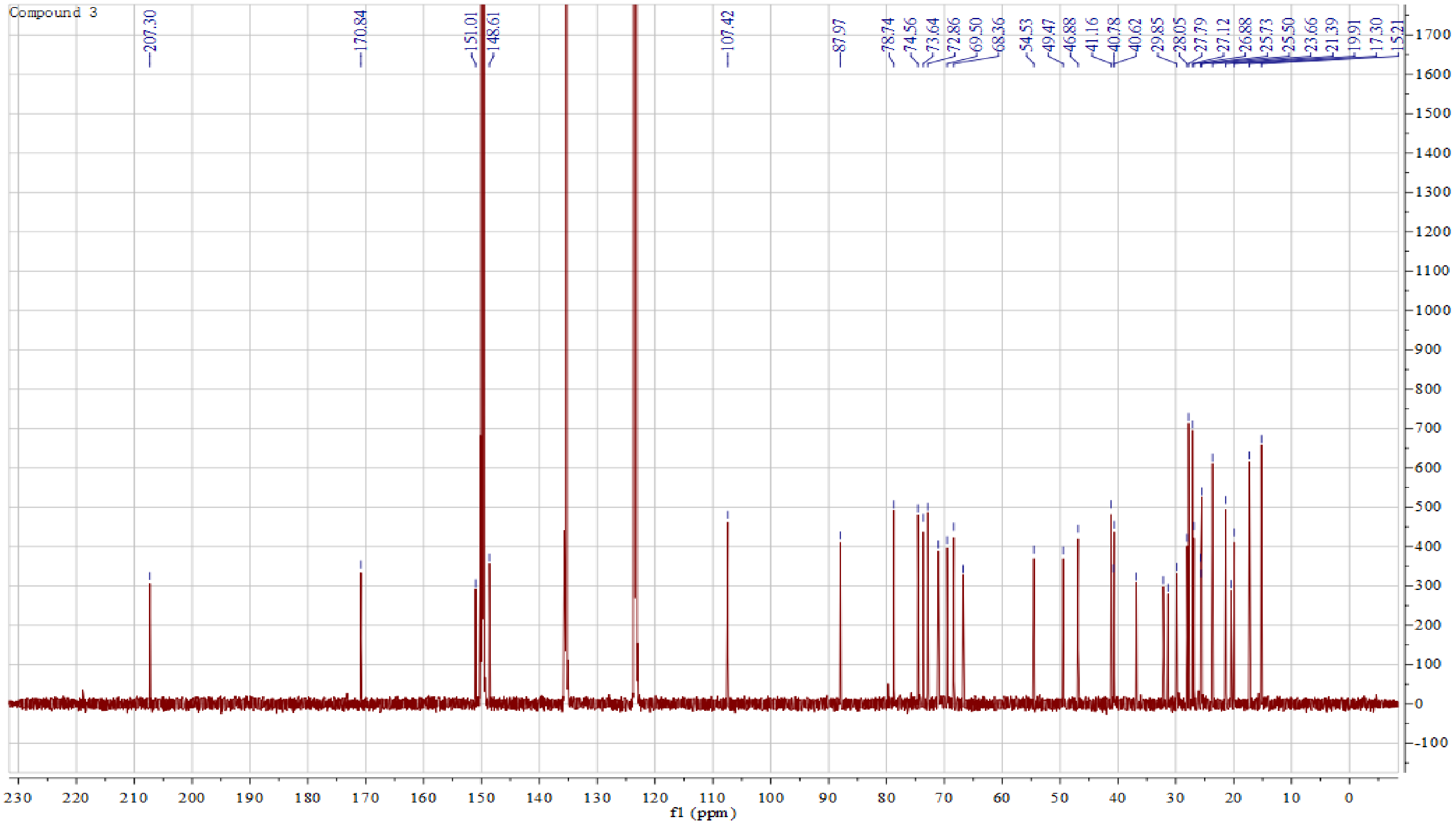

Figure S19. HSQC Spectrum of **3** in Pyridine-d_5

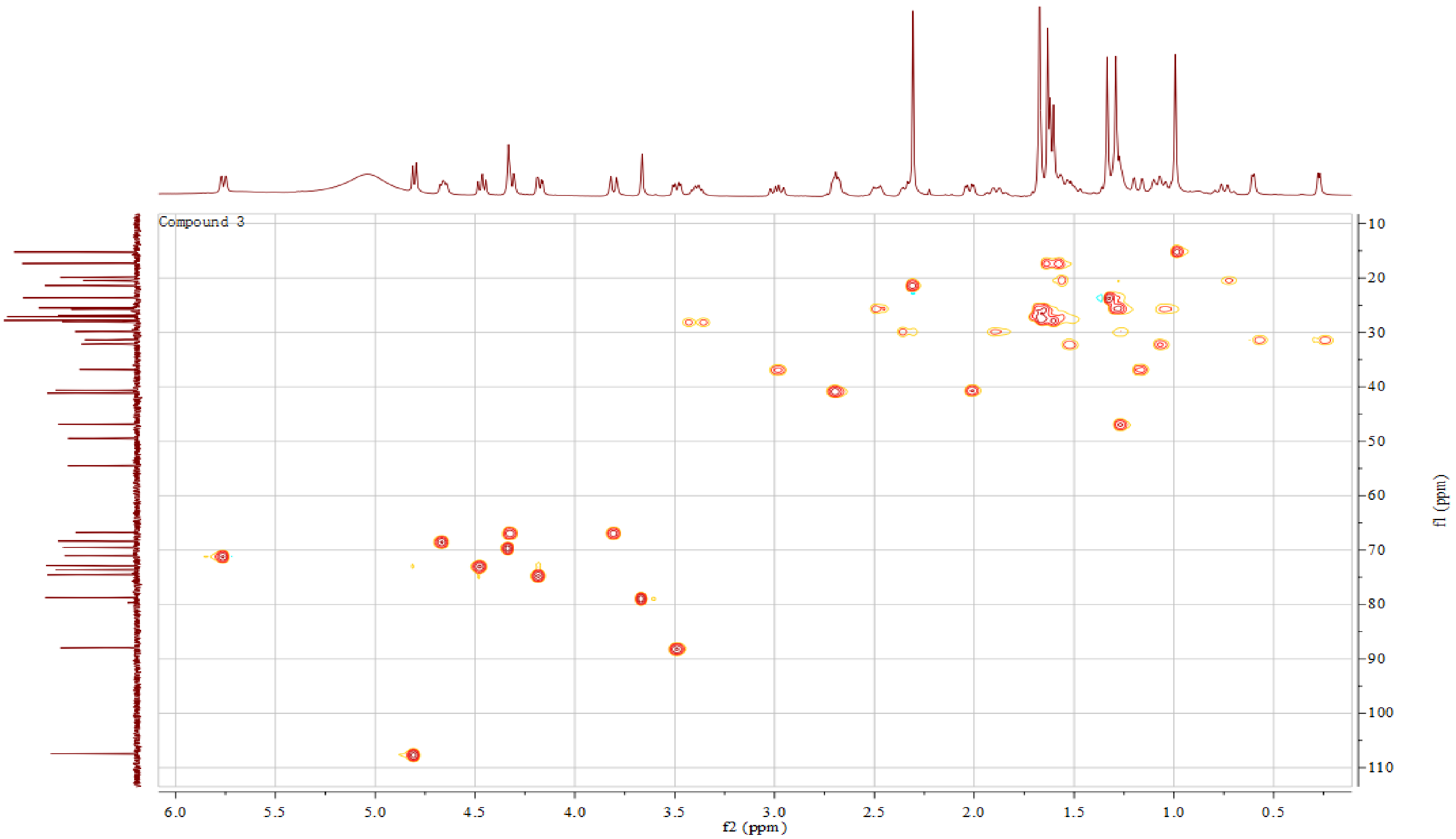

Figure S20. HMBC Spectrum of **3** in Pyridine-d_5

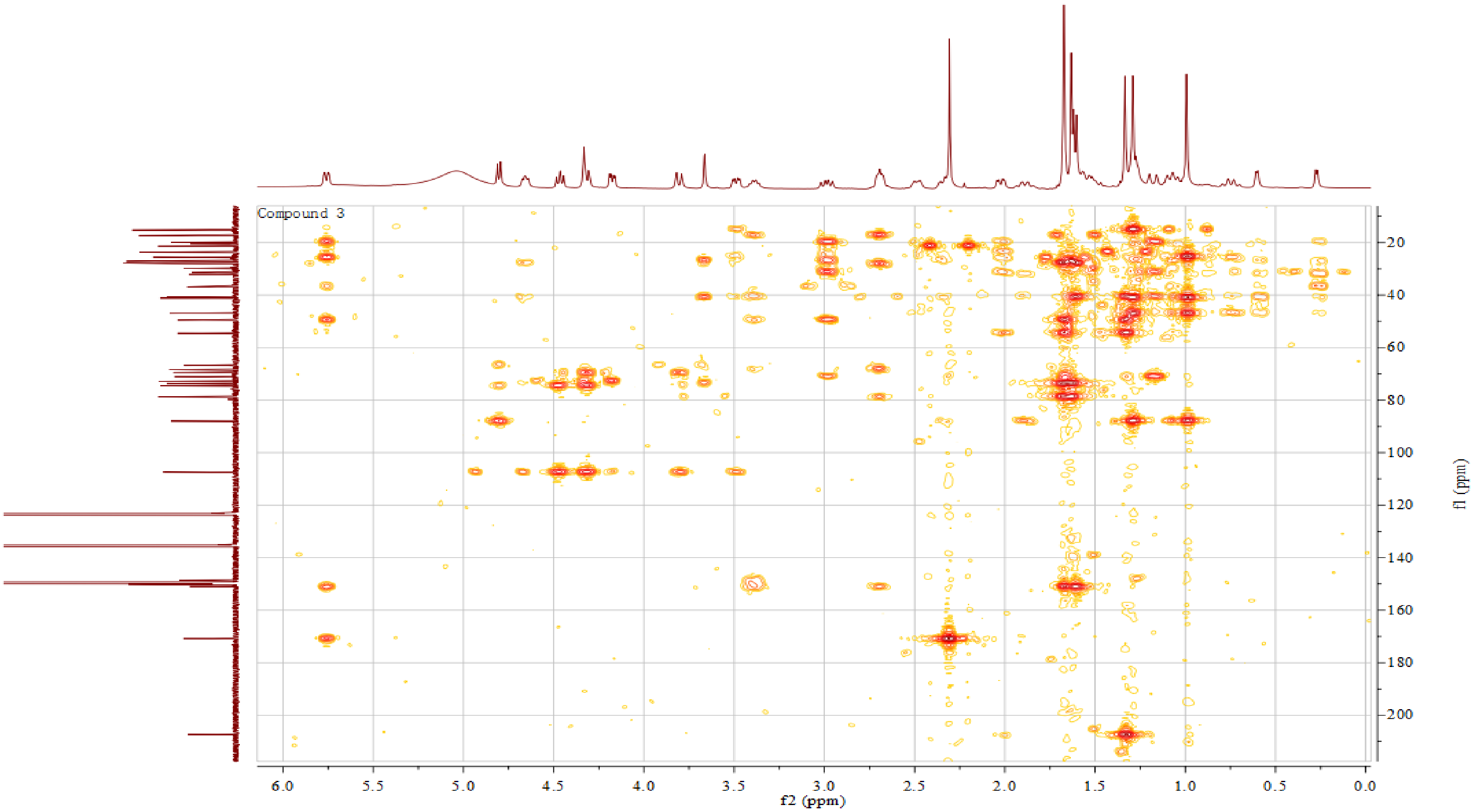

Figure S21. ^1H-^1H COSY Spectrum of **3** in Pyridine-d_5

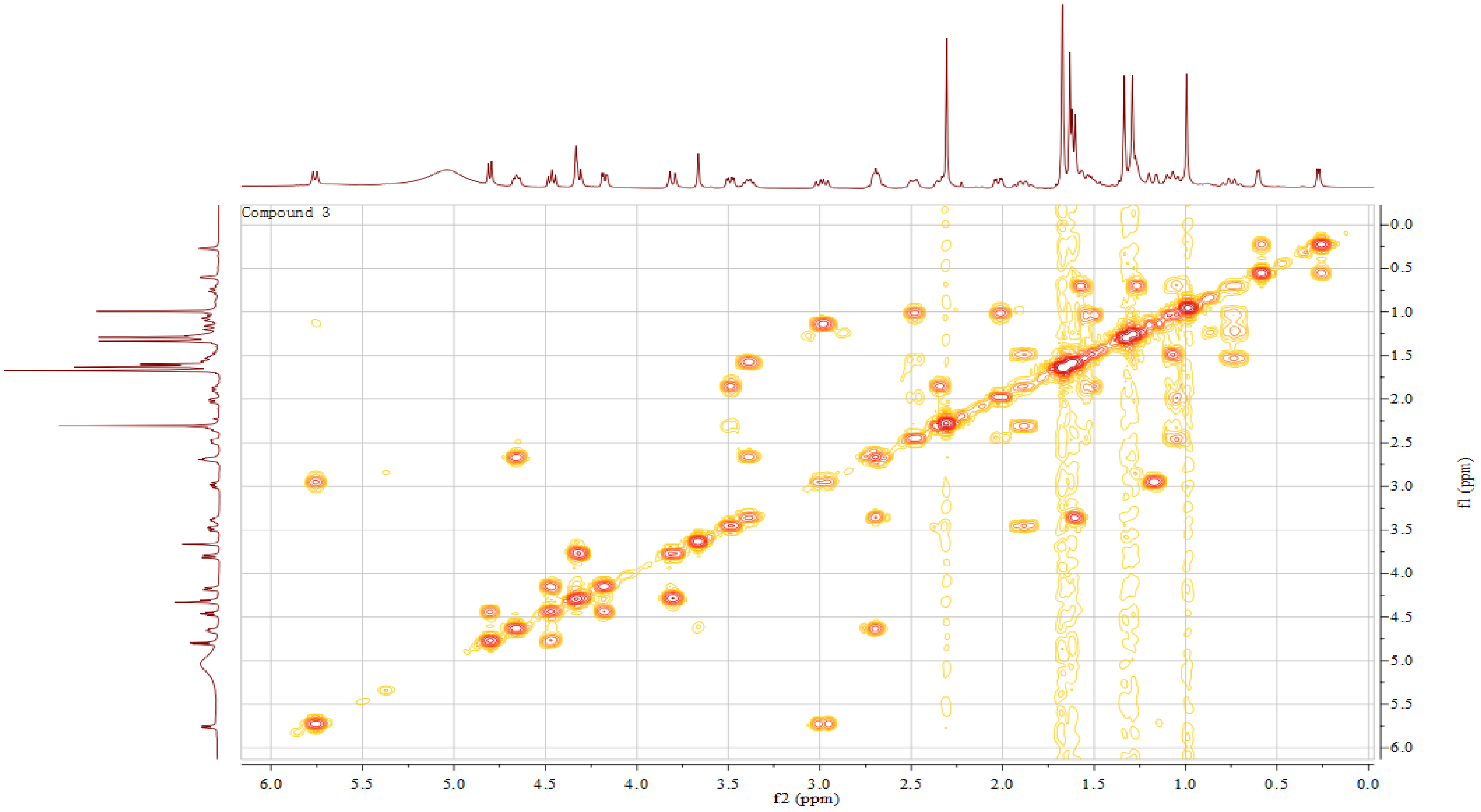

Figure S22. ROESY Spectrum of **3** in Pyridine-d_5

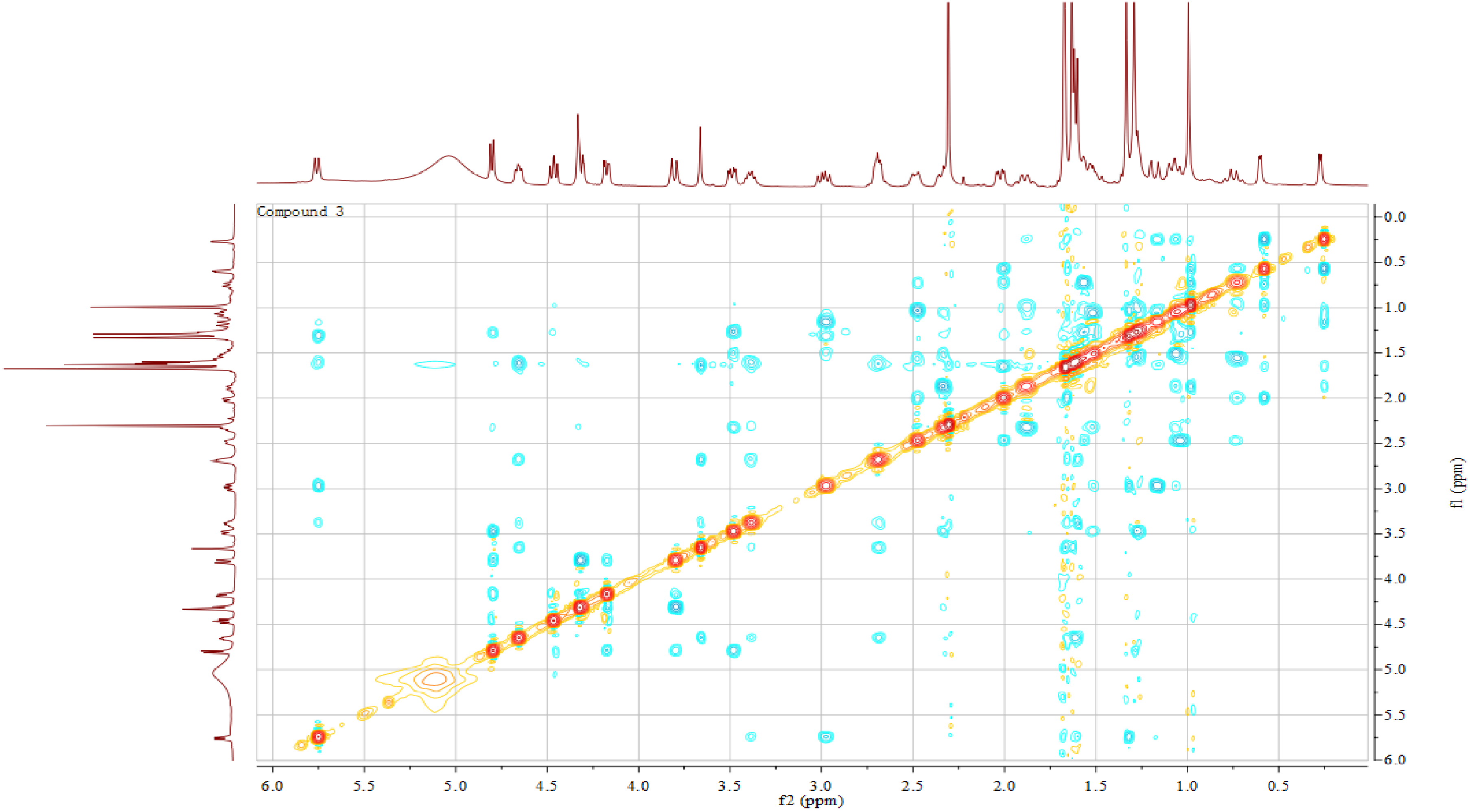

Figure S23. HRESIMS of **3**

Qualitative Analysis Report

**Data Filename**		**Sample Name**	
**Sample Type**	Sample	**Position**	
**Instrument Name**	Agilent G6230 TOF MS	**User Name**	
**Acq Method**	ESI.m	**Acquired Time**	
**IRM Calibration Status**	Success	**DA Method**	ESI.m
**Comment**			

Sample Group **Info.**
Acquisition SW 6200 series TOF/6500 series
Version Q-TOF B.05.01 (B5125.2)

User Spectra

Fragmentor Voltage	Collision Energy	Ionization Mode
200	0	ESI

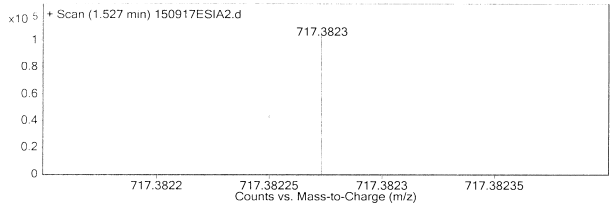

Peak List

m/z	z	Abund	Formula	Ion
153.0541		7558.66		
717.3823	1	105559.87	C37 H58 Na O12	M+
718.3854	1	35515.02	C37 H58 Na O12	M+
1064.5778	2	8940.06		
1065.0792	2	10880.18		
1107.6575	1	10876.28		
1379.8281	1	7292.69		
1411.774	1	55602.59		
1412.777	1	45462.13		
1413.7799	1	15454.35		

Formula Calculator Element Limits

Element	Min	Max
C	0	200
H	0	400
O	0	15
Na	1	1

Formula Calculator Results

Formula	CalculatedMass	CalculatedMz	Mz	Diff. (mDa)	Diff. (ppm)	DBE
C37 H58 Na O12	717.3826	717.3820	717.3823	-0.1	-0.2	8.5000

--- End Of Report ---

Figure S24. IR Spectrum of **3**

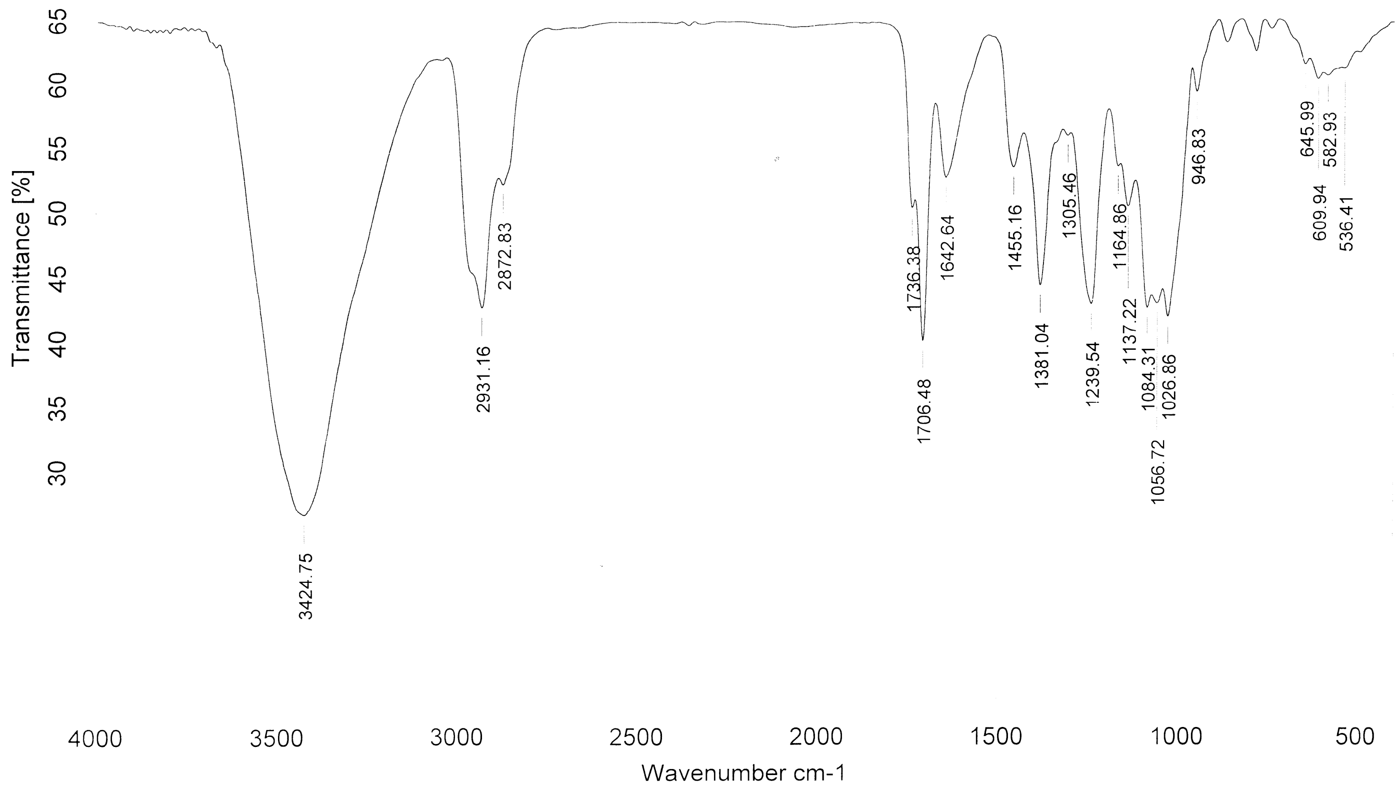

Figure S25. ^{1}H NMR Spectrum of **4** in Pyridine-d_5

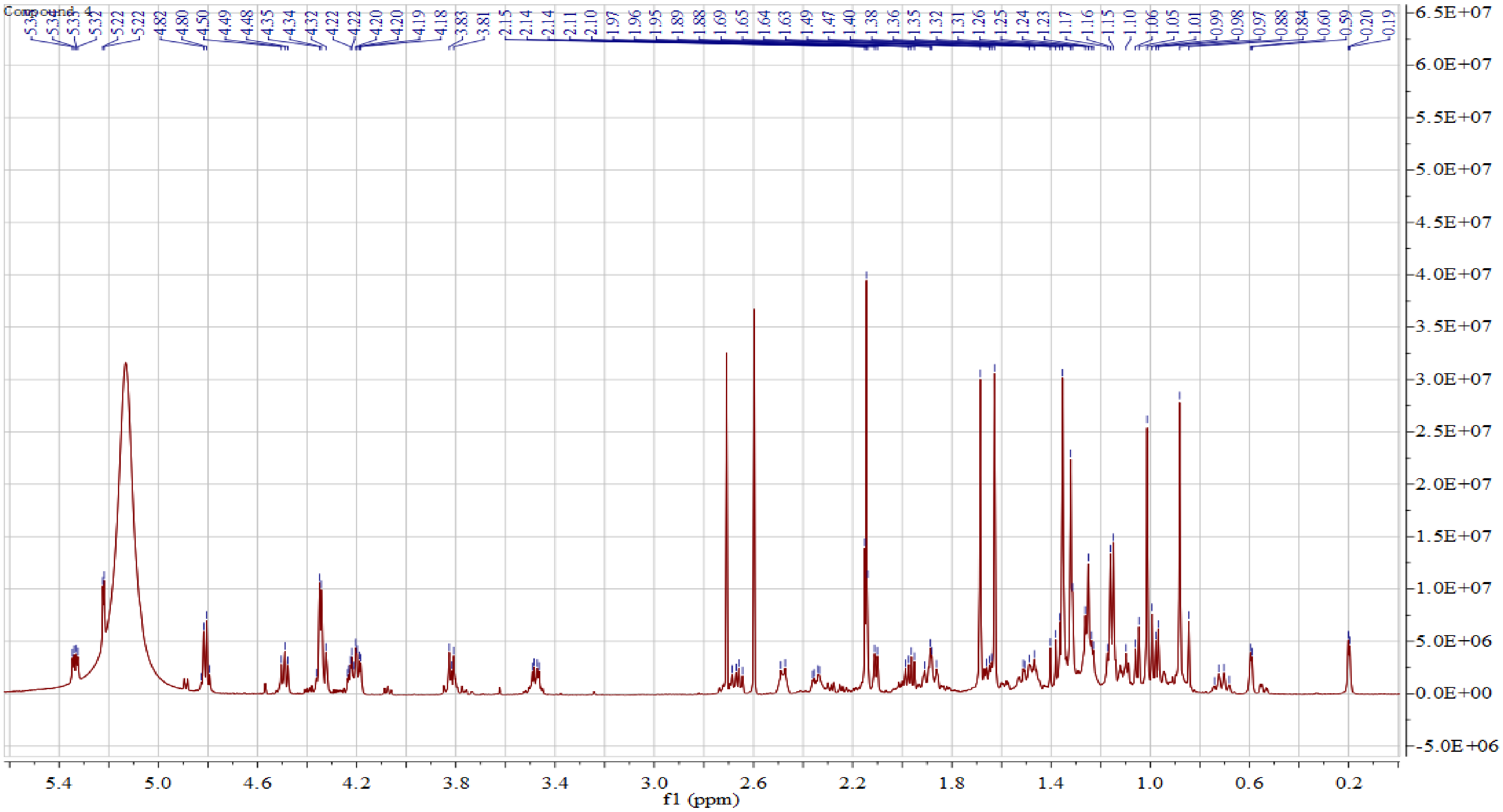

Figure S26. ^{13}C NMR Spectrum of **4** in Pyridine-d_5

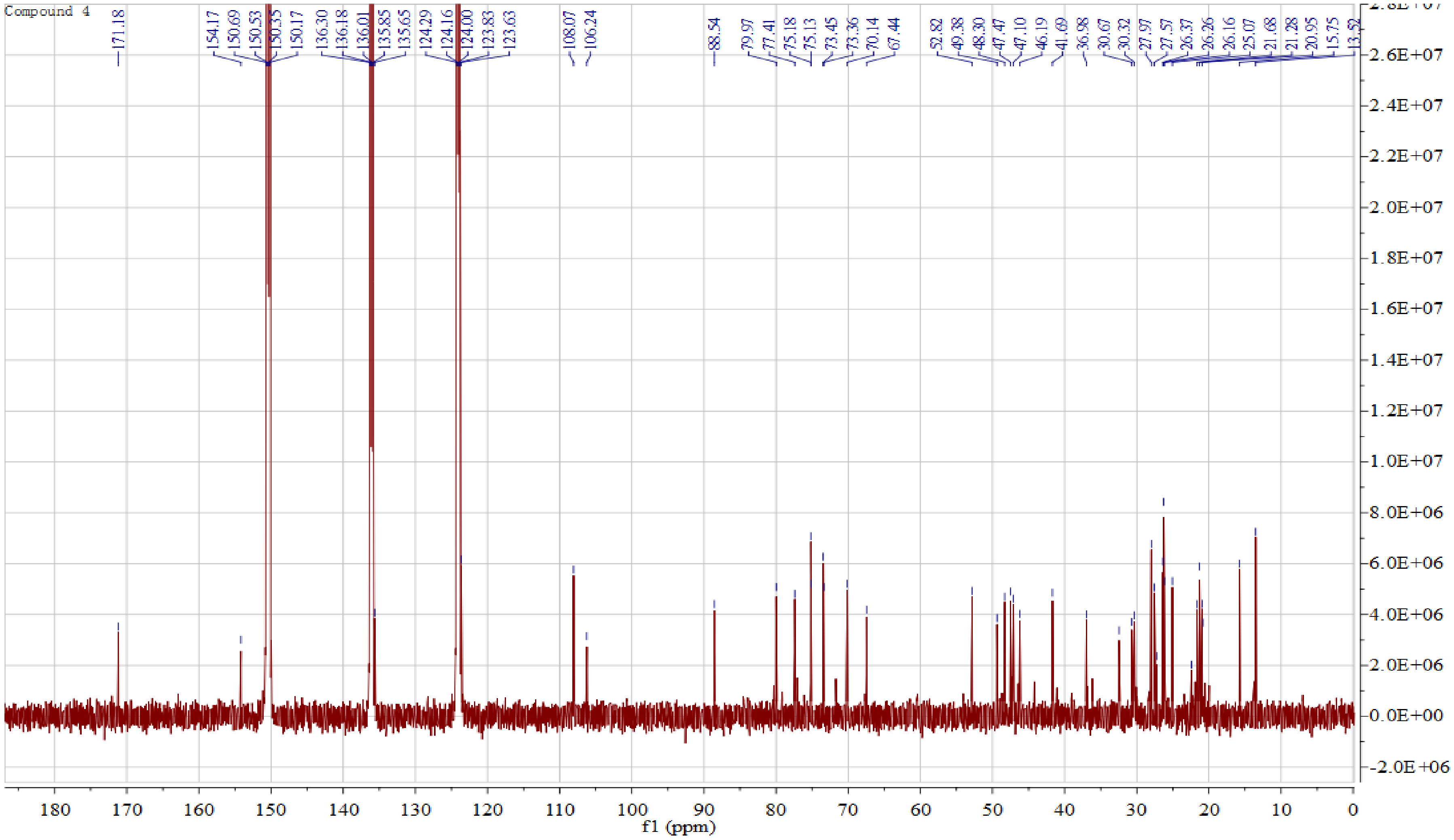

Figure S27. HSQC Spectrum of **4** in Pyridine-d_5

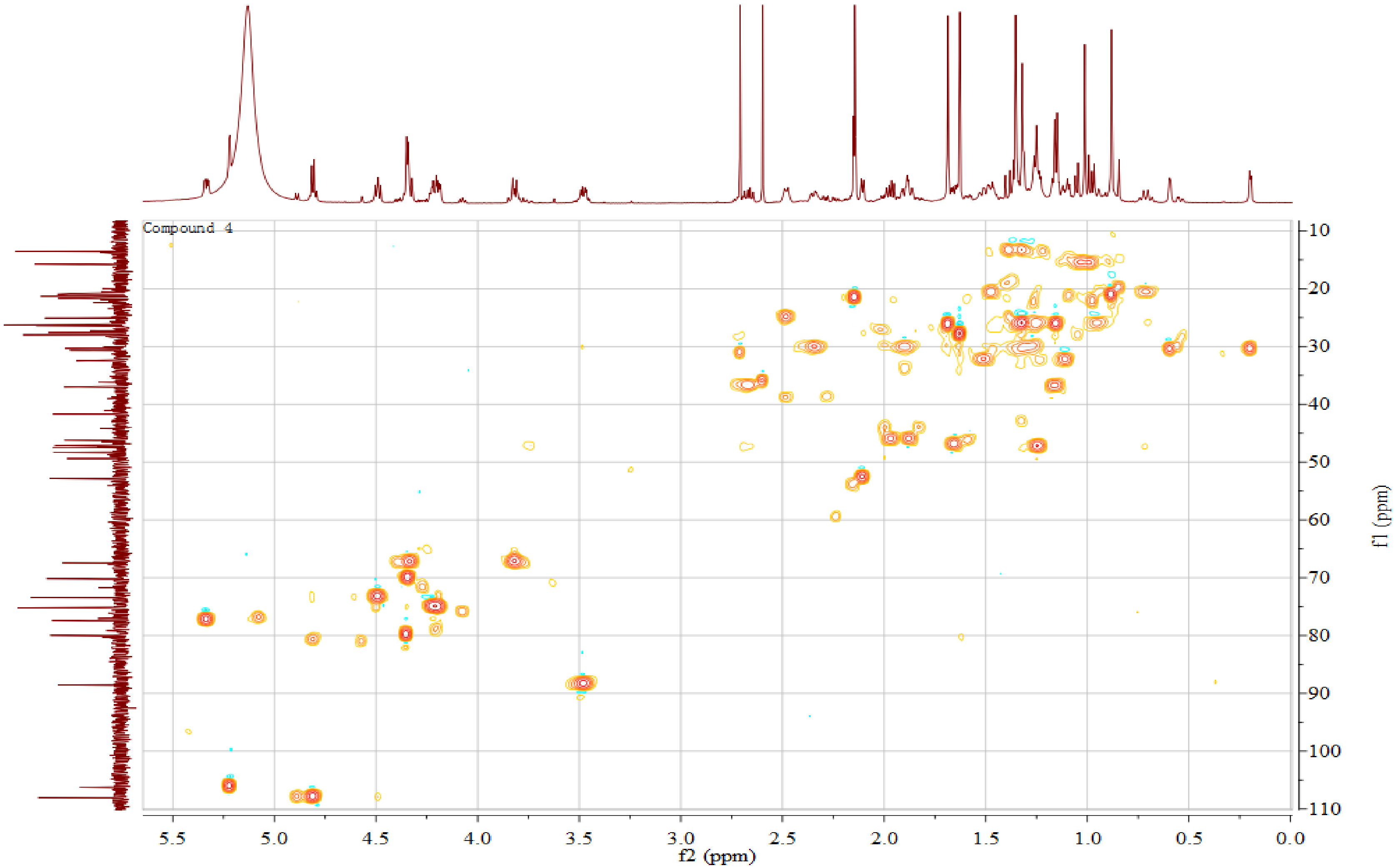

Figure S28. HMBC Spectrum of **4** in Pyridine-d_5

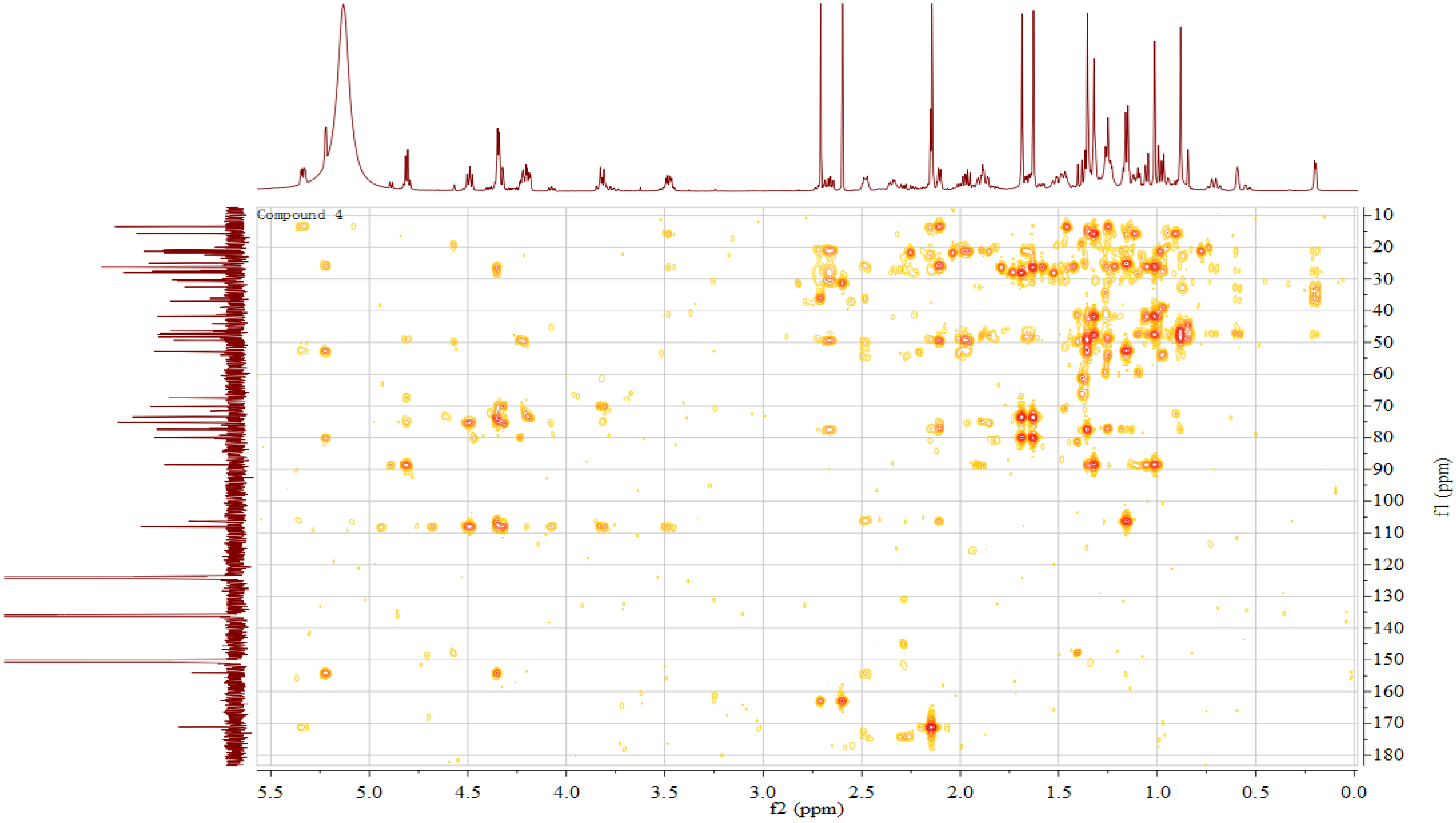

Figure S29. ¹H-¹H COSY Spectrum of **4** in Pyridine-d_5

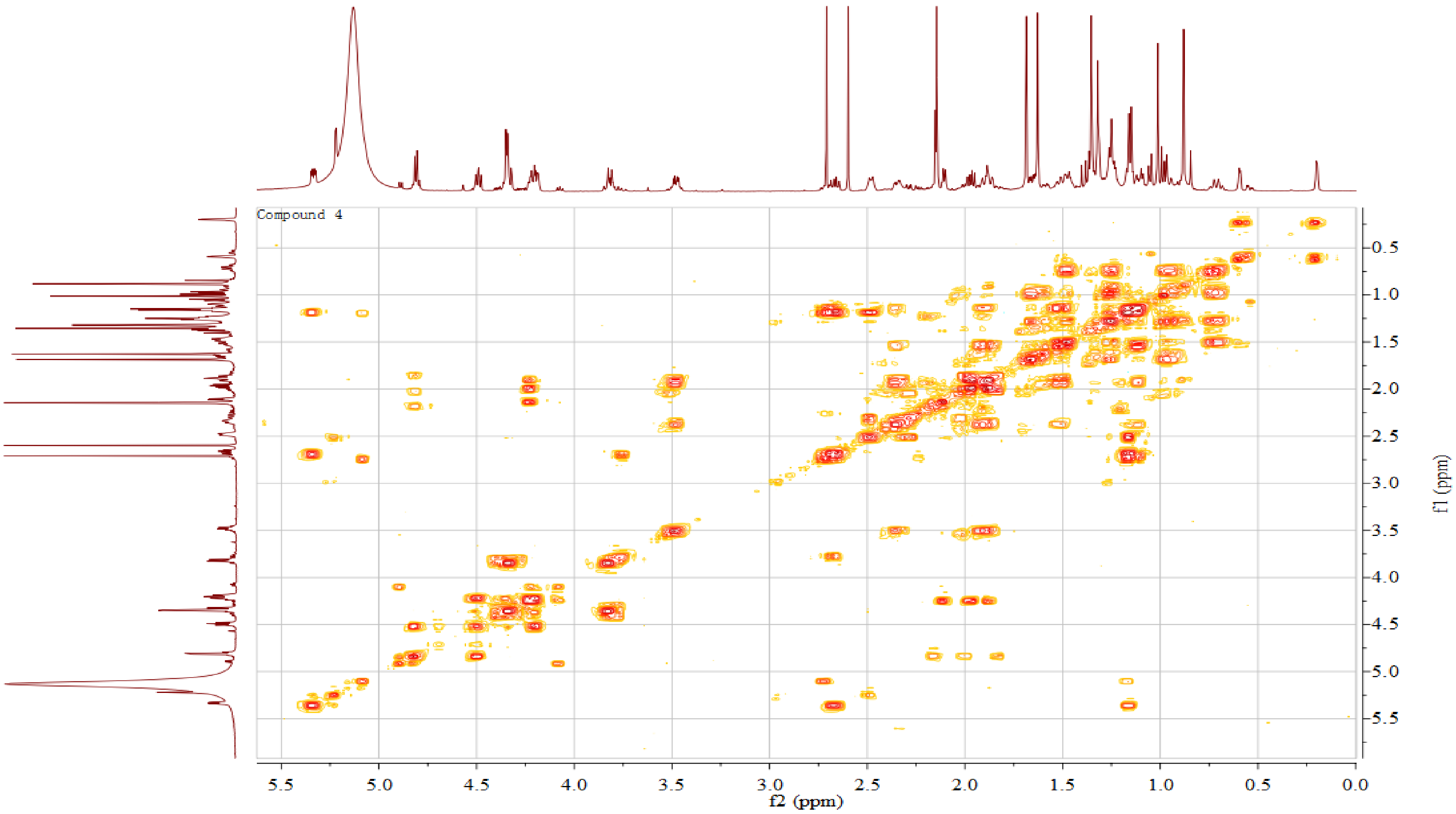

Figure S30. ROESY Spectrum of **4** in Pyridine-d_5

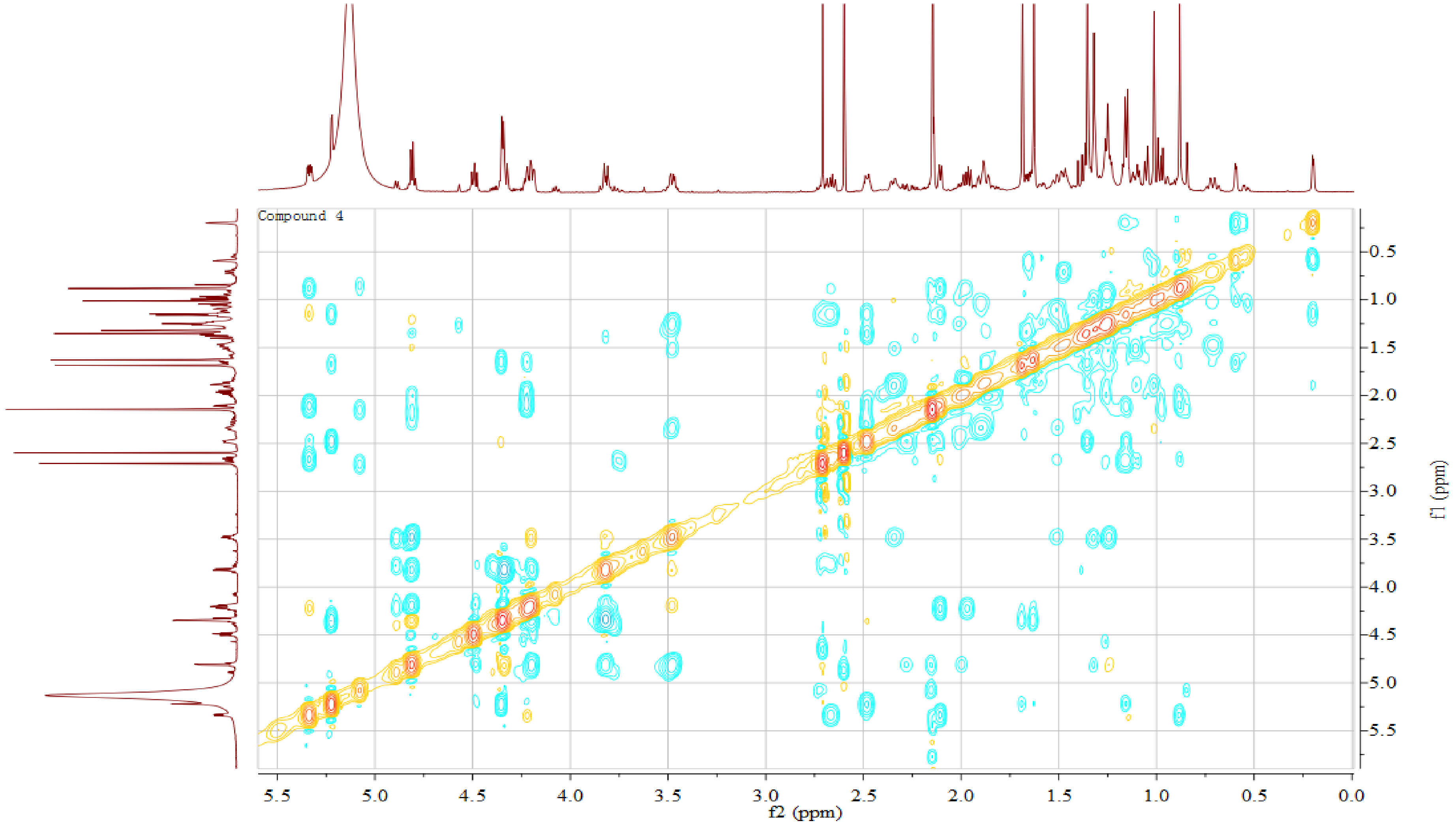

Figure S31. HRESIMS of **4**

Elemental Composition Report

Page 1

Single Mass Analysis

Tolerance = 10.0 PPM / DBE: min = -10.0, max = 120.0
Selected filters: None

Monoisotopic Mass, Odd and Even Electron Ions
25 formula(e) evaluated with 1 results within limits (up to 51 closest results for each mass)
Elements Used:
C: 0-200 H: 0-400 O: 9-11

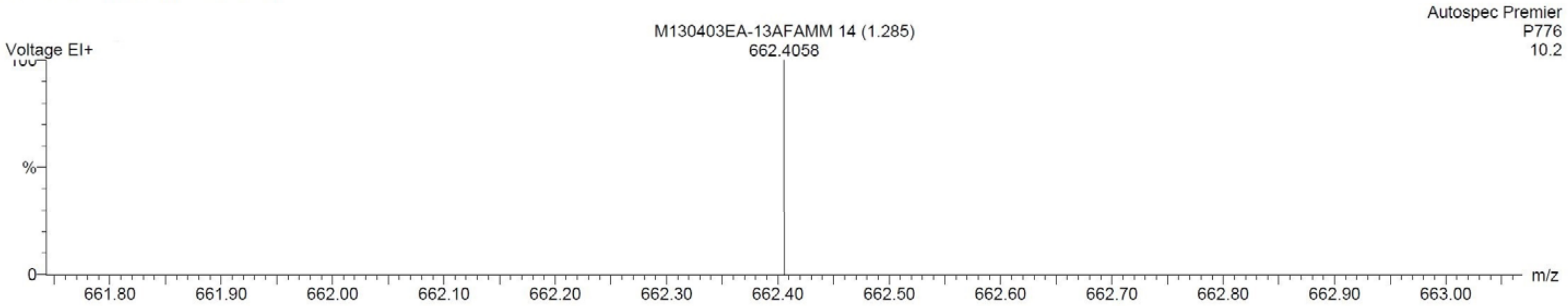

Minimum:				-10.0		
Maximum:		200.0	10.0	120.0		
Mass	Calc. Mass	mDa	PPM	DBE	i-FIT	Formula
662.4058	662.4030	2.8	4.2	9.0	5546027.0	C37 H58 O10

Figure S32. IR Spectrum of **4**

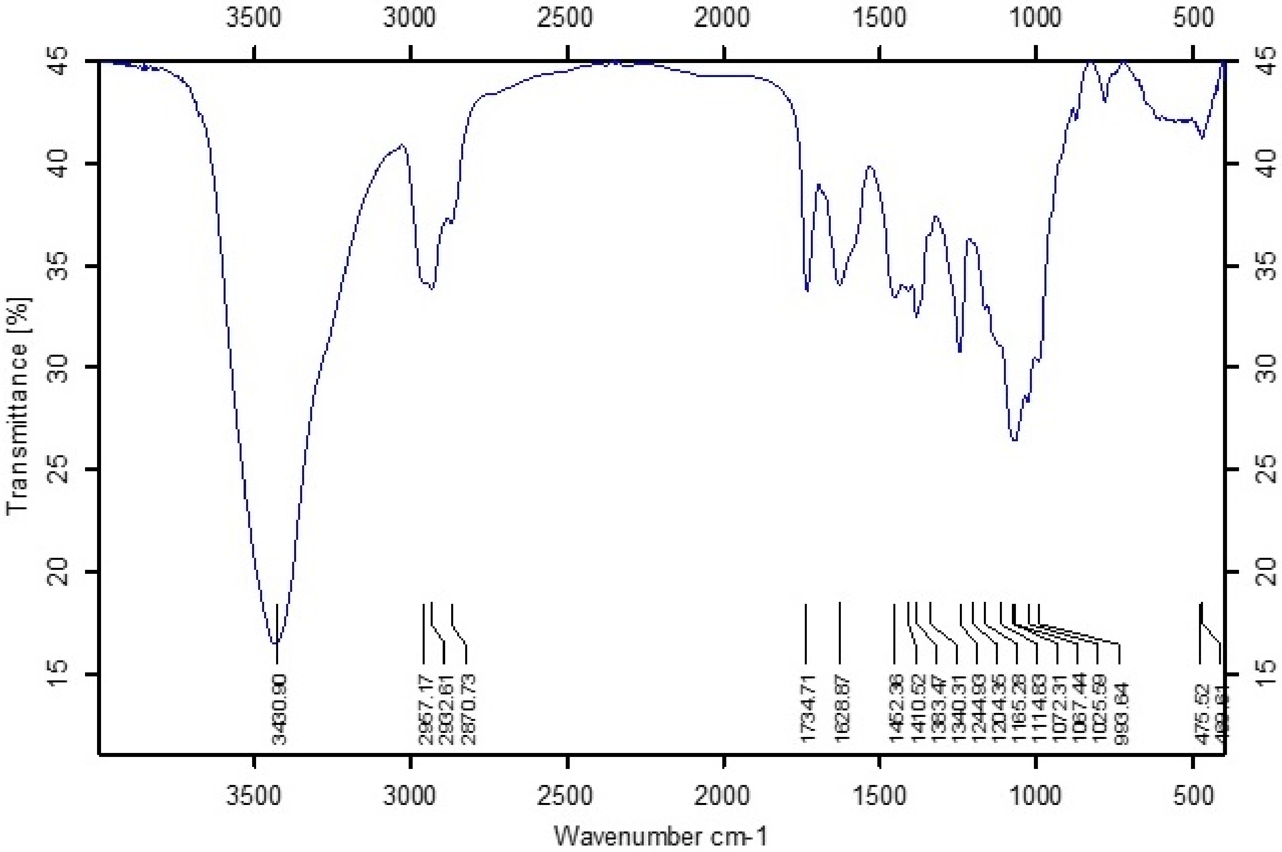

Figure S33. ^{1}H NMR Spectrum of **5** in Pyridine-d_5

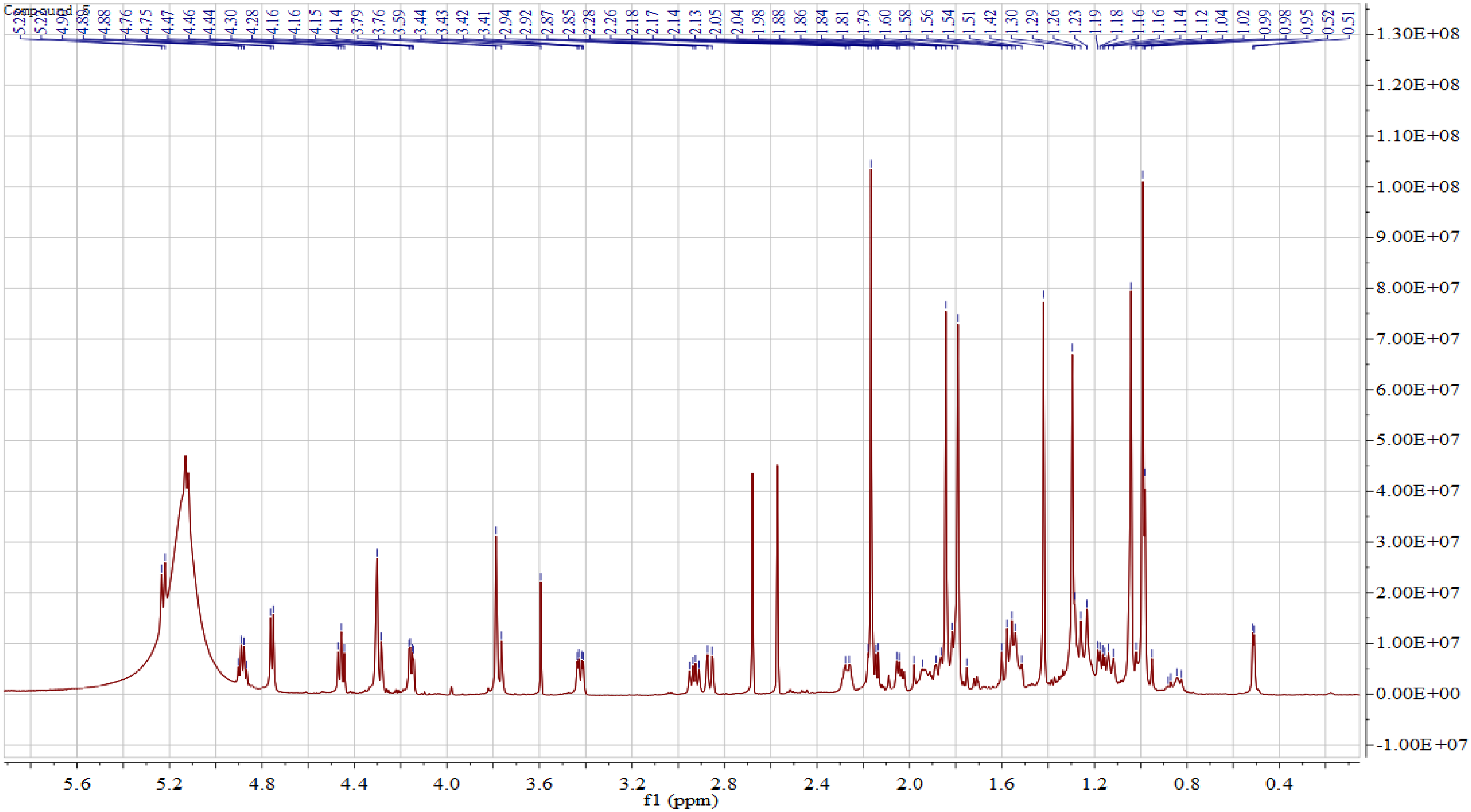

Figure S34. ^{13}C NMR Spectrum of **5** in Pyridine-d_5

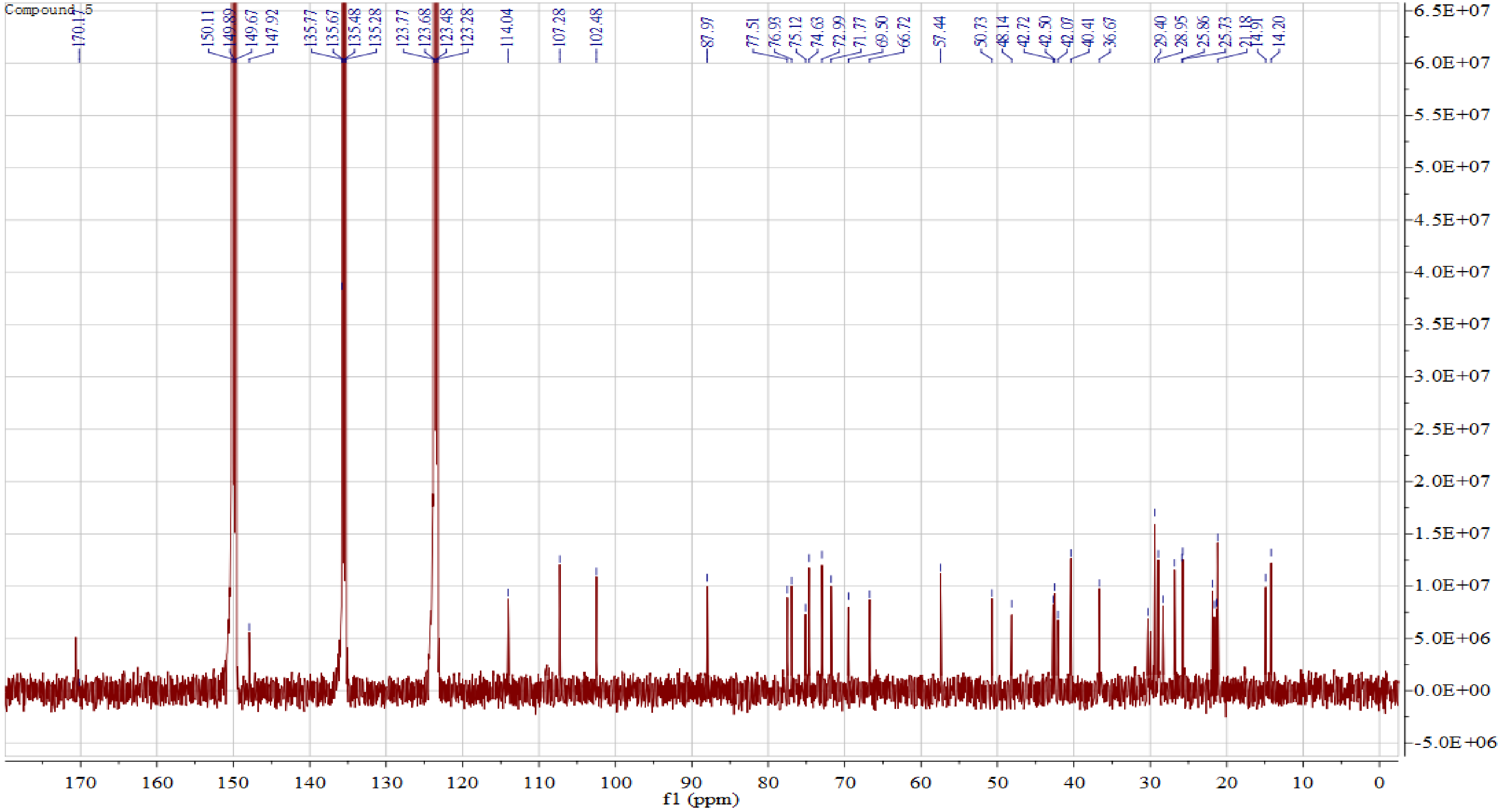

Figure S35. HSQC Spectrum of **5** in Pyridine-d_5

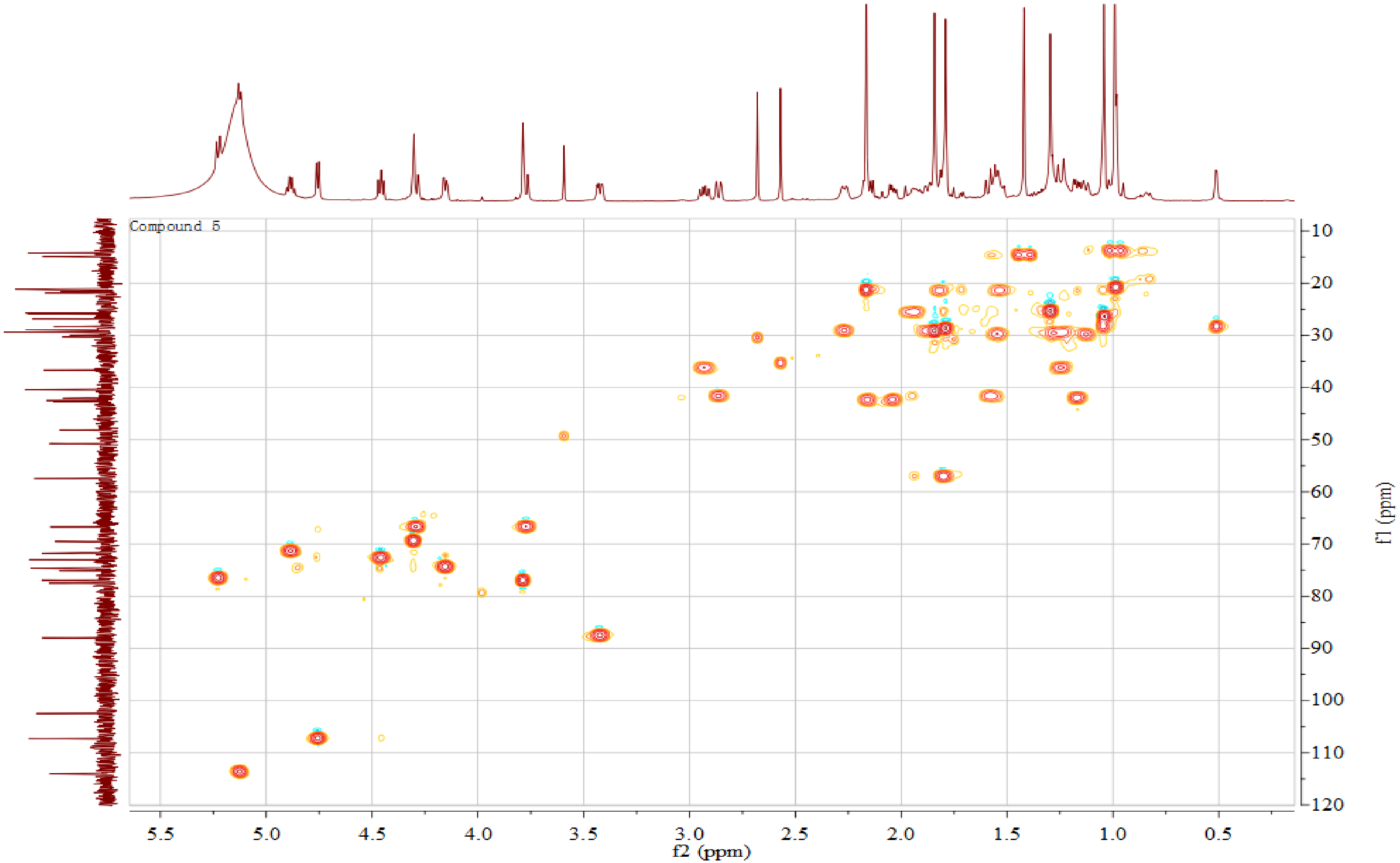

Figure S36. HMBC Spectrum of **5** in Pyridine-d_5

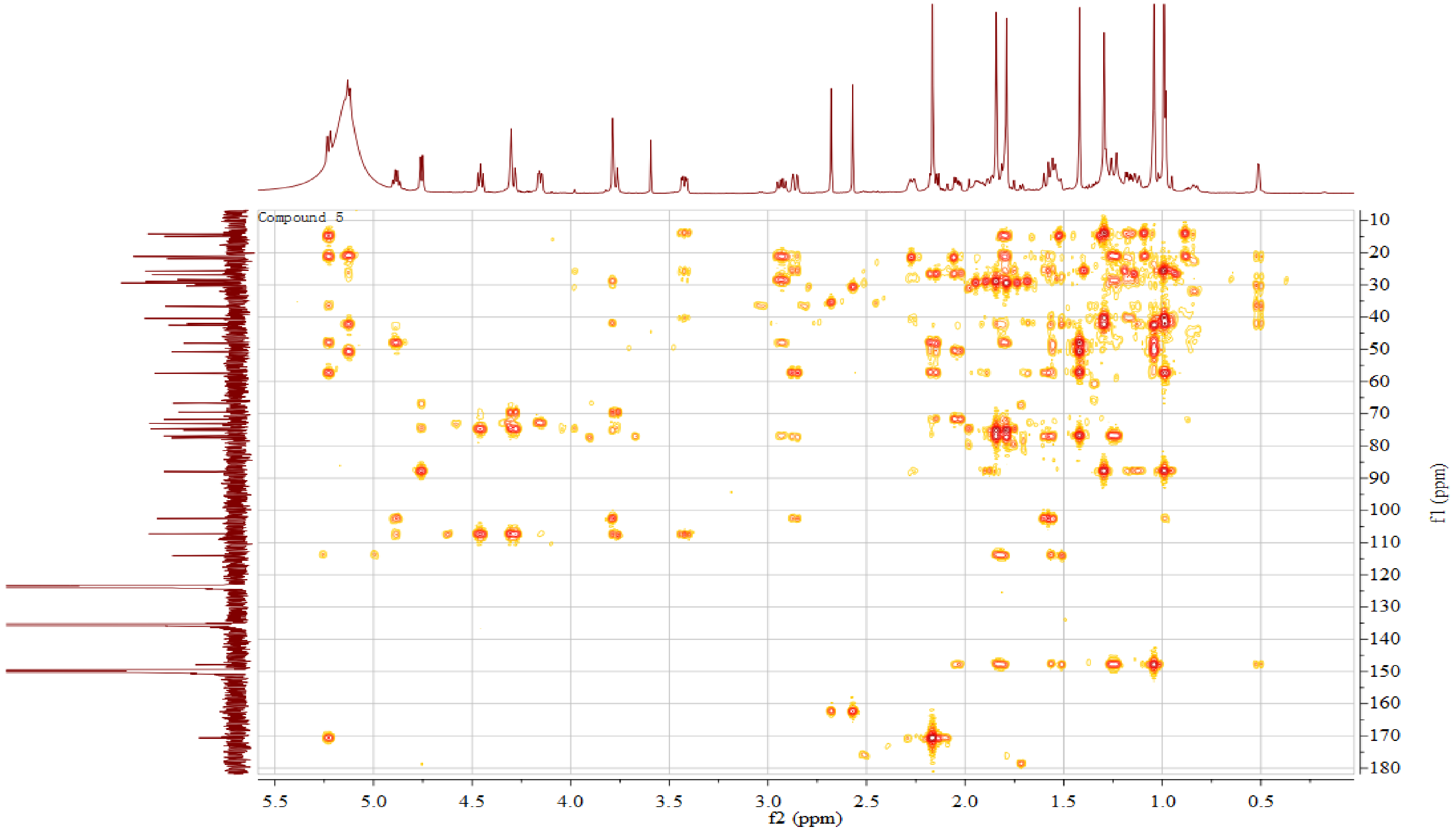

Figure S37. ^{1}H-^{1}H COSY Spectrum of **5** in Pyridine-d_5

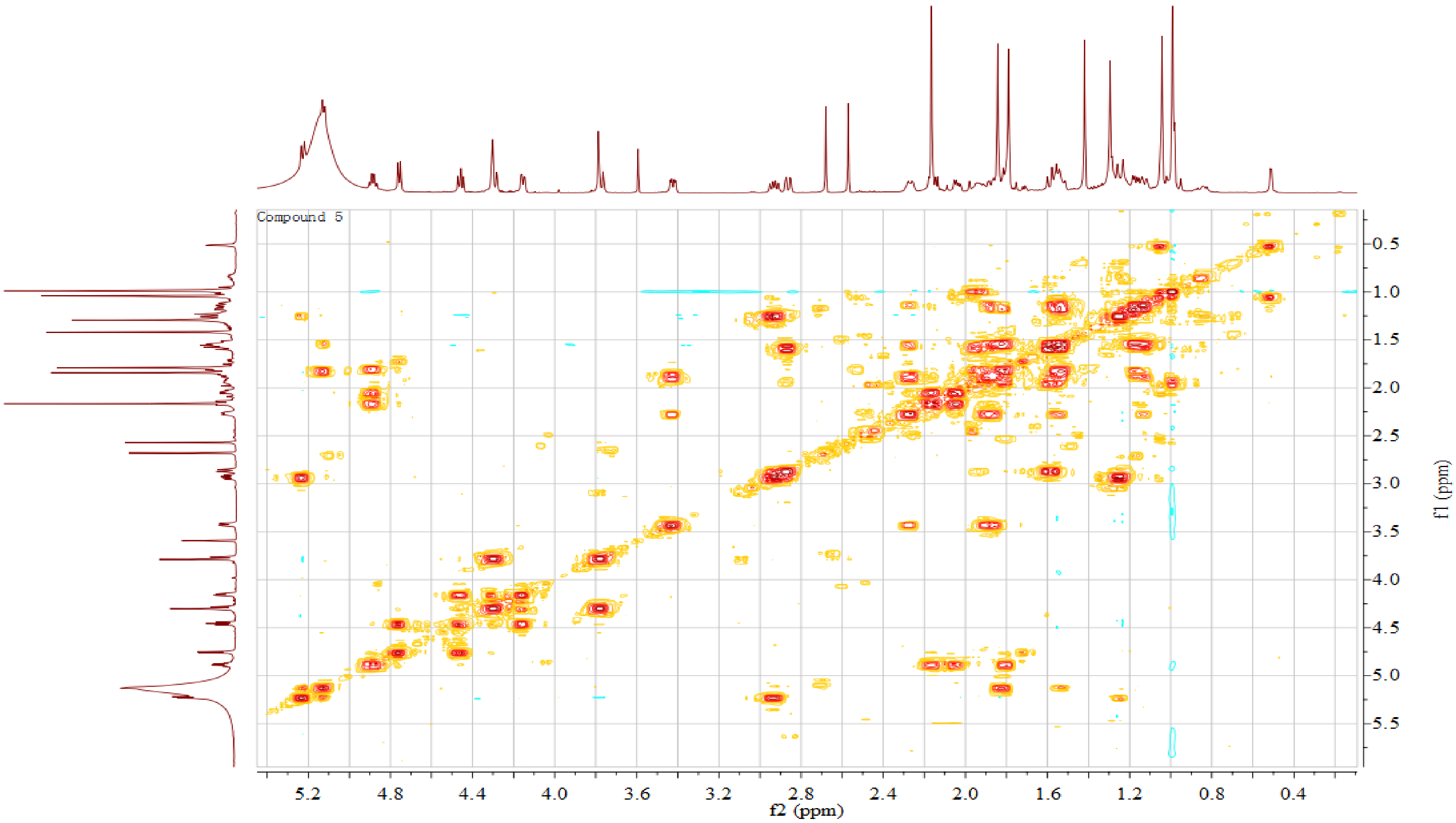

Figure S38. ROESY Spectrum of **5** in Pyridine-d_5

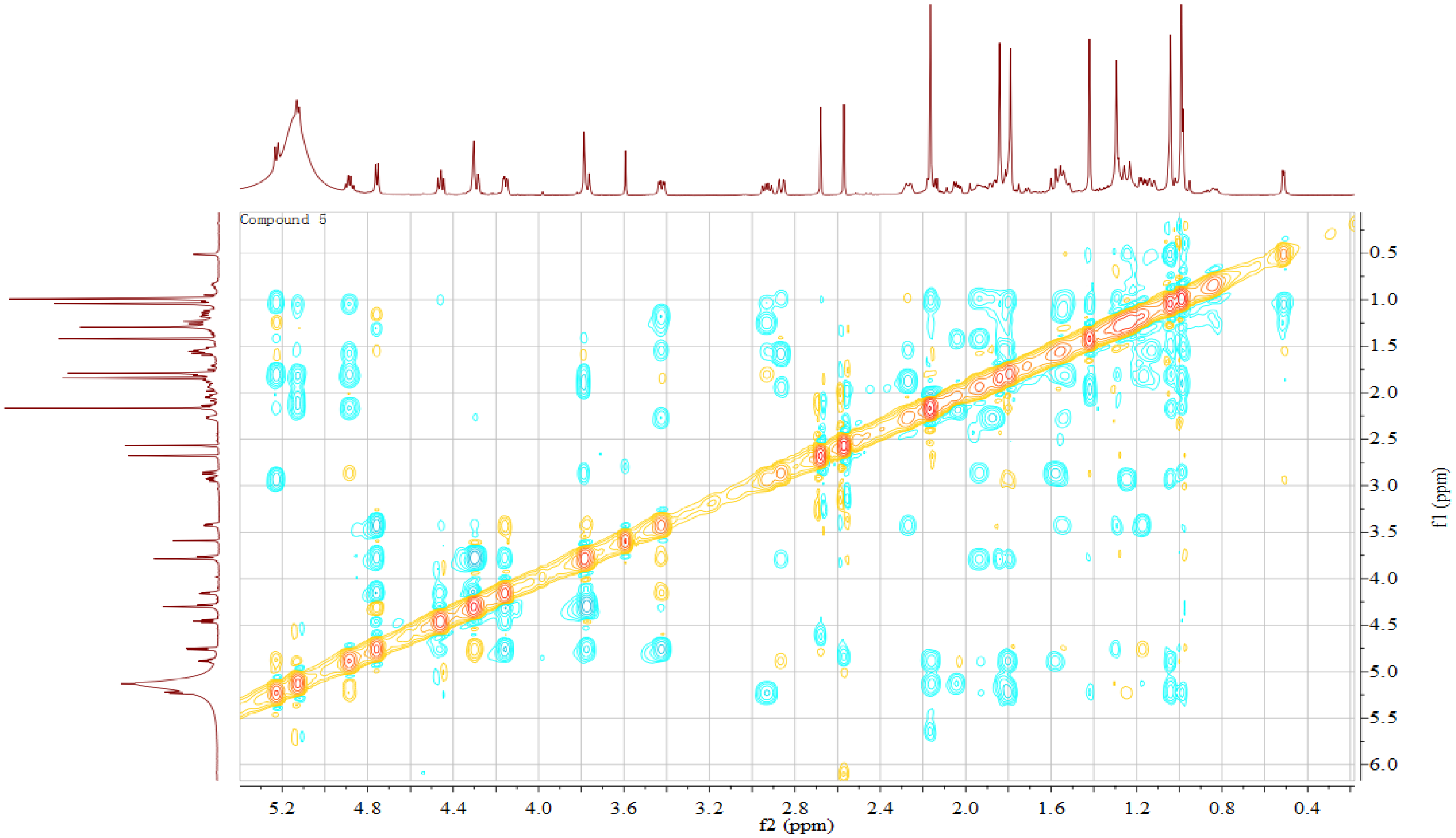

Figure S39. HREIMS of **5**

Elemental Composition Report

Page 1

Single Mass Analysis

Tolerance = 10.0 PPM / DBE: min = -10.0, max = 120.0
Selected filters: None

Monoisotopic Mass, Odd and Even Electron Ions
25 formula(e) evaluated with 1 results within limits (up to 51 closest results for each mass)
Elements Used:
C: 0-200 H: 0-400 O: 10-12

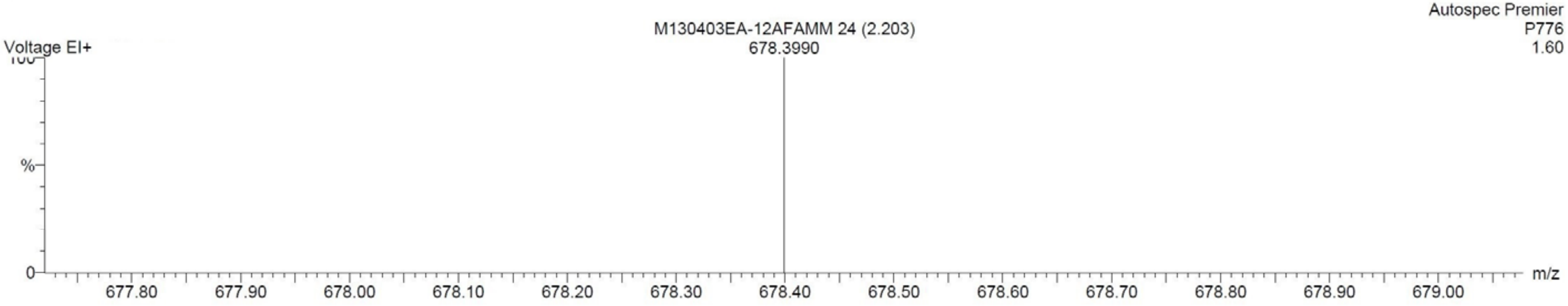

Minimum:				-10.0		
Maximum:		200.0	10.0	120.0		
Mass	Calc. Mass	mDa	PPM	DBE	i-FIT	Formula
678.3990	678.3979	1.1	1.6	9.0	5546025.5	C37 H58 O11

Figure S40. IR Spectrum of **5**

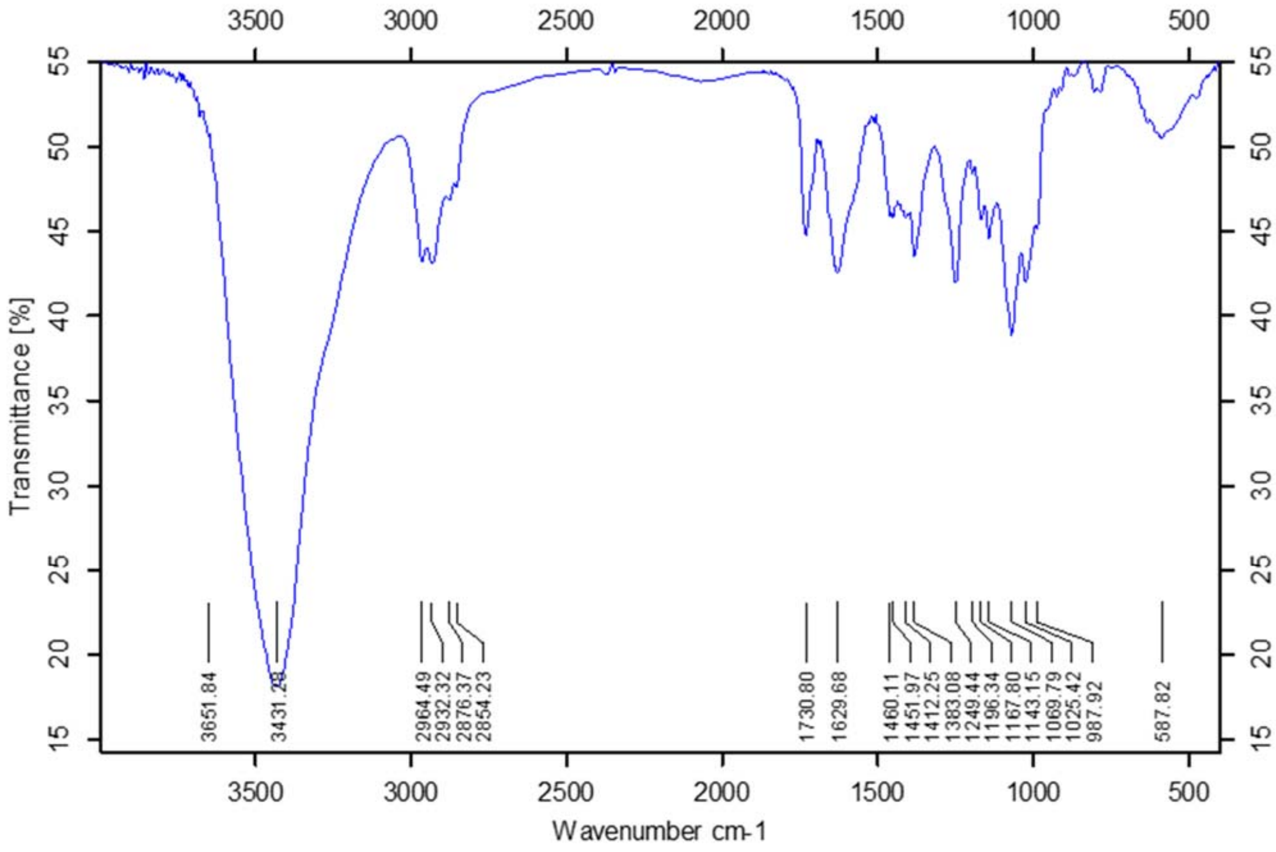

Figure S41. ^{1}H NMR Spectrum of **6** in Pyridine-d_5

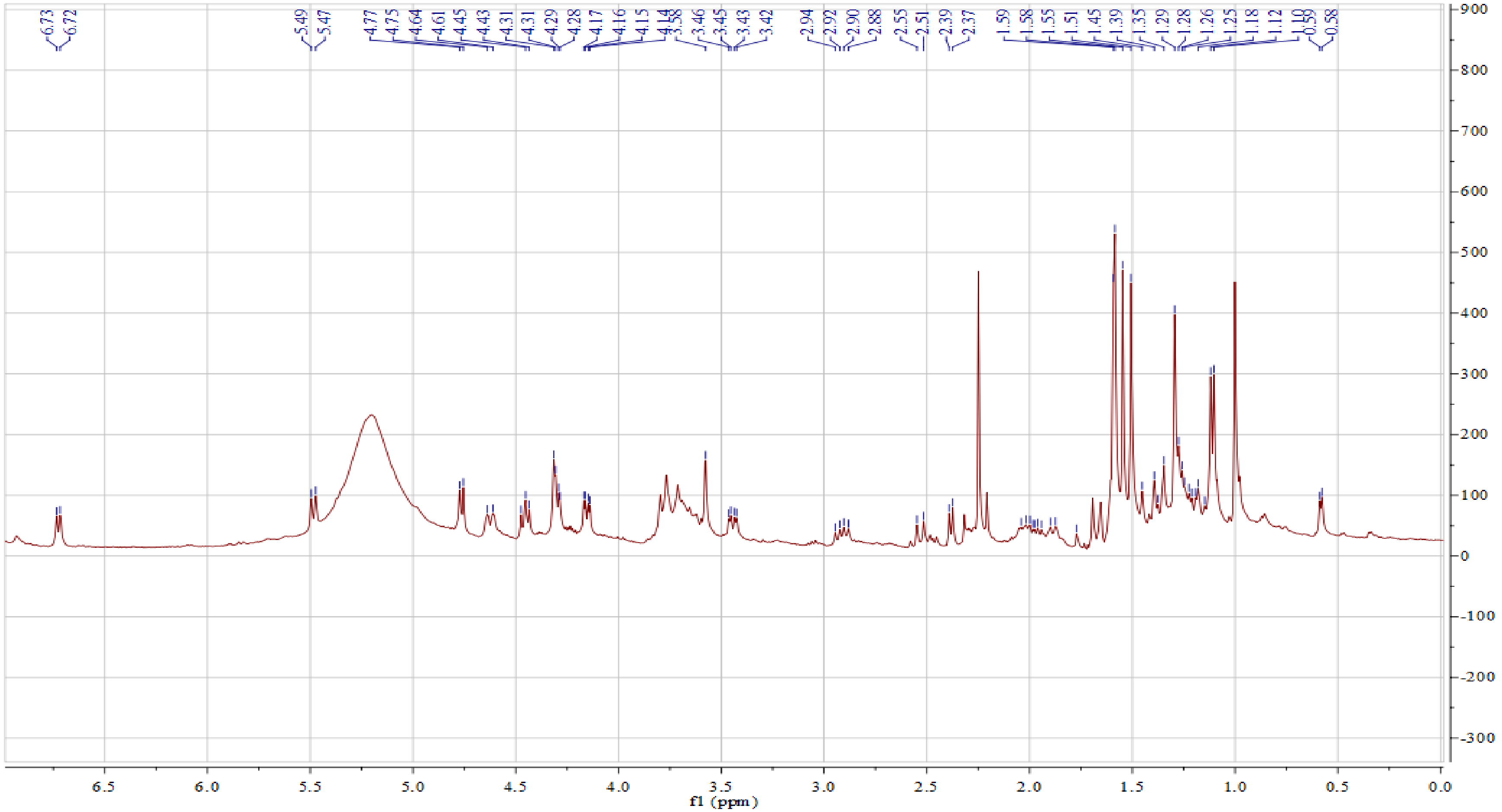

Figure S42. ^{13}C NMR Spectrum of **6** in Pyridine-d_5

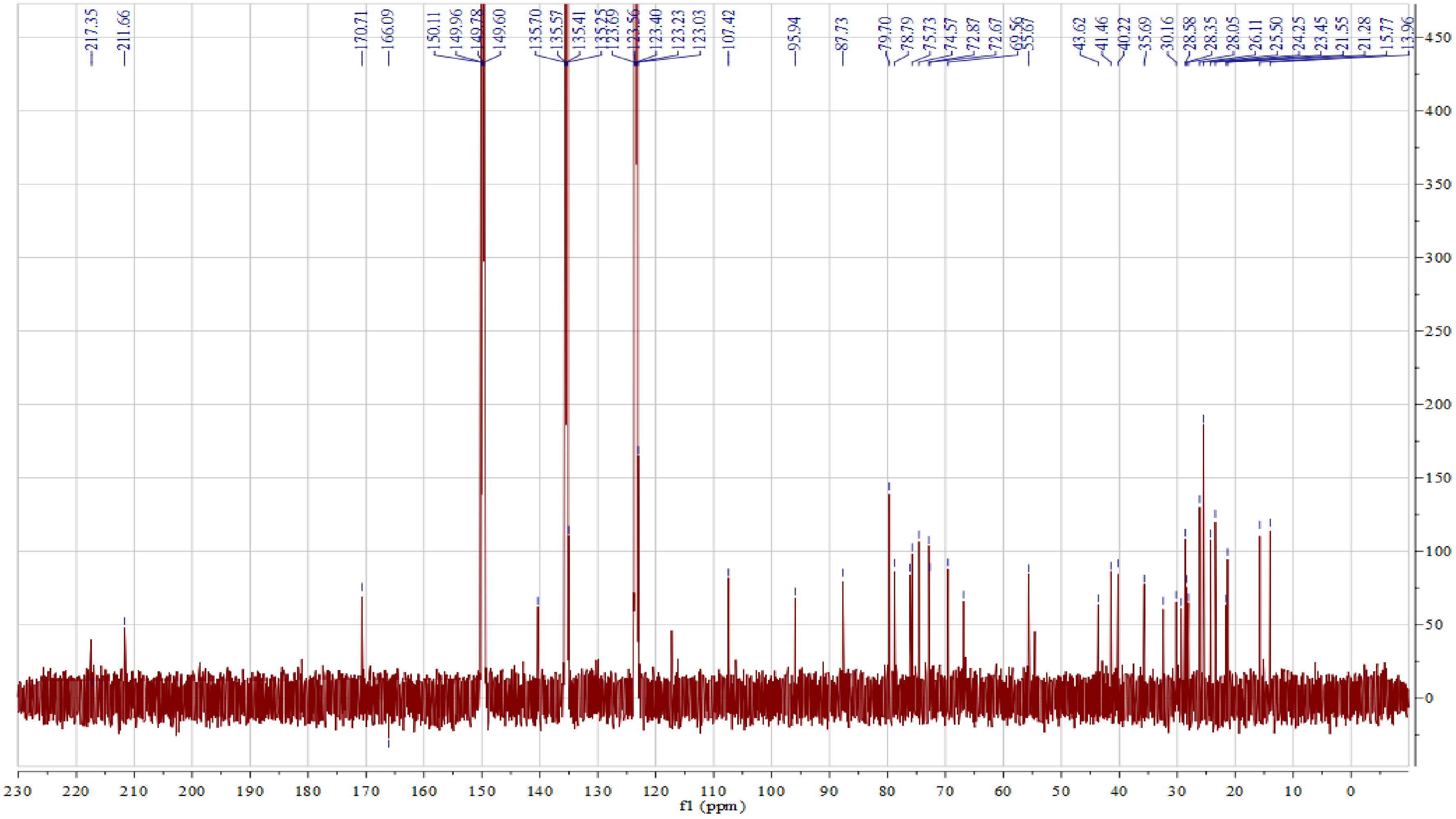

Figure S43. HSQC Spectrum of **6** in Pyridine-d_5

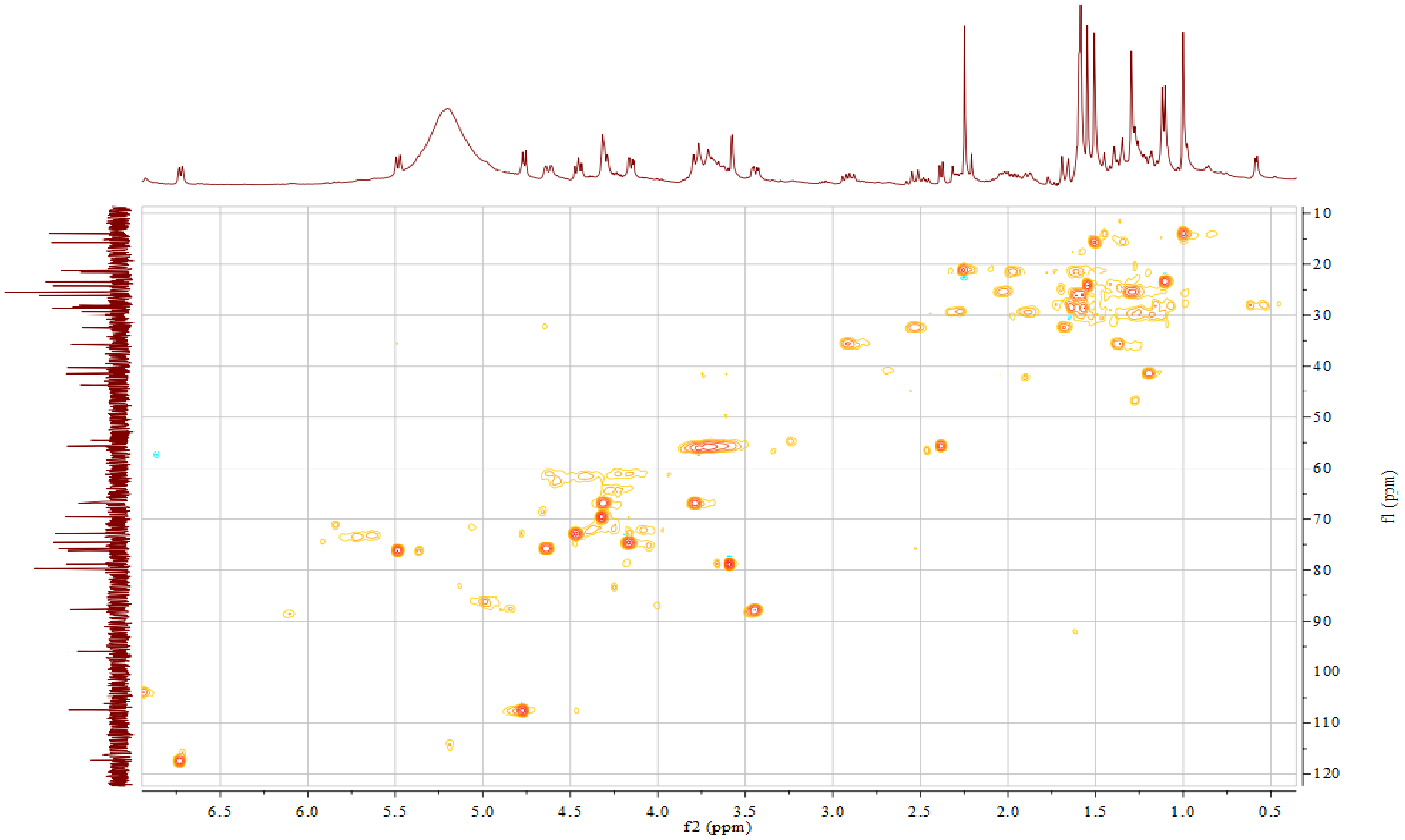

Figure S44. HMBC Spectrum of **6** in Pyridine-d_5

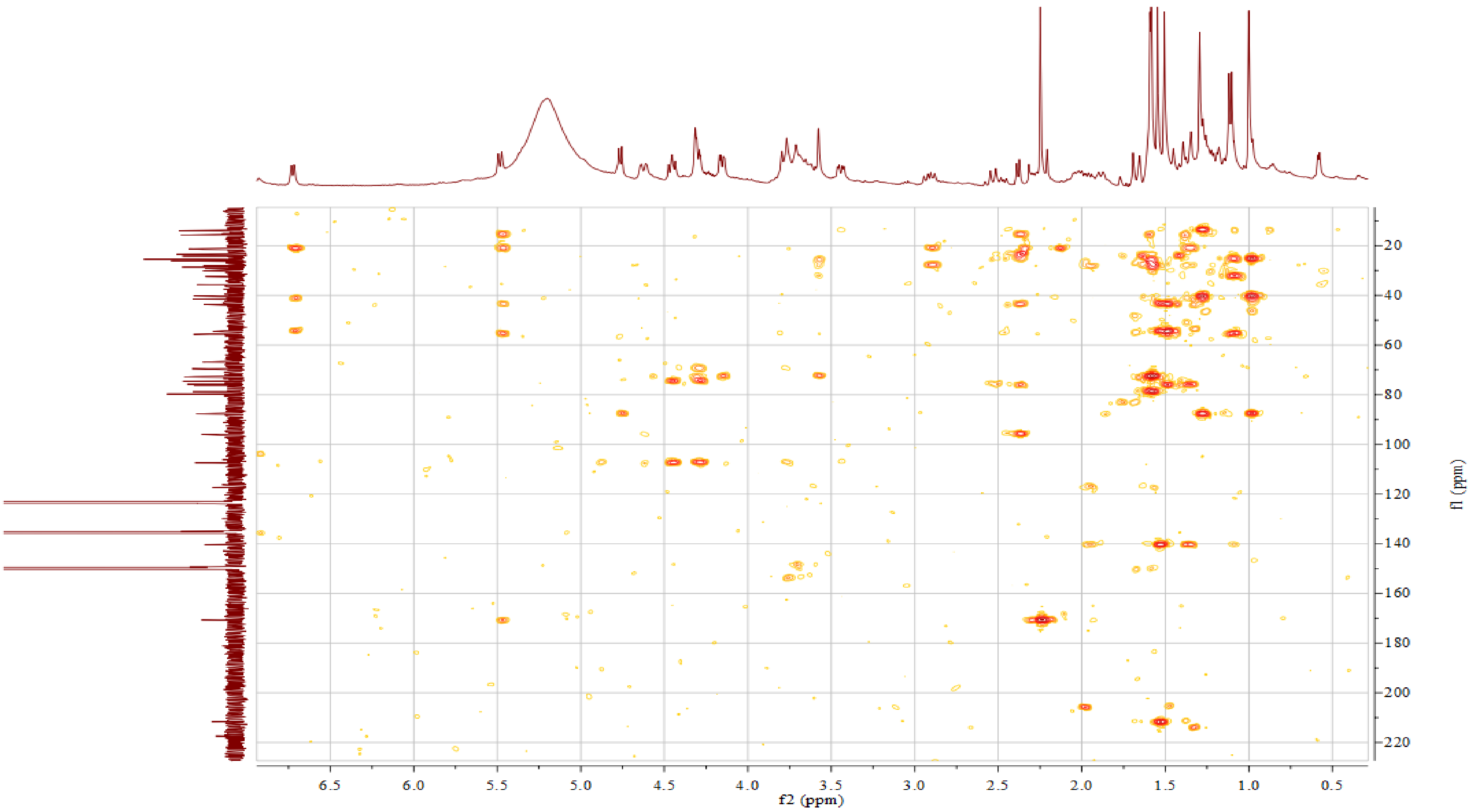

Figure S45. ^{1}H-^{1}H COSY Spectrum of **6** in Pyridine-d_5

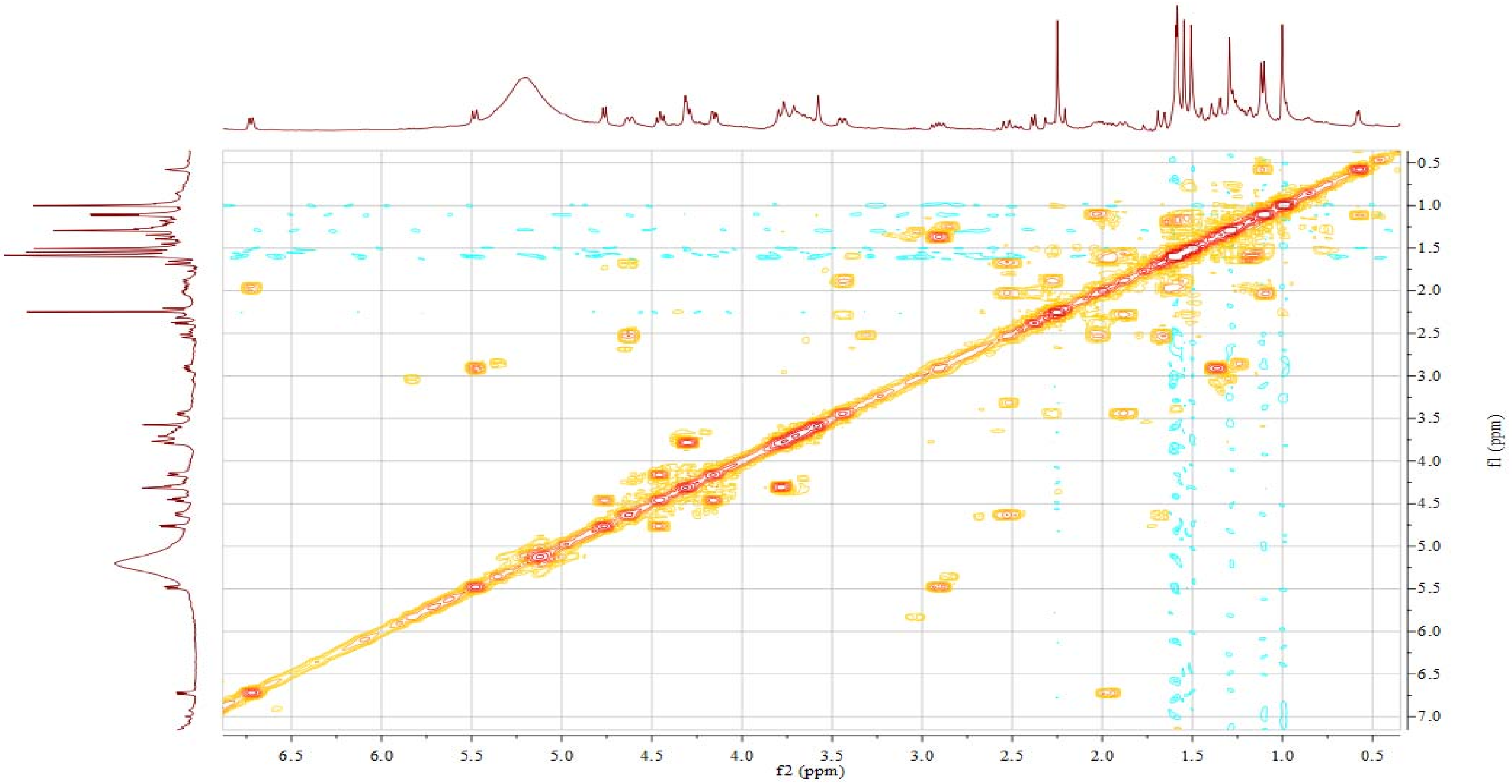

Figure S46. ROESY Spectrum of **6** in Pyridine-d_5

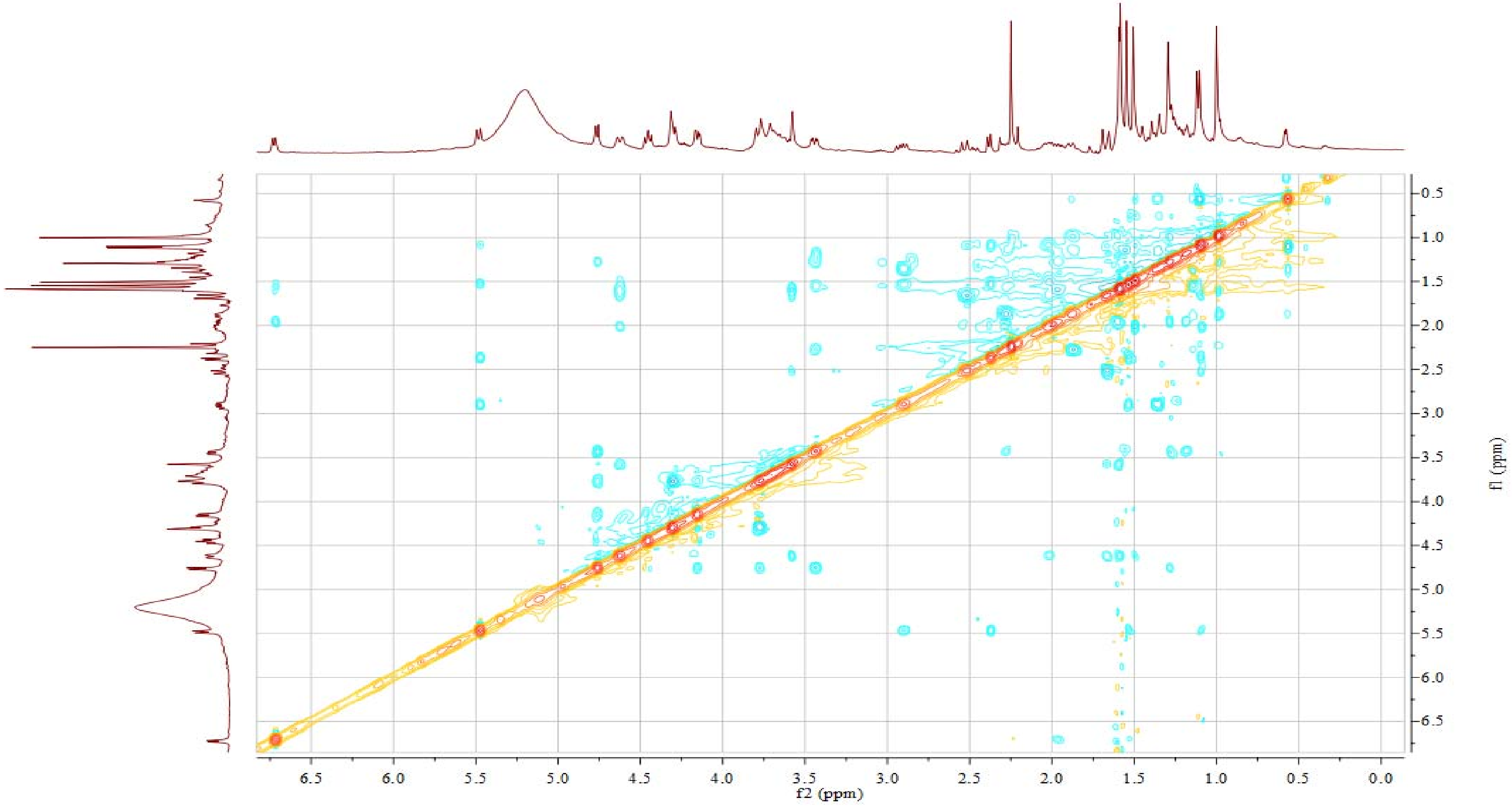

Figure S47. HREIMS of **6**

Qualitative Analysis Report

**Data Filename**		**Sample Name**	
**Sample Type**	Sample	**Position**	
**Instrument Name**	Agilent G6230 TOF MS	**User Name**	
**Acq Method**	ESI.m	**Acquired Time**	
**IRM Calibration Status**	Success	**DA Method**	ESI.m
**Comment**			

Sample Group **Info.**
Acquisition SW Version 6200 series TOF/6500 series Q-TOF B.05.01 (B5125.2)

User Spectra

Fragmentor Voltage	Collision Energy	Ionization Mode
250	0	ESI

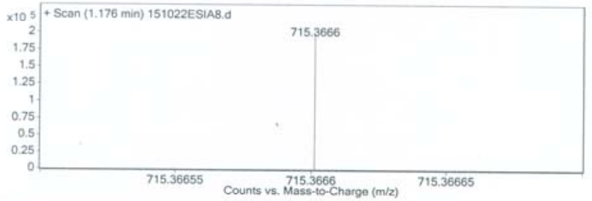

Peak List

m/z	z	Abund	Formula	Ion
299.1102	1	23707.8		
413.2664	1	27078.22		
715.3666	1	199800.11	C37 H56 Na O12	M+
716.3694	1	70347.52	C37 H56 Na O12	M+
717.3769	1	30343.79	C37 H56 Na O12	M+
922.0098	1	134236.13		
923.0115	1	20421.78		
1407.7419	1	39622.61		
1408.7446	1	31648.32		
1409.7501	1	20666.53		

Formula Calculator Element Limits

Element	Min	Max
C	0	200
H	0	400
O	5	13
Na	1	1

Formula Calculator Results

Formula	CalculatedMass	CalculatedMz	Mz	Diff. (mDa)	Diff. (ppm)	DBE
C37 H56 Na O12	715.3670	715.3664	715.3666	-0.6	-0.8	9.5000

--- End Of Report ---

Figure S48. IR Spectrum of **6**

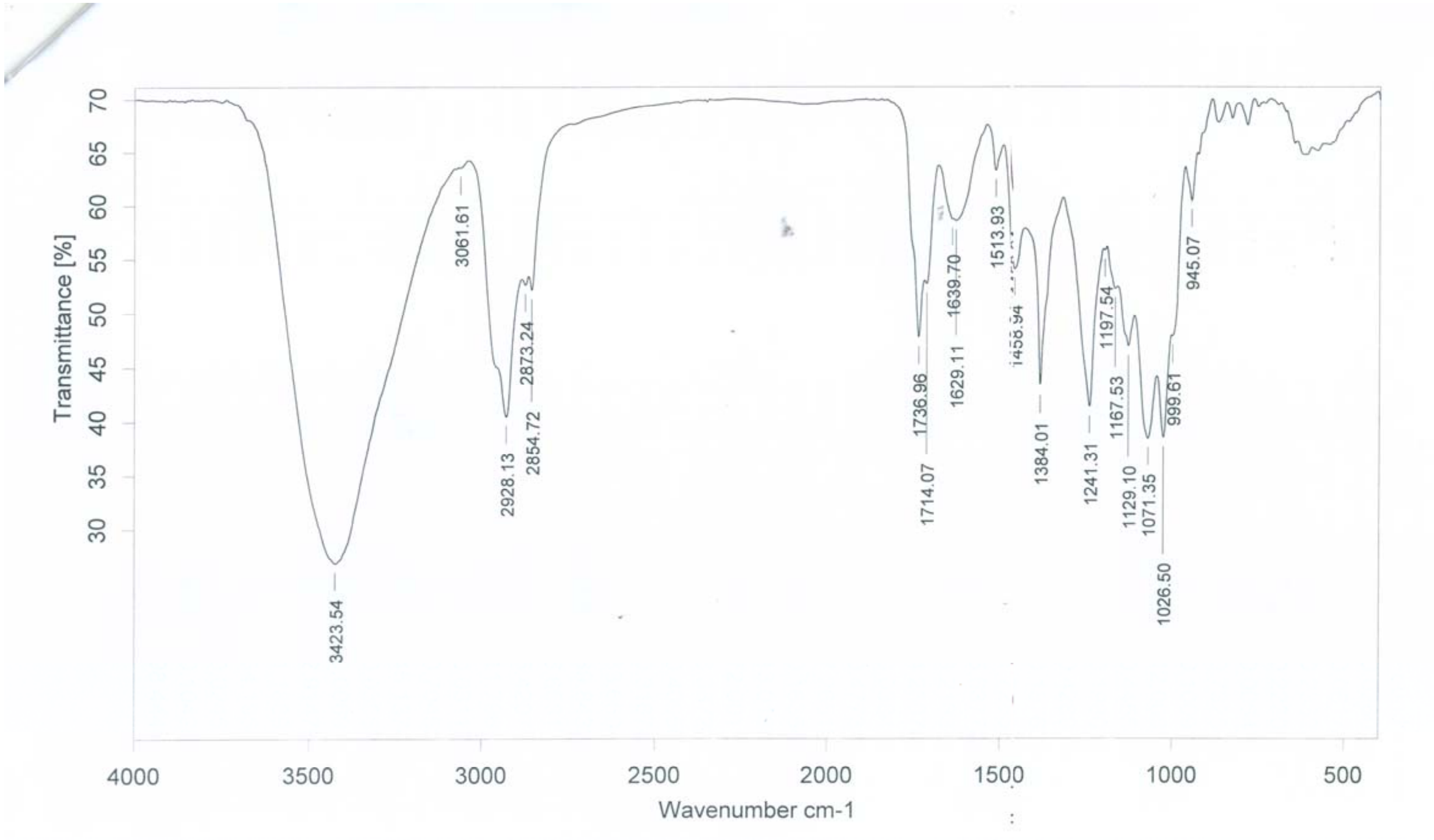

Figure S49. ^{1}H NMR Spectrum of **7** in Pyridine-d_5

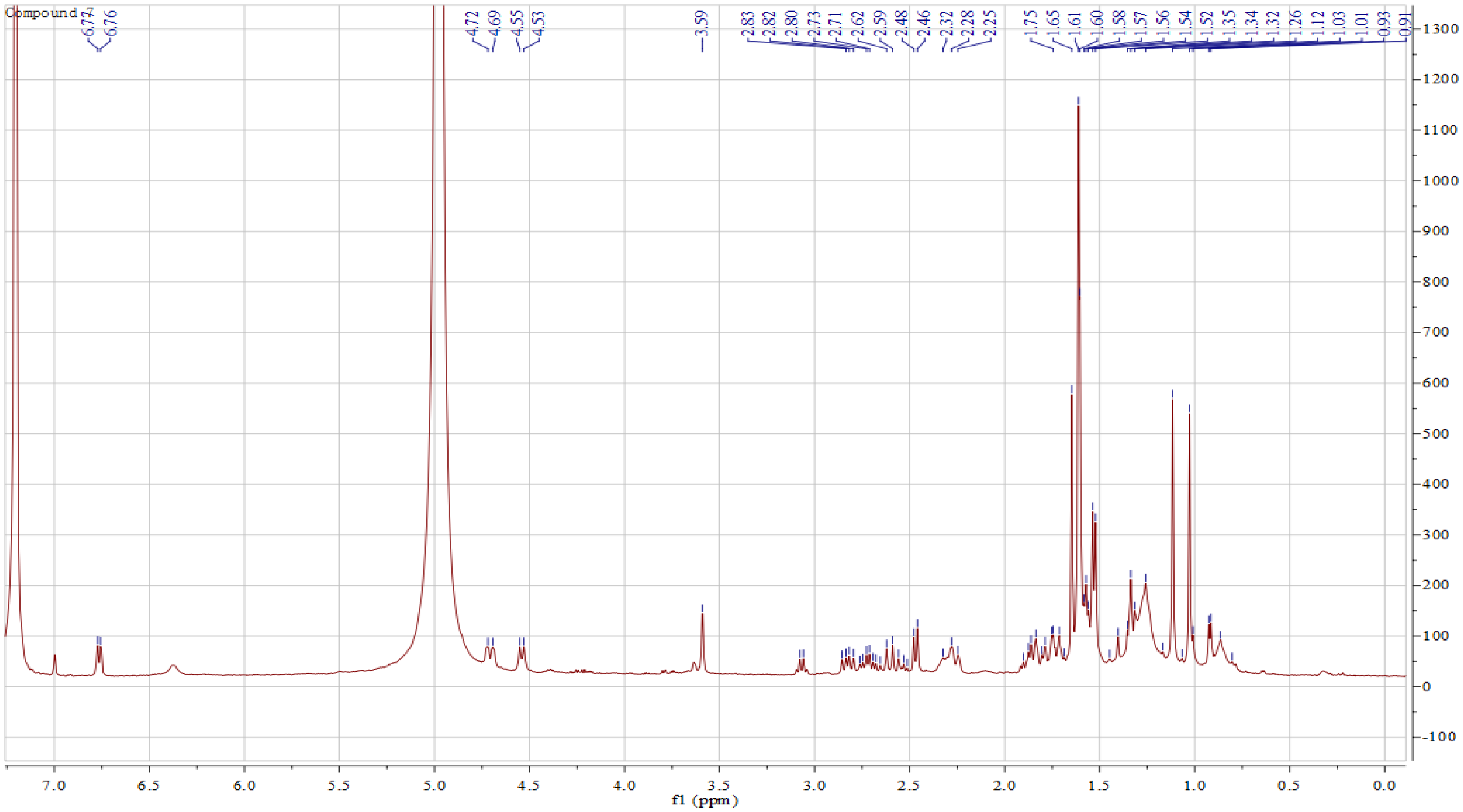

Figure S50. ^{13}C NMR Spectrum of **7** in Pyridine-d_5

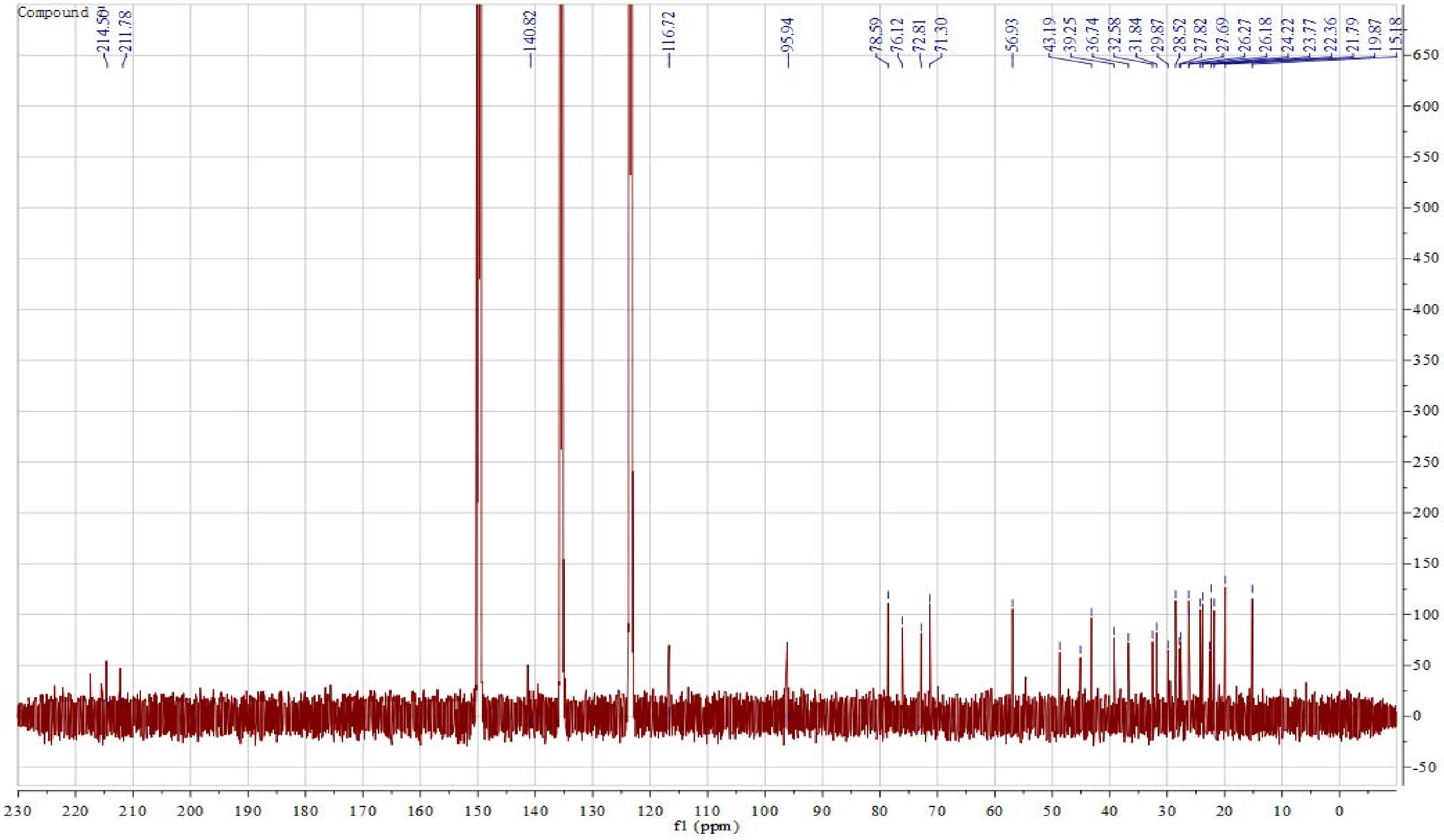

Figure S51. HSQC Spectrum of **7** in Pyridine-*d*5

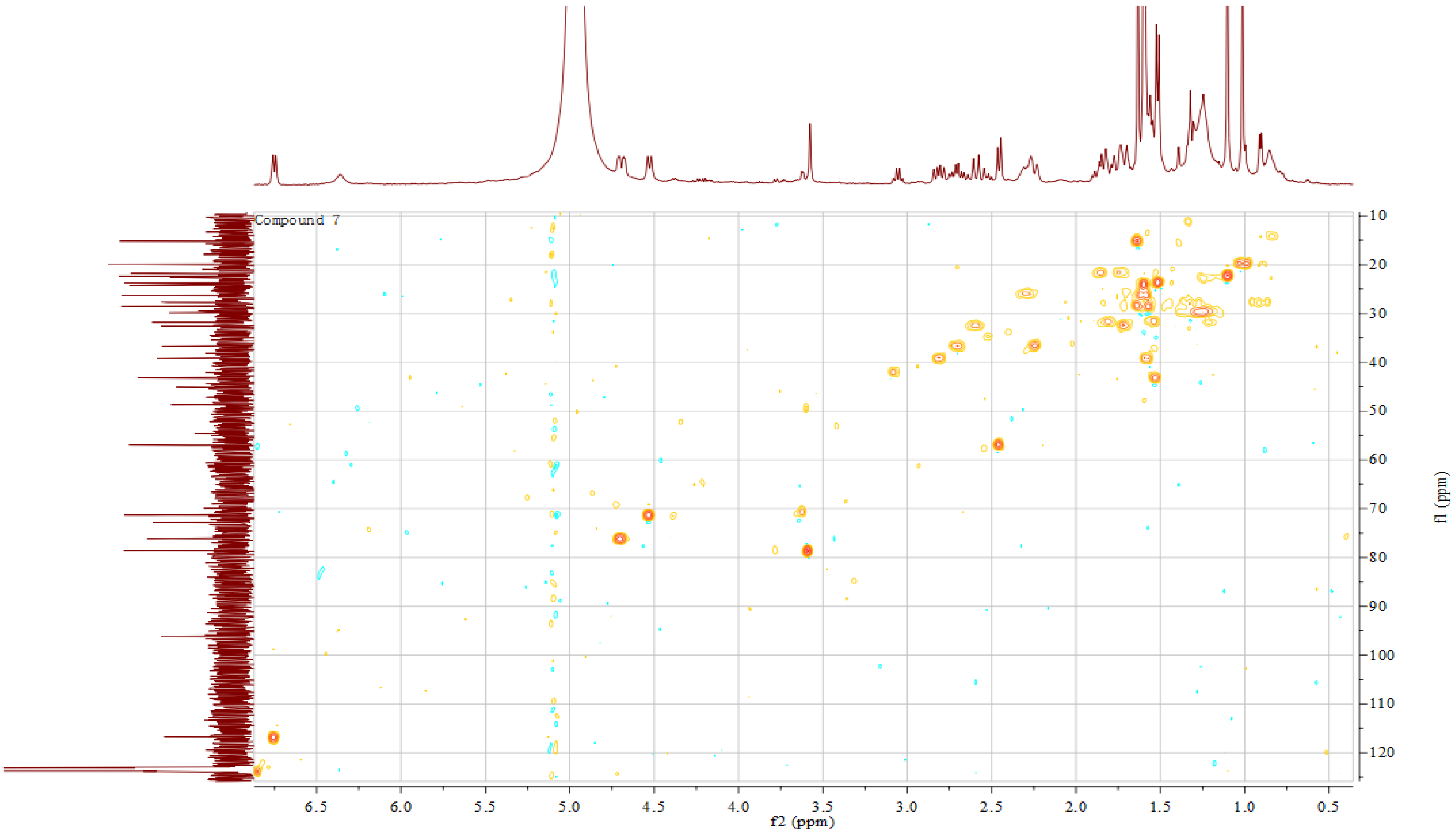

Figure S52. HMBC Spectrum of **7** in Pyridine-*d*$_5$

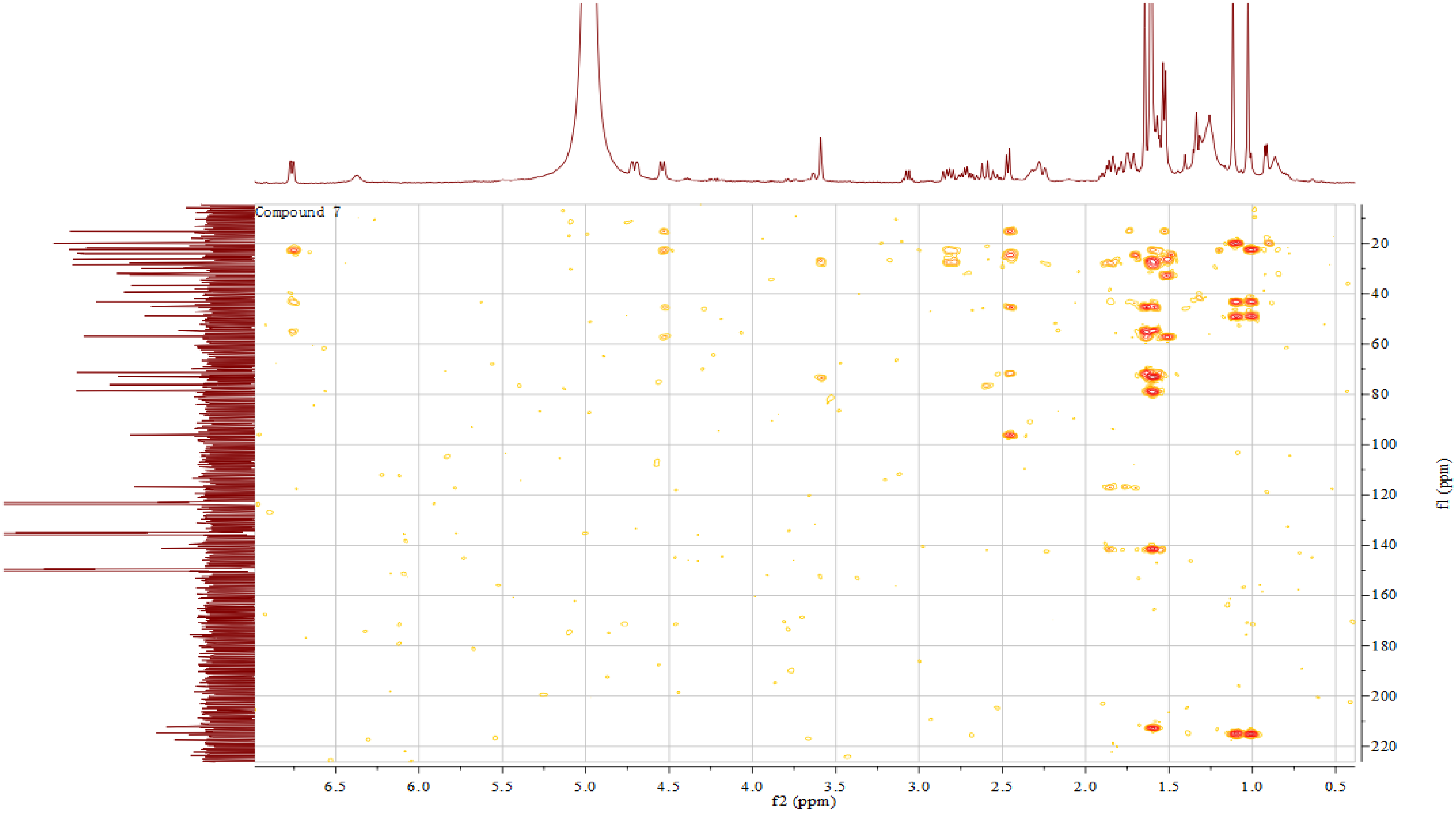

Figure S53. ^{1}H-^{1}H COSY Spectrum of **7** in Pyridine-d_5

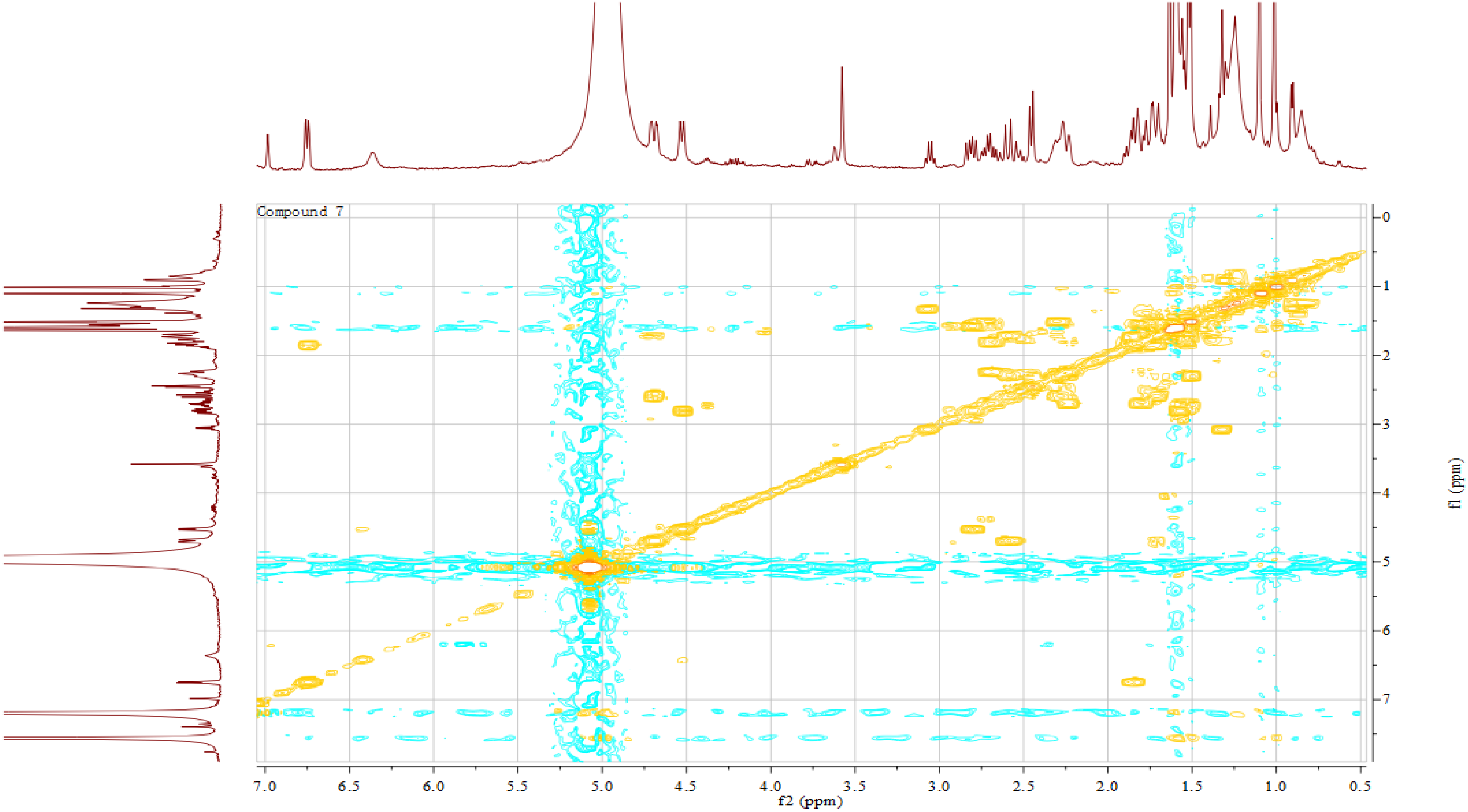

Figure S54. ROESY Spectrum of **7** in Pyridine-d_5

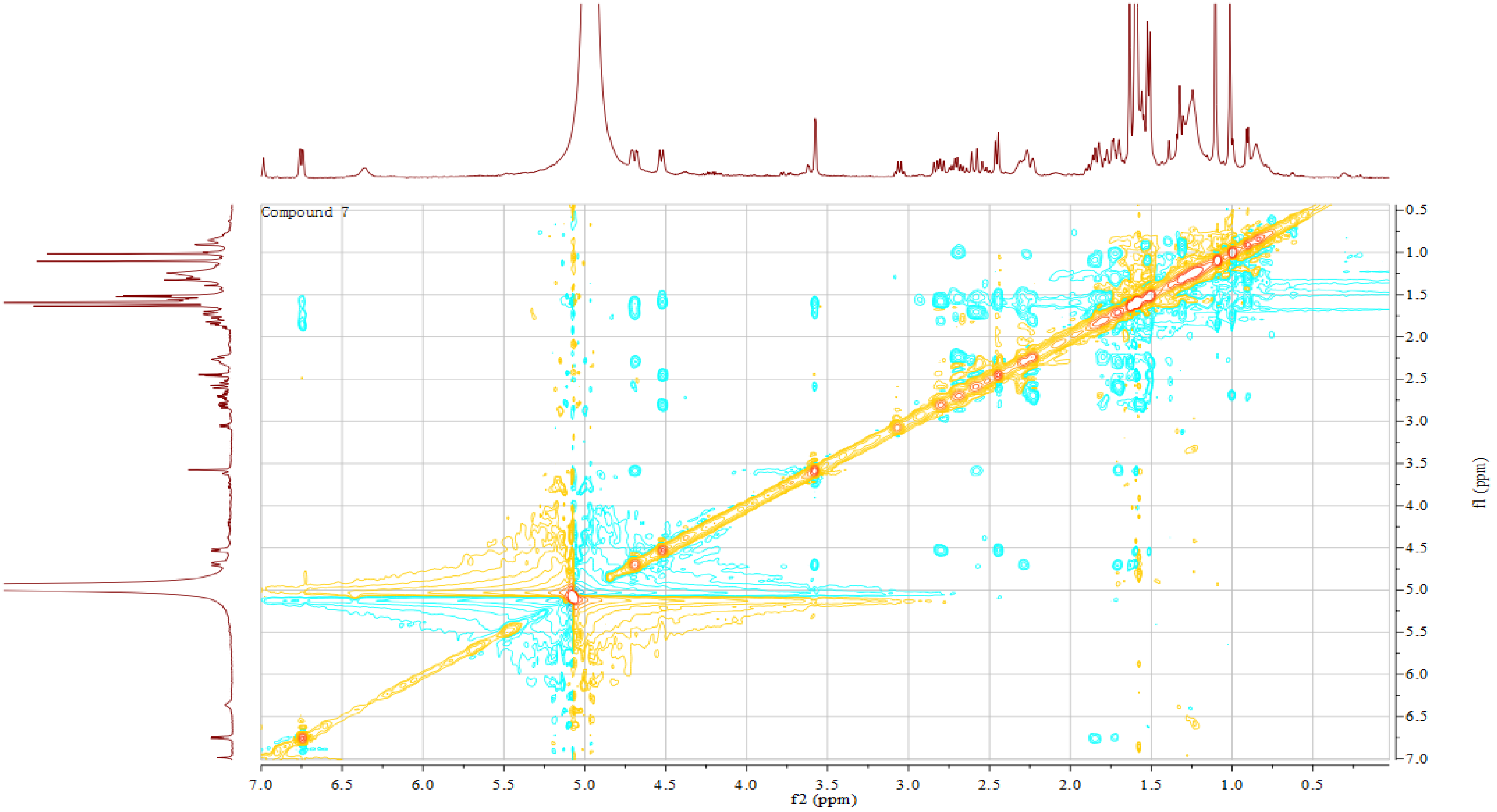

Figure S55. HRESIMS of **7**

Qualitative Analysis Report

**Data Filename**		**Sample Name**	
**Sample Type**	Sample	**Position**	
**Instrument Name**	Agilent G6230 TOF MS	**User Name**	
**Acq Method**	ESI.m	**Acquired Time**	
**IRM Calibration Status**	Success	**DA Method**	ESI.m
**Comment**			

**Sample Group**		**Info.**
**Acquisition SW Version**	6200 series TOF/6500 series Q-TOF B.05.01 (B5125.2)	

User Spectra

Fragmentor Voltage	Collision Energy	Ionization Mode
200	0	ESI

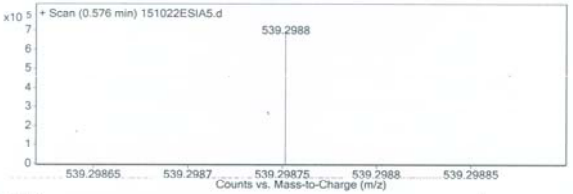

Peak List

m/z	z	Abund	Formula	Ion
517.371	1	74801.61		
531.3865	1	86222.37		
539.2988	1	696356	C30 H44 Na O7	M+
540.3018	1	213360.53	C30 H44 Na O7	M+
541.3096	1	80991.41	C30 H44 Na O7	M+
561.3972	1	80713.26		
575.4124	1	88325.02		
1055.6074	1	367616.97		
1056.6104	1	232328.45		
1057.6166	1	130736.3		

Formula Calculator Element Limits

Element	Min	Max
C	0	200
H	0	400
O	1	12
Na	1	1

Formula Calculator Results

Formula	CalculatedMass	CalculatedMz	Mz	Diff. (mDa)	Diff. (ppm)	DBE
C30 H44 Na O7	539.2985	539.2979	539.2988	[illegible]	[illegible]	8.5000

--- End Of Report ---

Figure S56. IR Spectrum of **7**

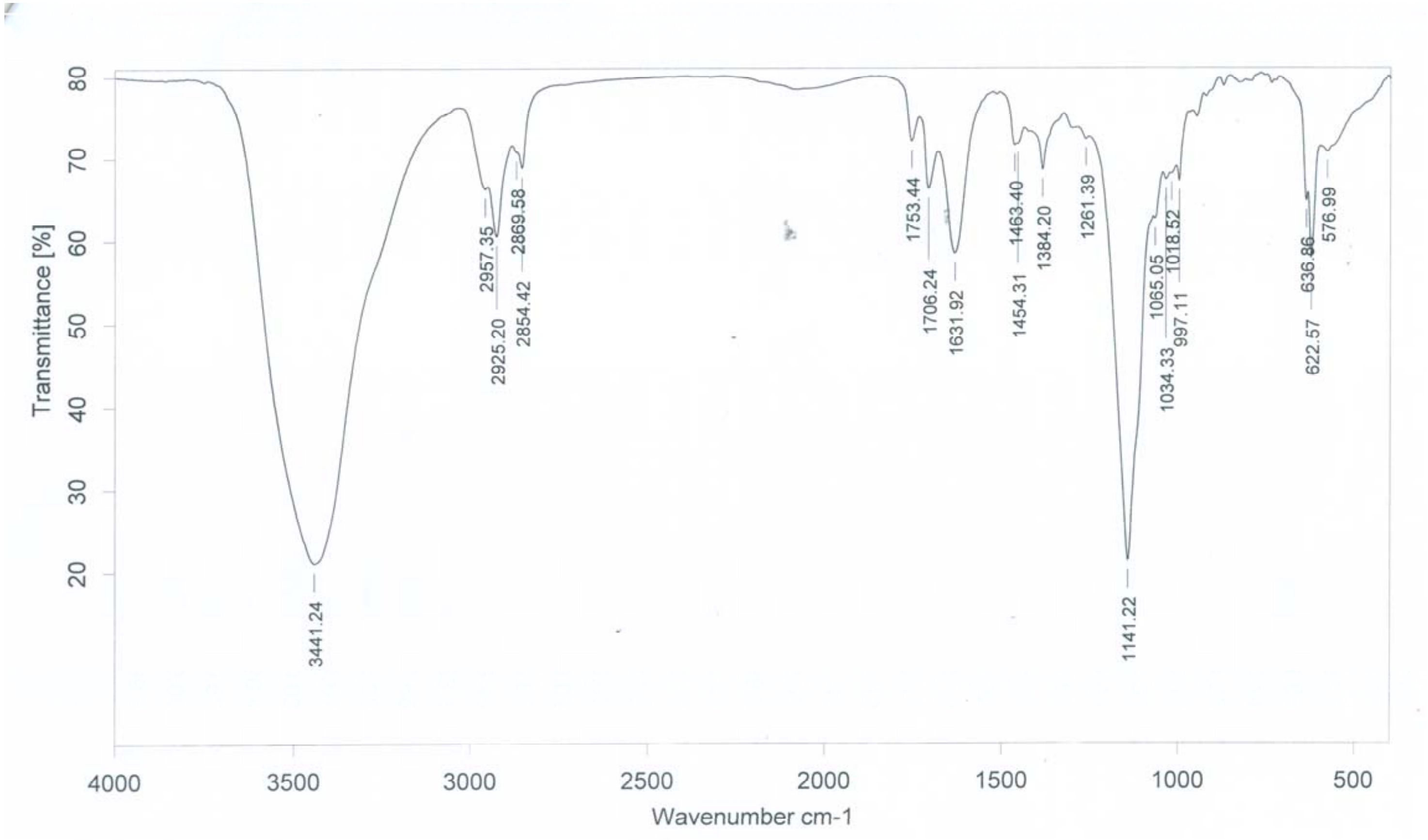

Figure S57. ^{1}H NMR Spectrum of **8** in Pyridine-d_5

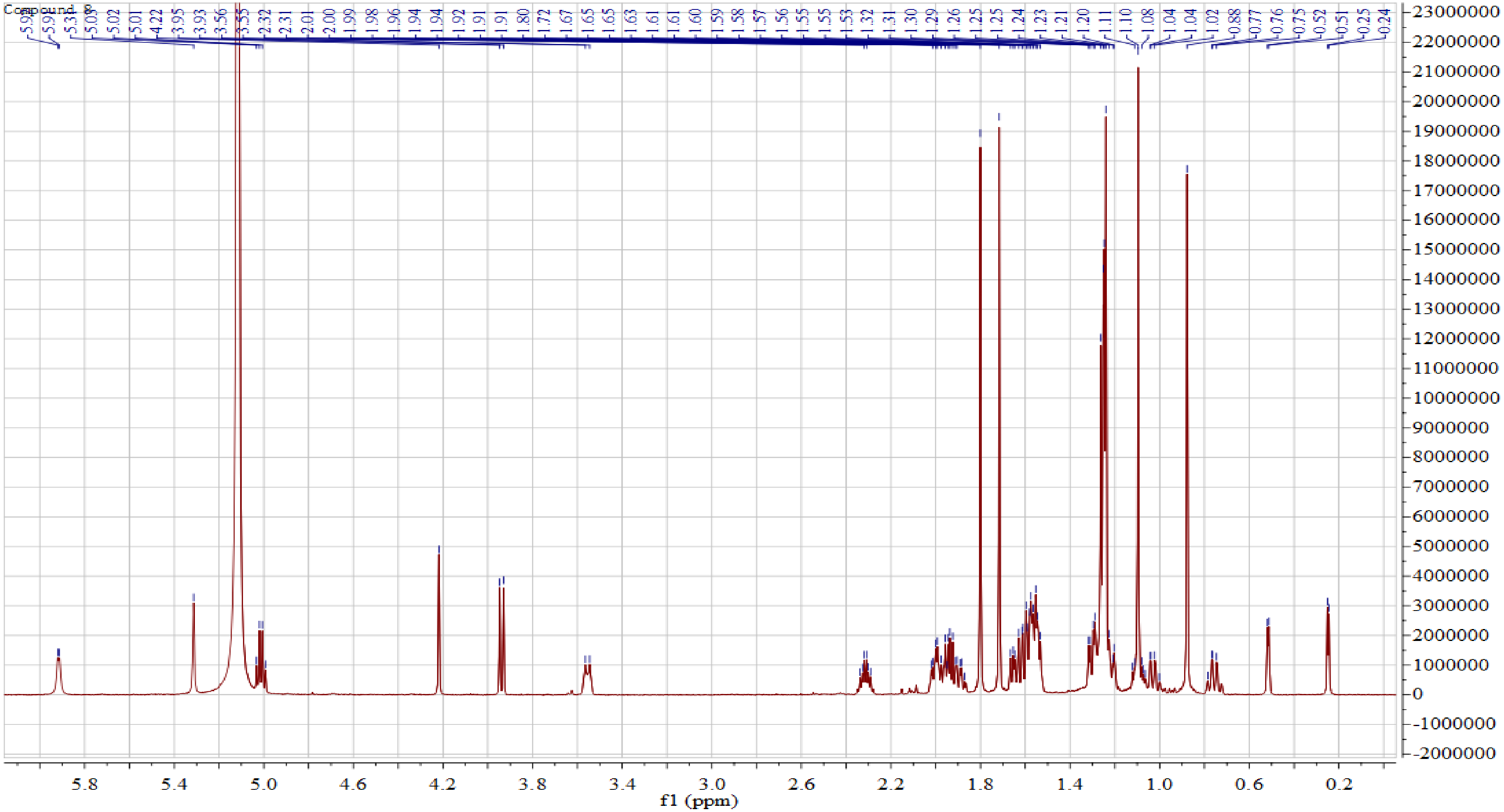

Figure S58 ^{13}C NMR Spectrum of **8** in Pyridine-d_5

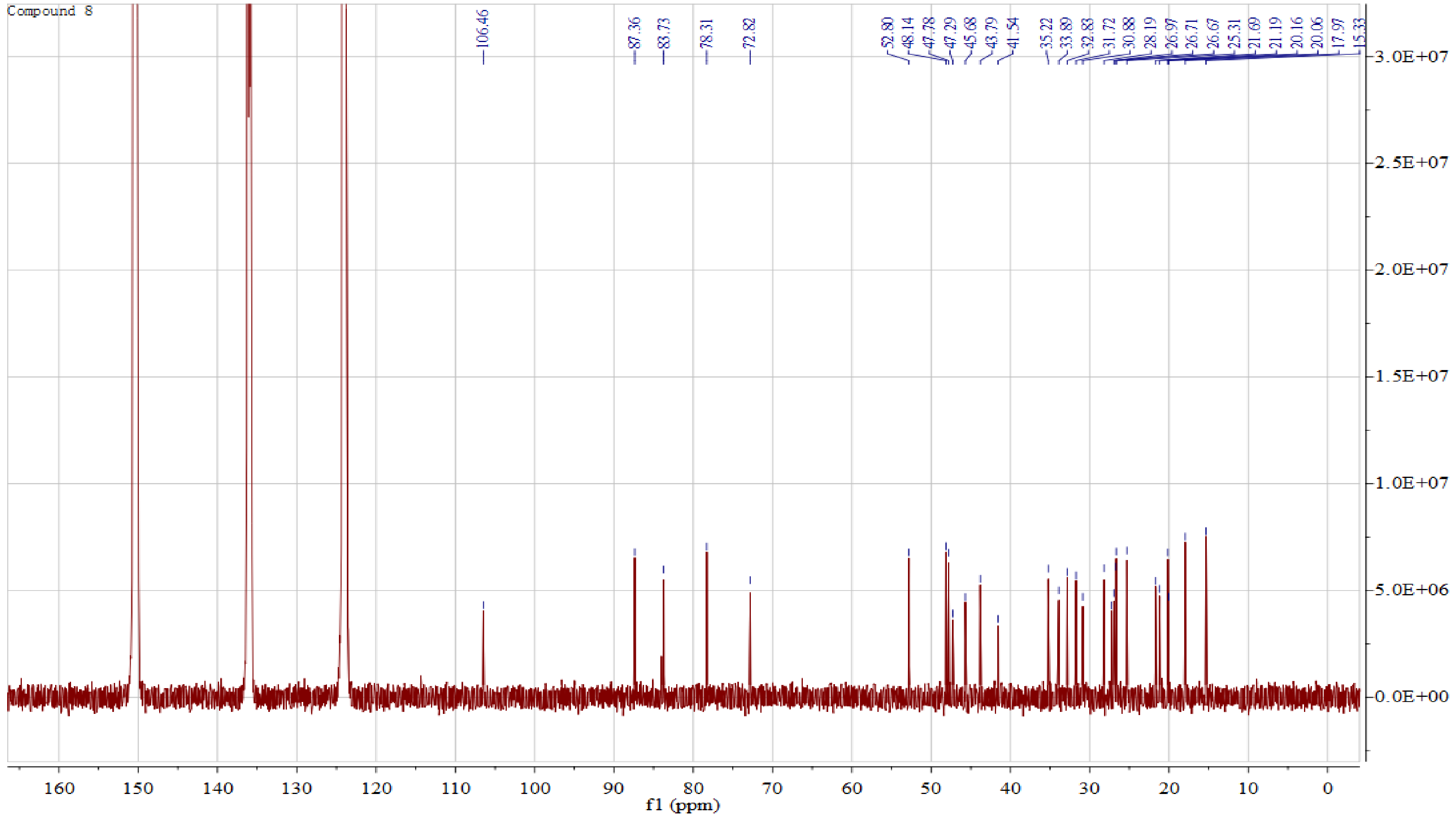

Figure S59. HSQC Spectrum of **8** in Pyridine-d_5

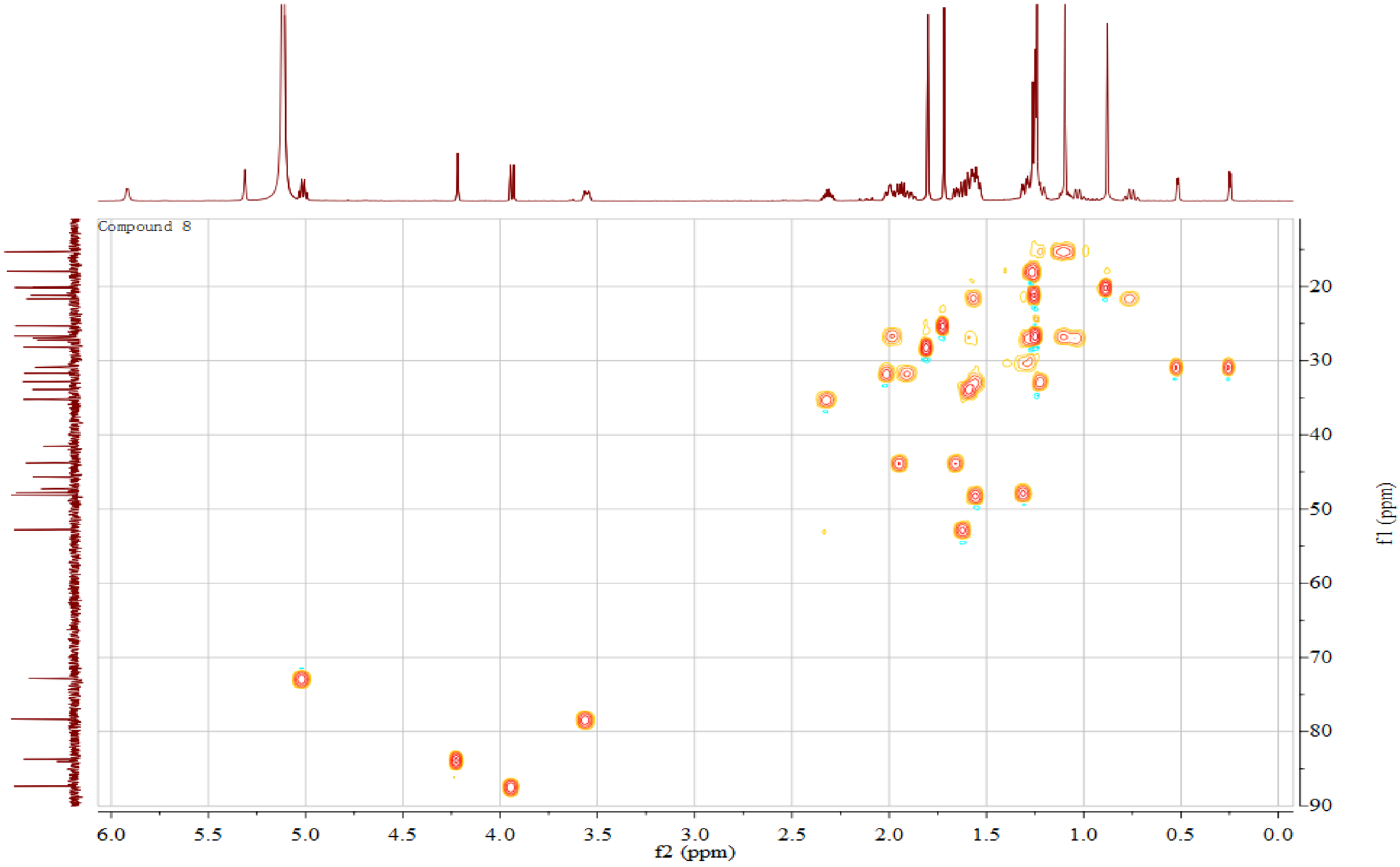

Figure S60. HMBC Spectrum of **8** in Pyridine-d_5

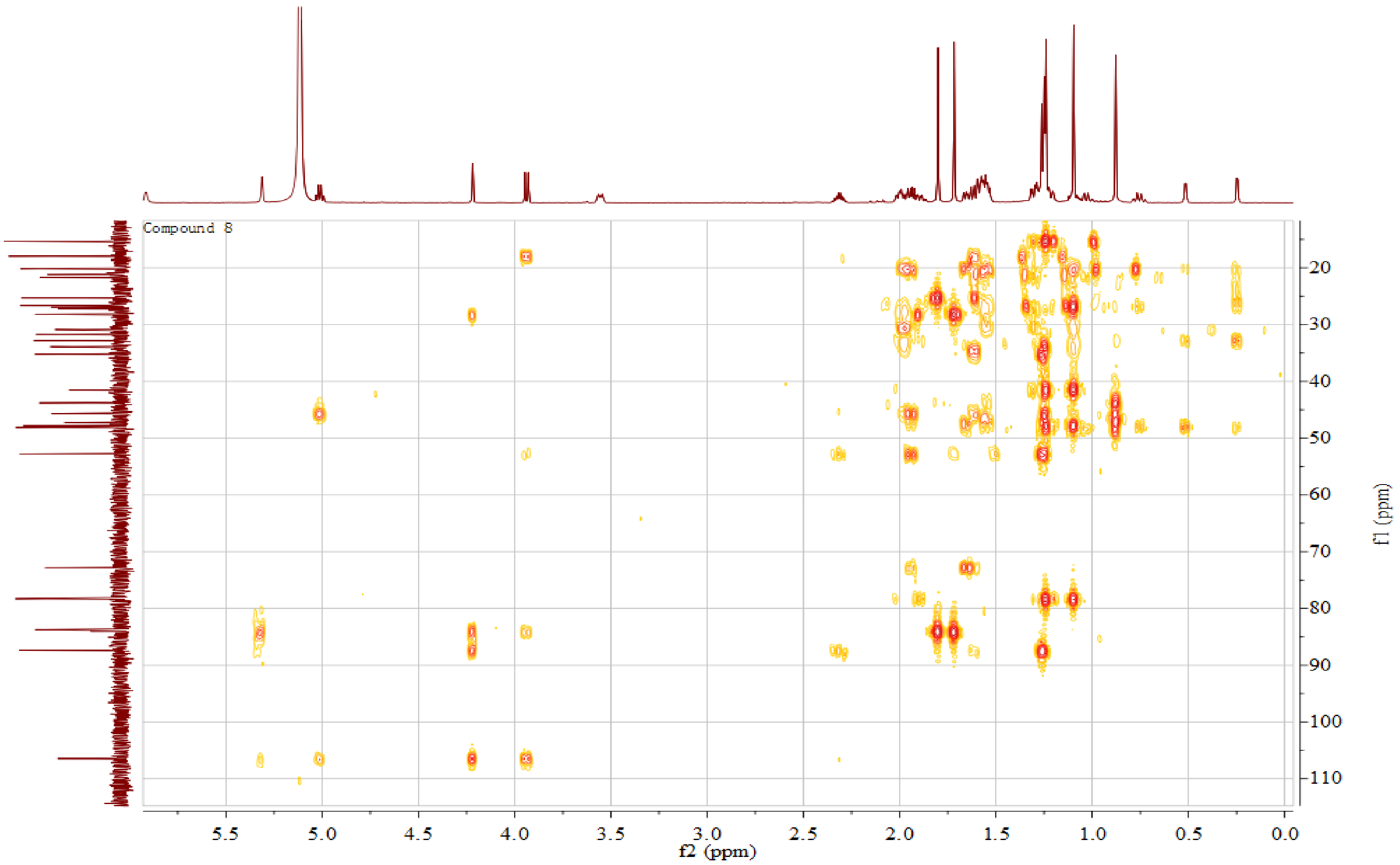

Figure S61. ^{1}H-^{1}H COSY Spectrum of **8** in Pyridine-d_5

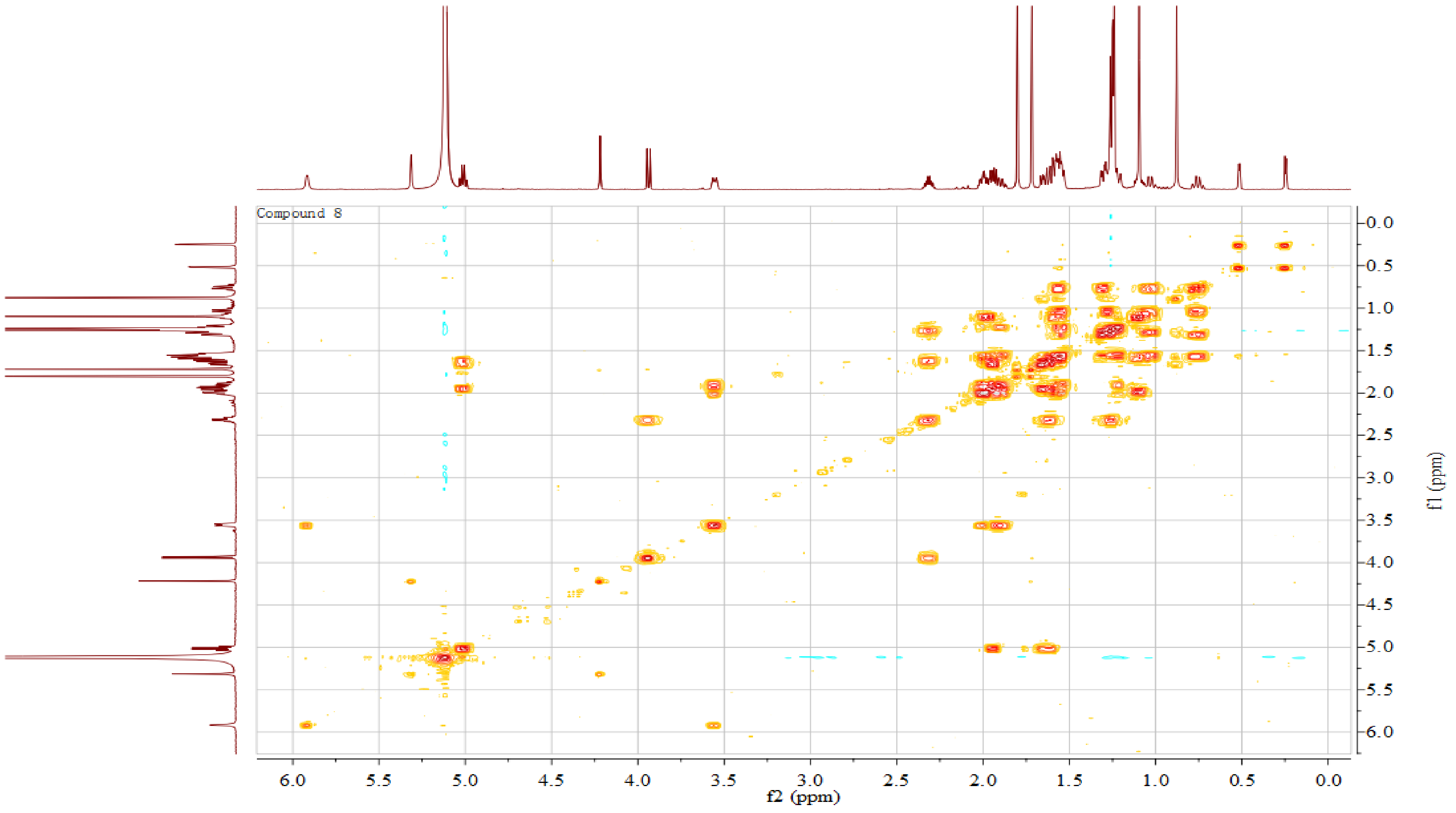

Figure S62. ROESY Spectrum of **8** in Pyridine-d_5

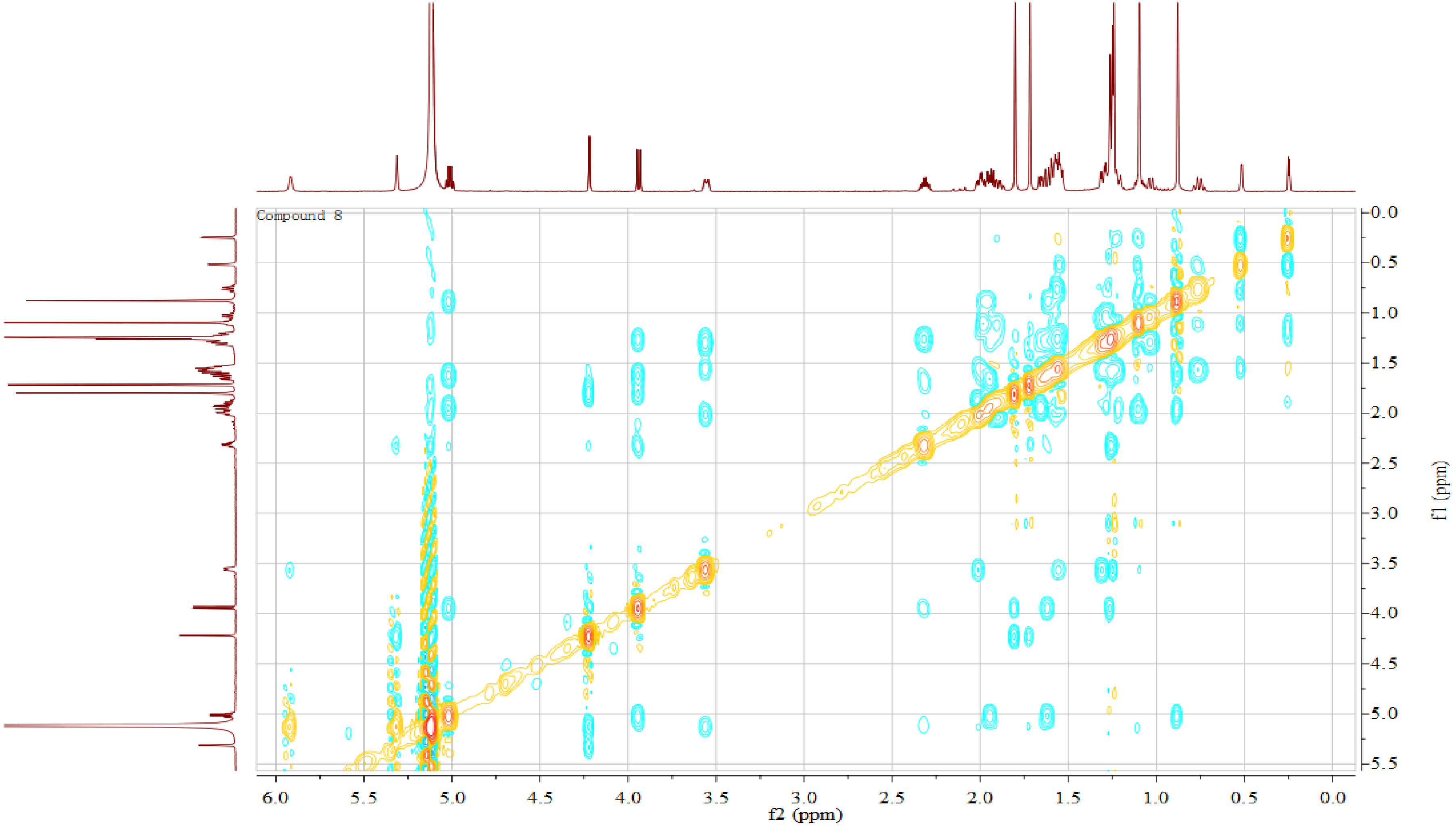

Figure S63. HRESIMS of **8**

Elemental Composition Report

Page 1

Single Mass Analysis

Tolerance = 10.0 PPM / DBE: min = -10.0, max = 120.0

Selected filters: None

Monoisotopic Mass, Odd and Even Electron Ions

21 formula(e) evaluated with 1 results within limits (up to 51 closest results for each mass)

Elements Used:

C: 0-200 H: 0-400 O: 4-6

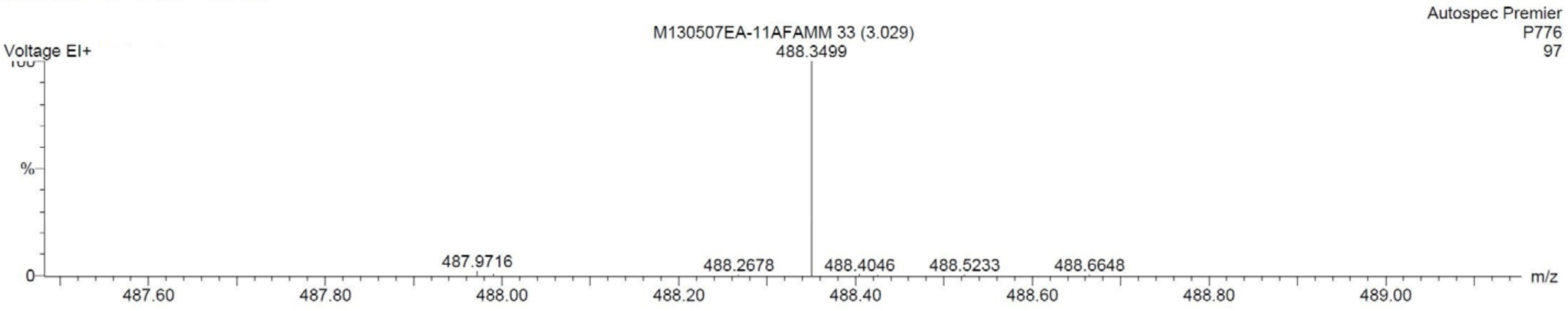

Minimum:				-10.0		
Maximum:		200.0	10.0	120.0		

Mass	Calc. Mass	mDa	PPM	DBE	i-FIT	Formula
488.3499	488.3502	-0.3	-0.6	7.0	5546067.0	C30 H48 O5

Figure S64. IR Spectrum of **8**

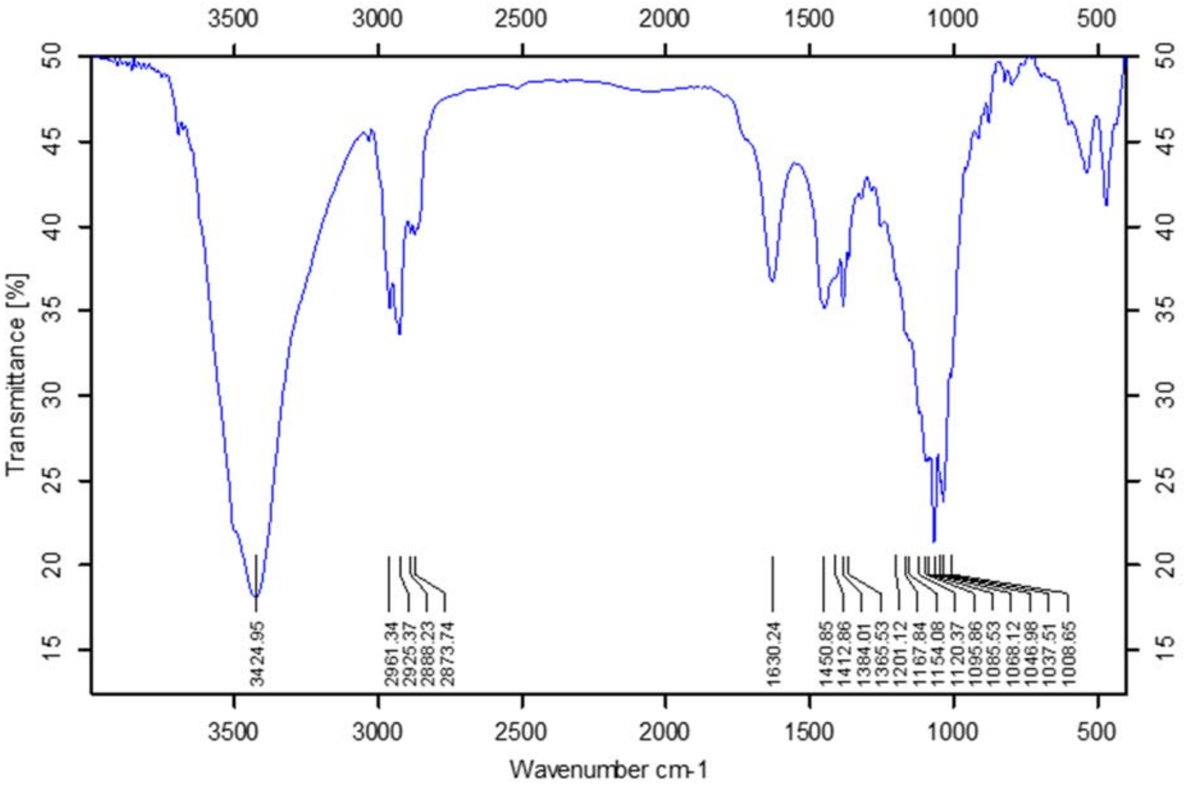

Figure S65. ^{1}H NMR Spectrum of **9** in Pyridine-d_5

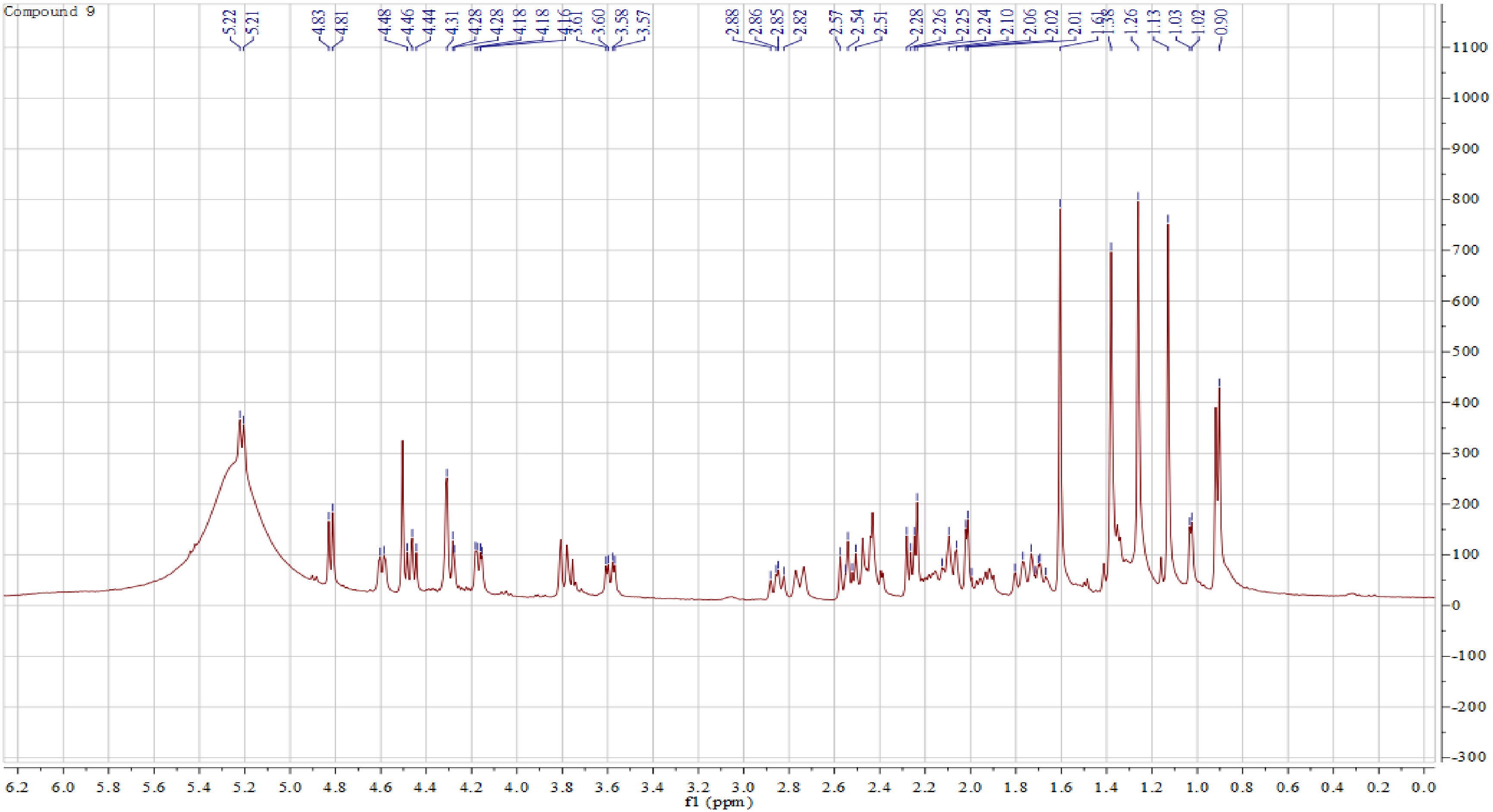

Figure S66 ^{13}C NMR Spectrum of **9** in Pyridine-d_5

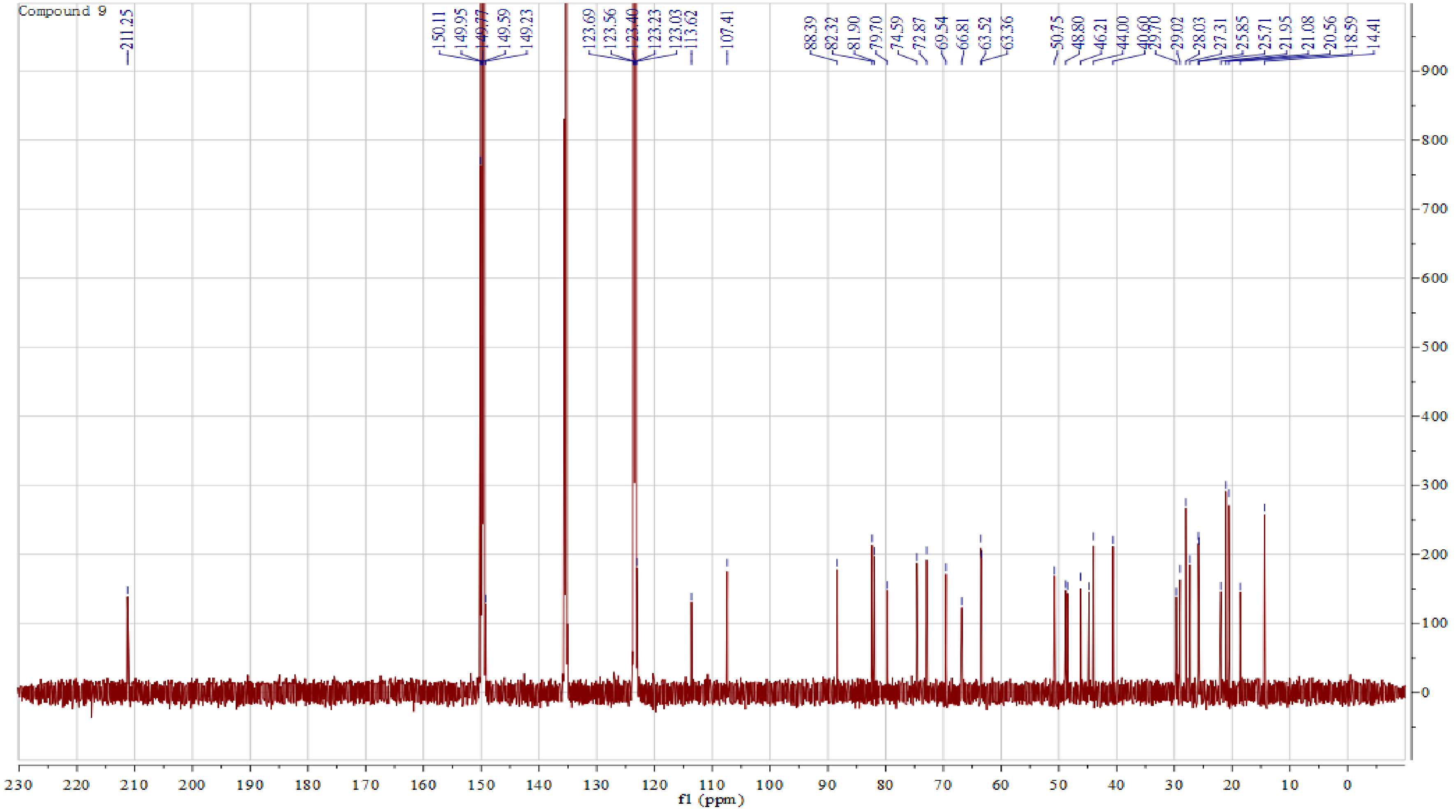

Figure S67. HSQC Spectrum of **9** in Pyridine-d_5

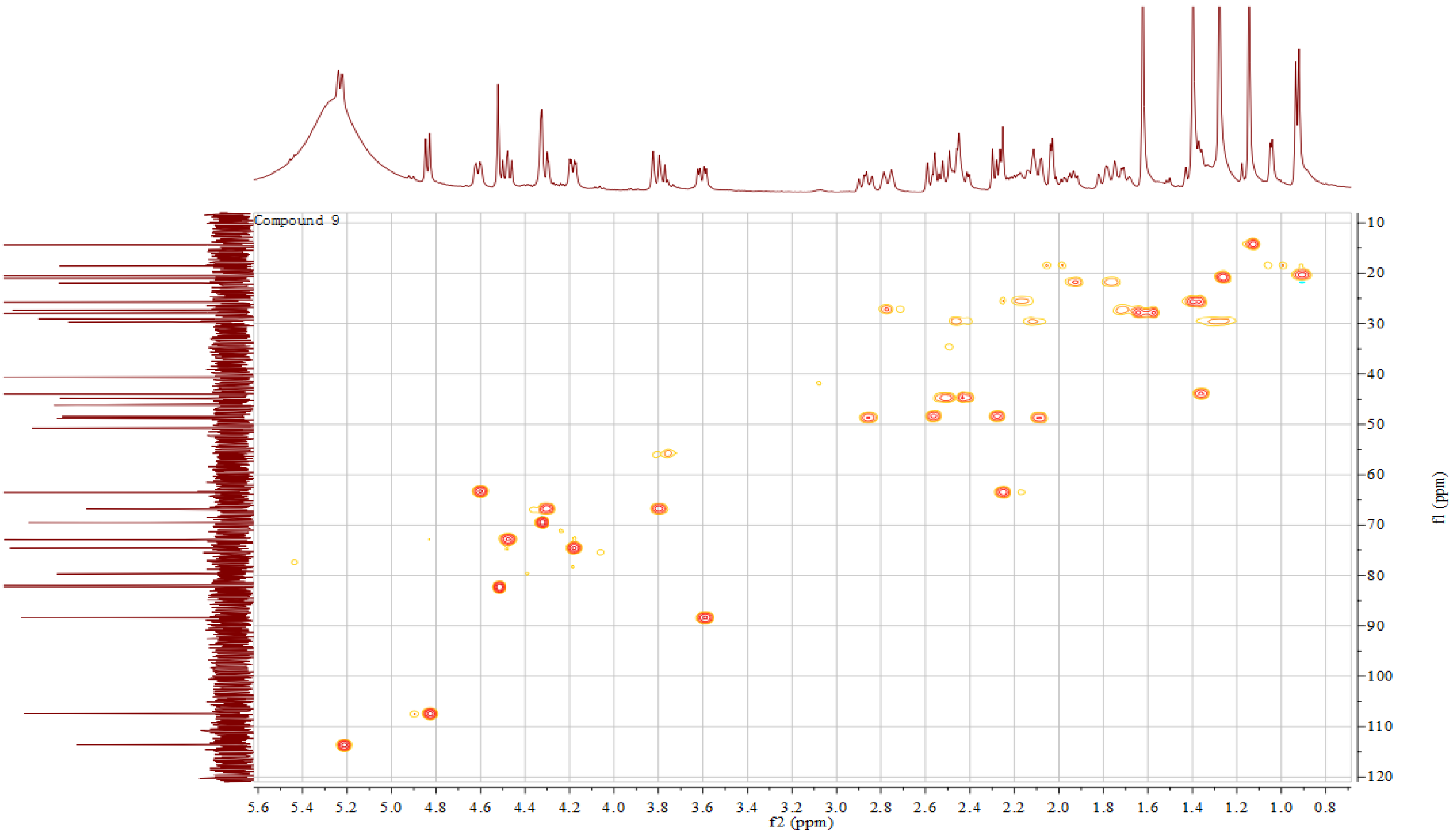

Figure S68. HMBC Spectrum of **9** in Pyridine-d_5

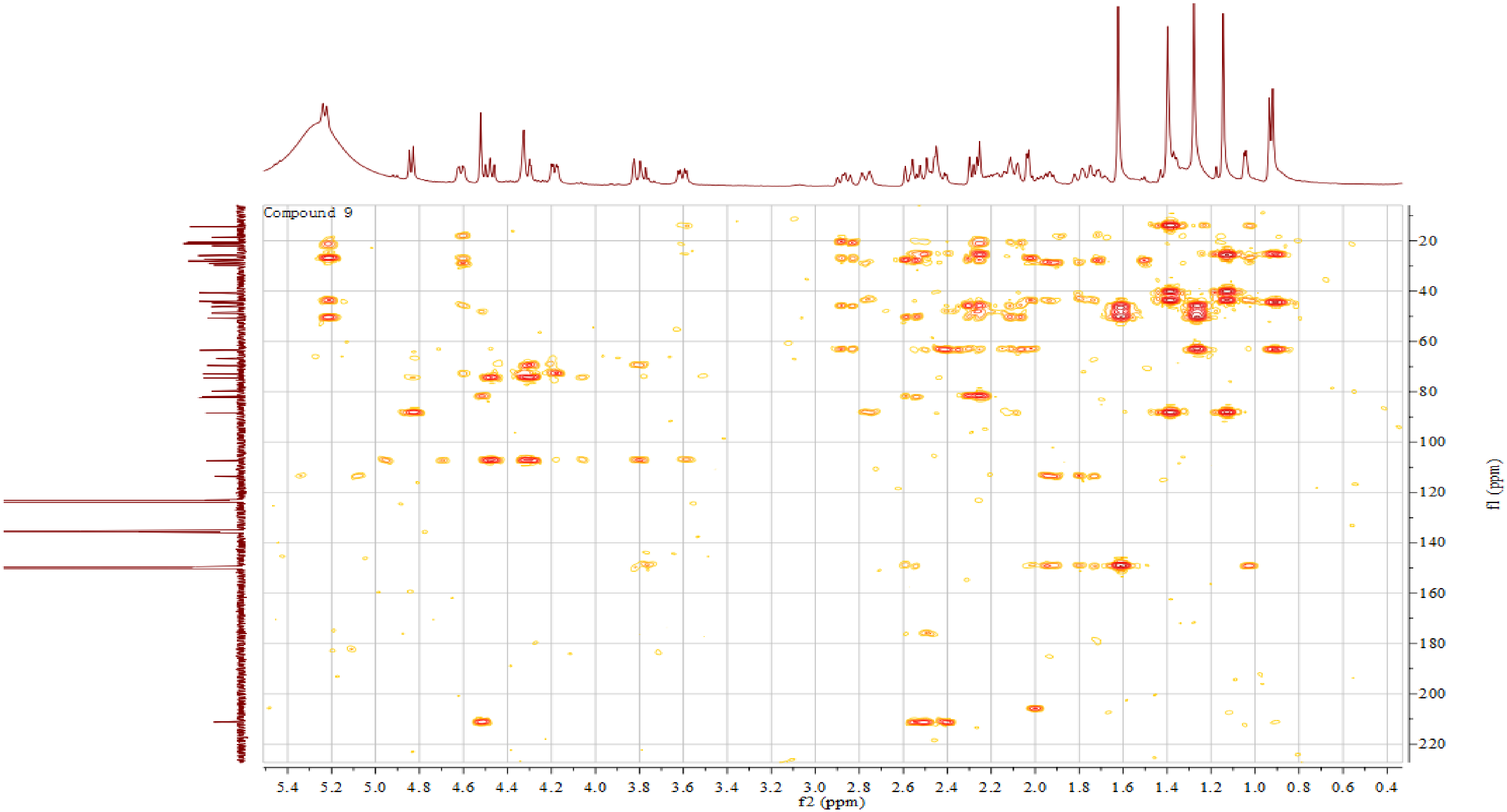

Figure S69. ^{1}H-^{1}H COSY Spectrum of **9** in Pyridine-d_5

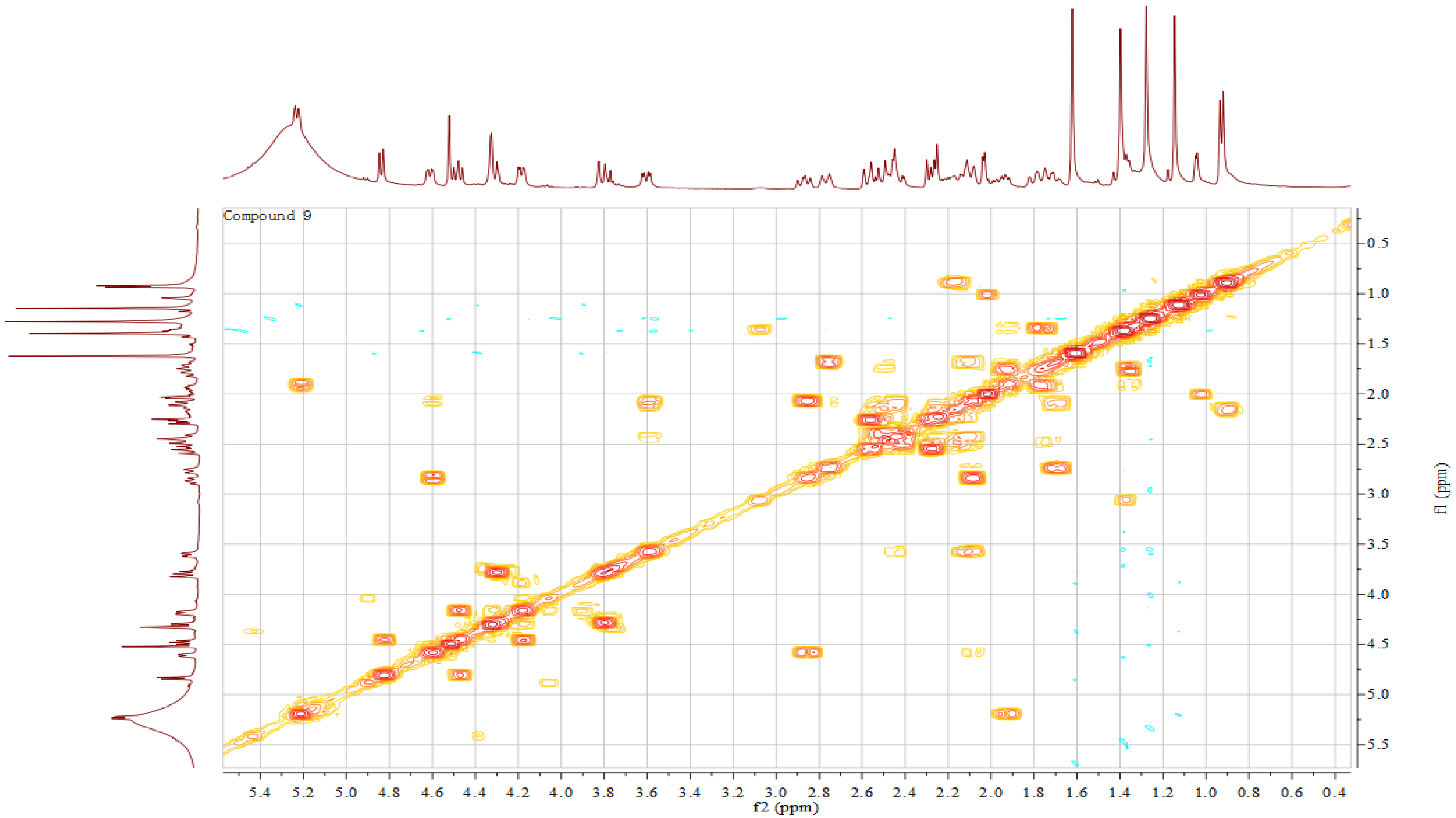

Figure S70. ROESY Spectrum of **9** in Pyridine-d_5

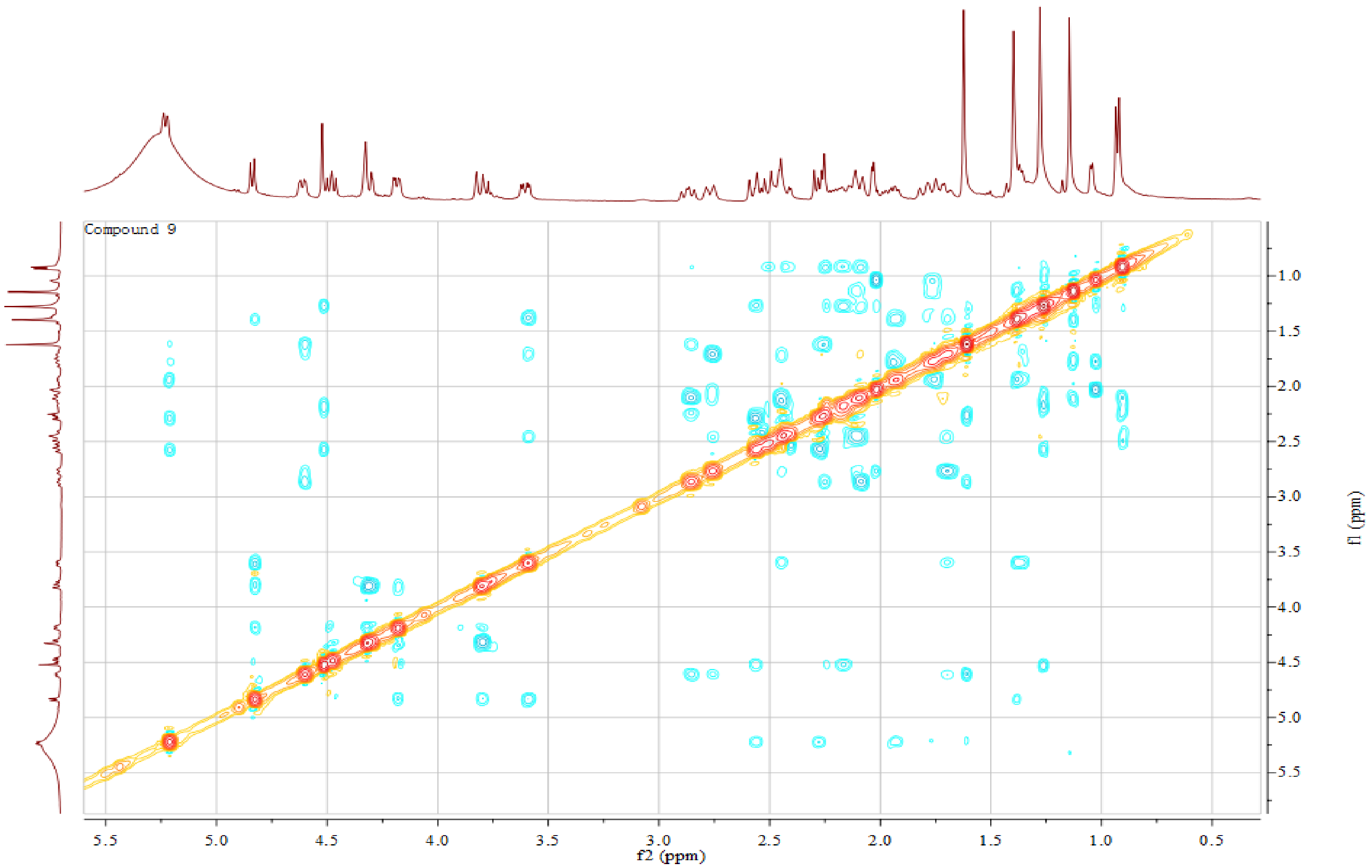

Figure S71. HRESIMS of **9**

Qualitative Analysis Report

**Data Filename**	151022ESIA3.d	**Sample Name**	
**Sample Type**	Sample	**Position**	
**Instrument Name**	Agilent G6230 TOF MS	**User Name**	KIB
**Acq Method**	ESI.m	**Acquired Time**	10/20/2015 5:51:34 PM
**IRM Calibration Status**	Success	**DA Method**	ESI.m
**Comment**			

**Sample Group**		**Info.**
**Acquisition SW Version**	6200 series TOF/6500 series Q-TOF B.05.01 (B5125.2)	

User Spectra

Fragmentor Voltage	Collision Energy	Ionization Mode
200	0	ESI

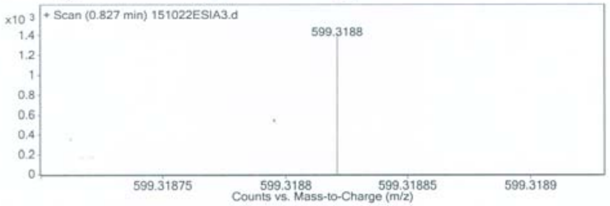

Peak List

m/z	z	Abund
102.1284	1	11537.5
121.0509	1	62614.3
153.0543	1	12896.9
232.1119	1	9774.8
241.1702	1	66952.9
242.173	1	9699.08
299.1101	1	16003.32
301.1409	1	10089.62
922.0098	1	83405.42
923.0109	1	12211.02

Formula Calculator Element Limits

Element	Min	Max
C	0	200
H	0	400
O	1	12
Na	1	1

Formula Calculator Results

Formula	CalculatedMass	CalculatedMz	Mz	Diff. (mDa)	Diff. (ppm)	DBE
C32 H48 Na O9	599.3196	599.3191	599.3188	0.2	0.4	8.5000

--- End Of Report ---

Figure S72. IR Spectrum of **9**

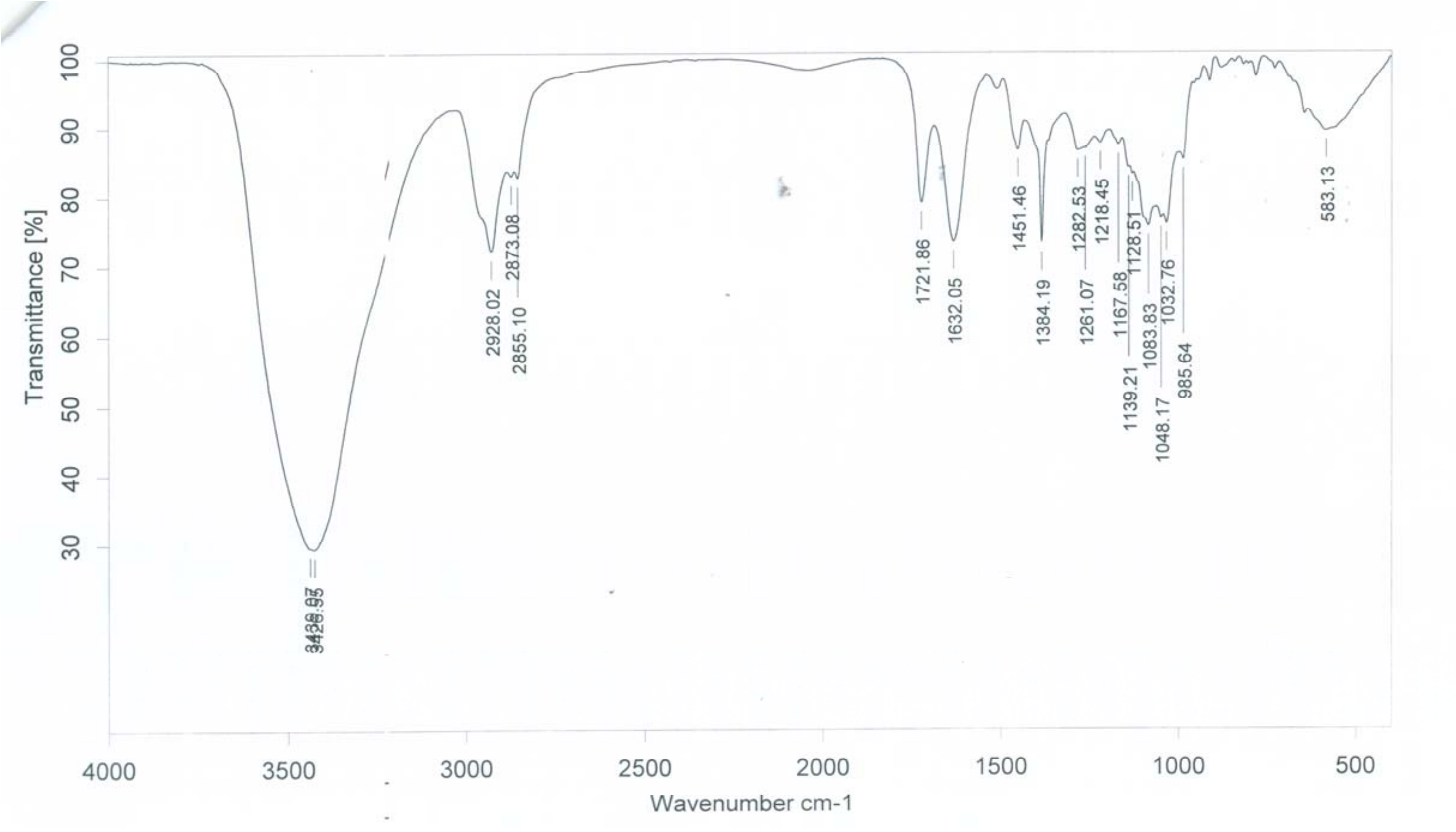

Figure S73. ^{1}H NMR Spectrum of **10** in Pyridine-d_5

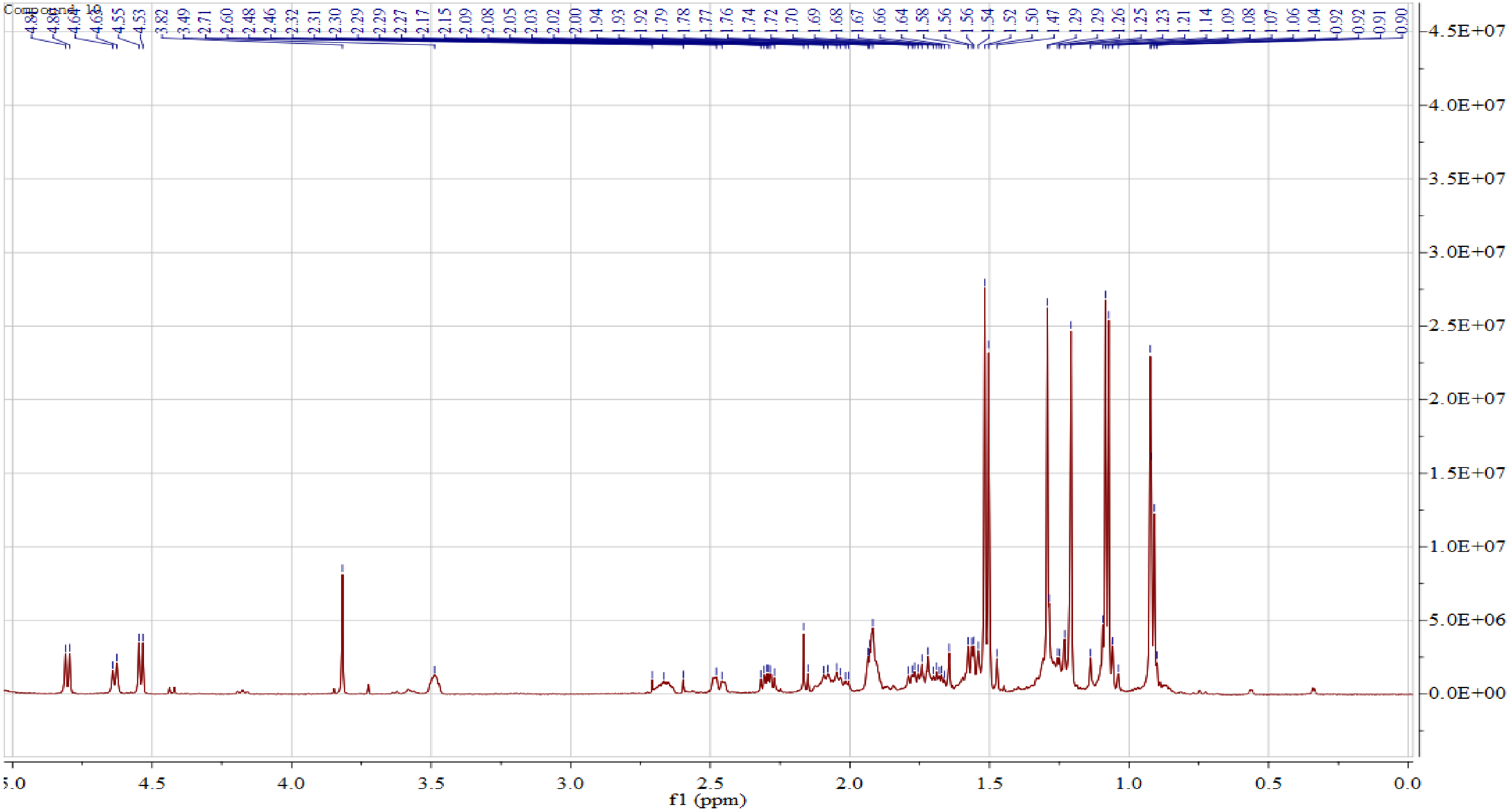

Figure S74 ^{13}C NMR Spectrum of **10** in Pyridine-d_5

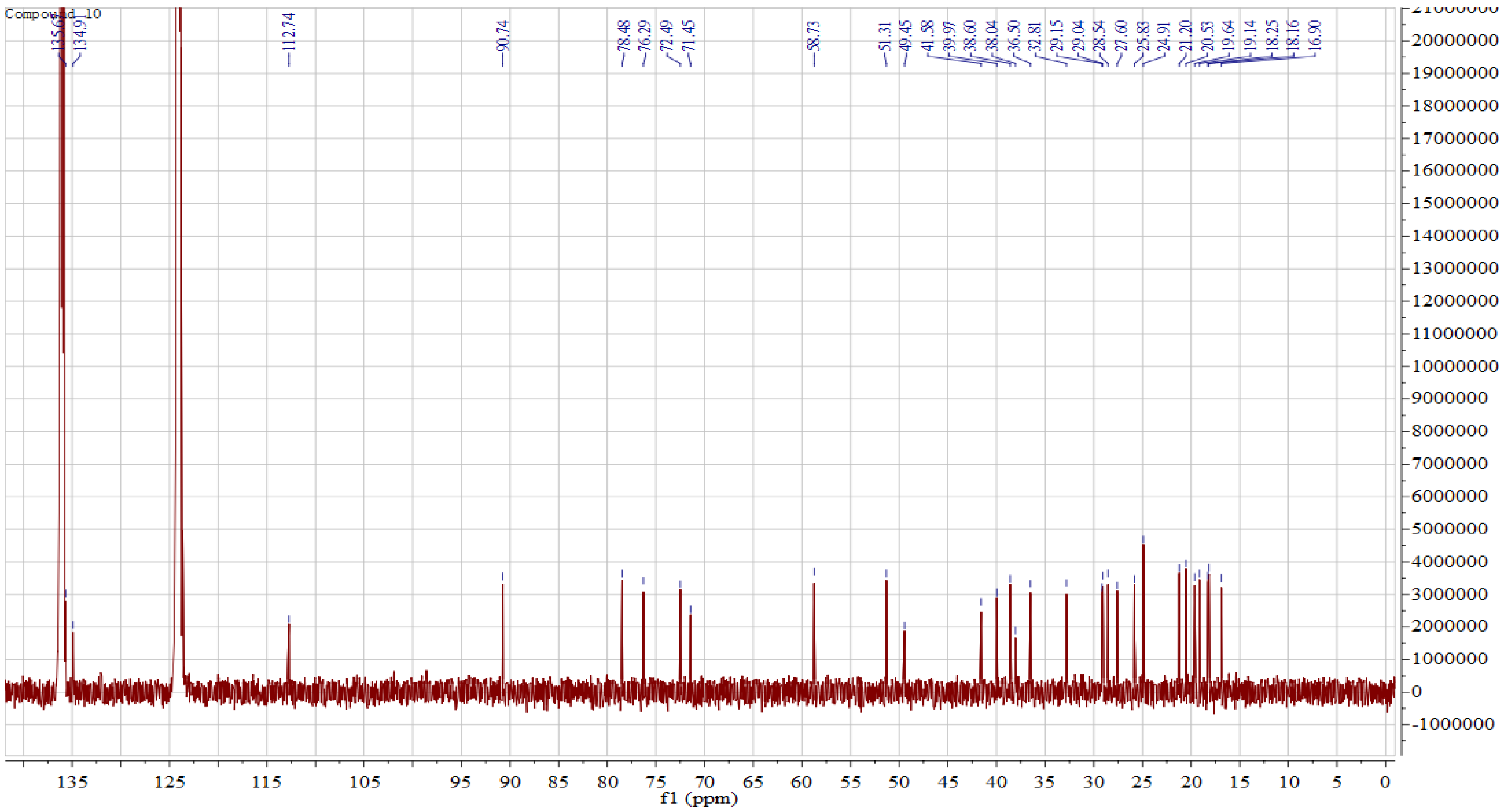

Figure S75. HSQC Spectrum of **10** in Pyridine-d_5

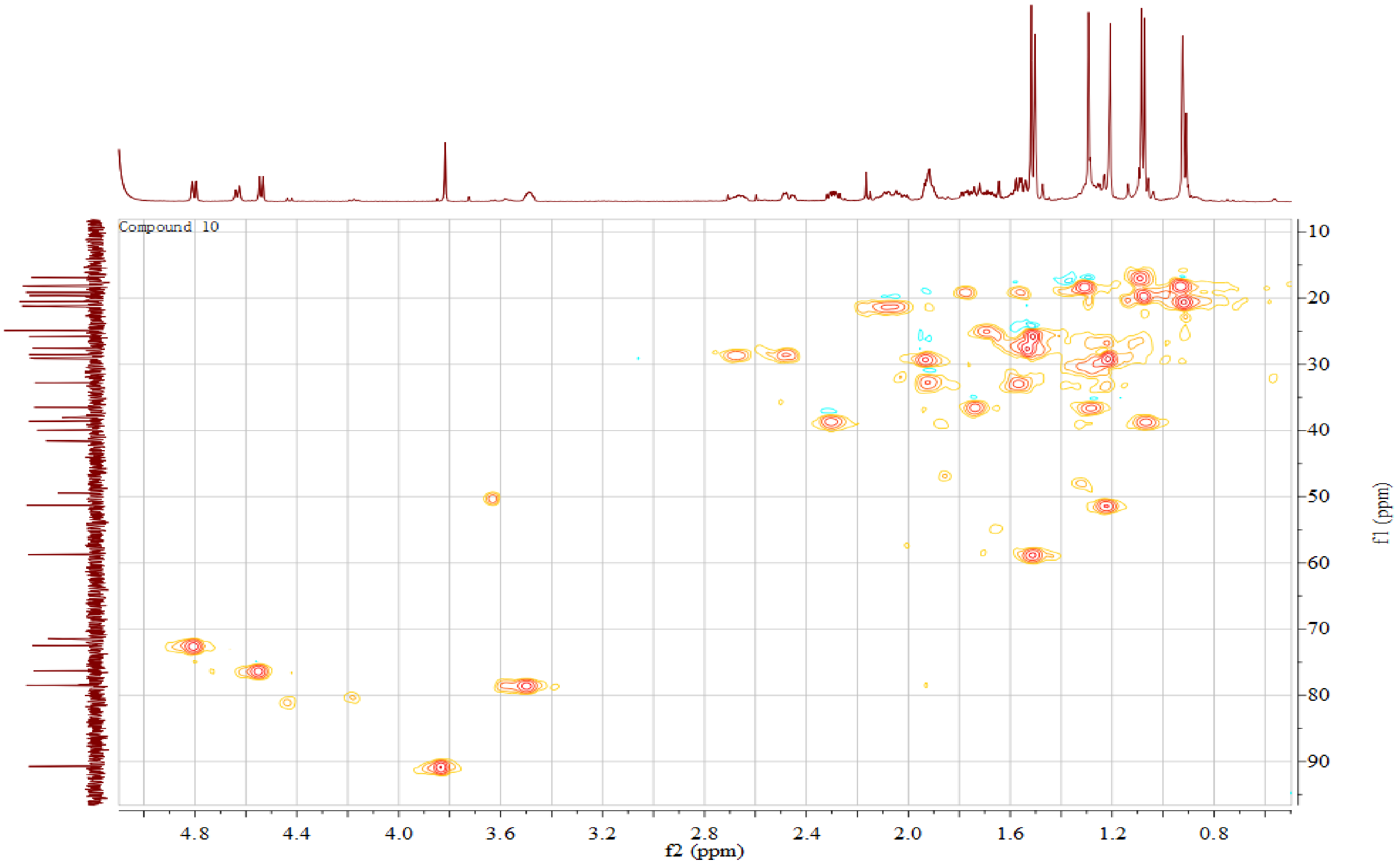

Figure S76. HMBC Spectrum of **10** in Pyridine-d_5

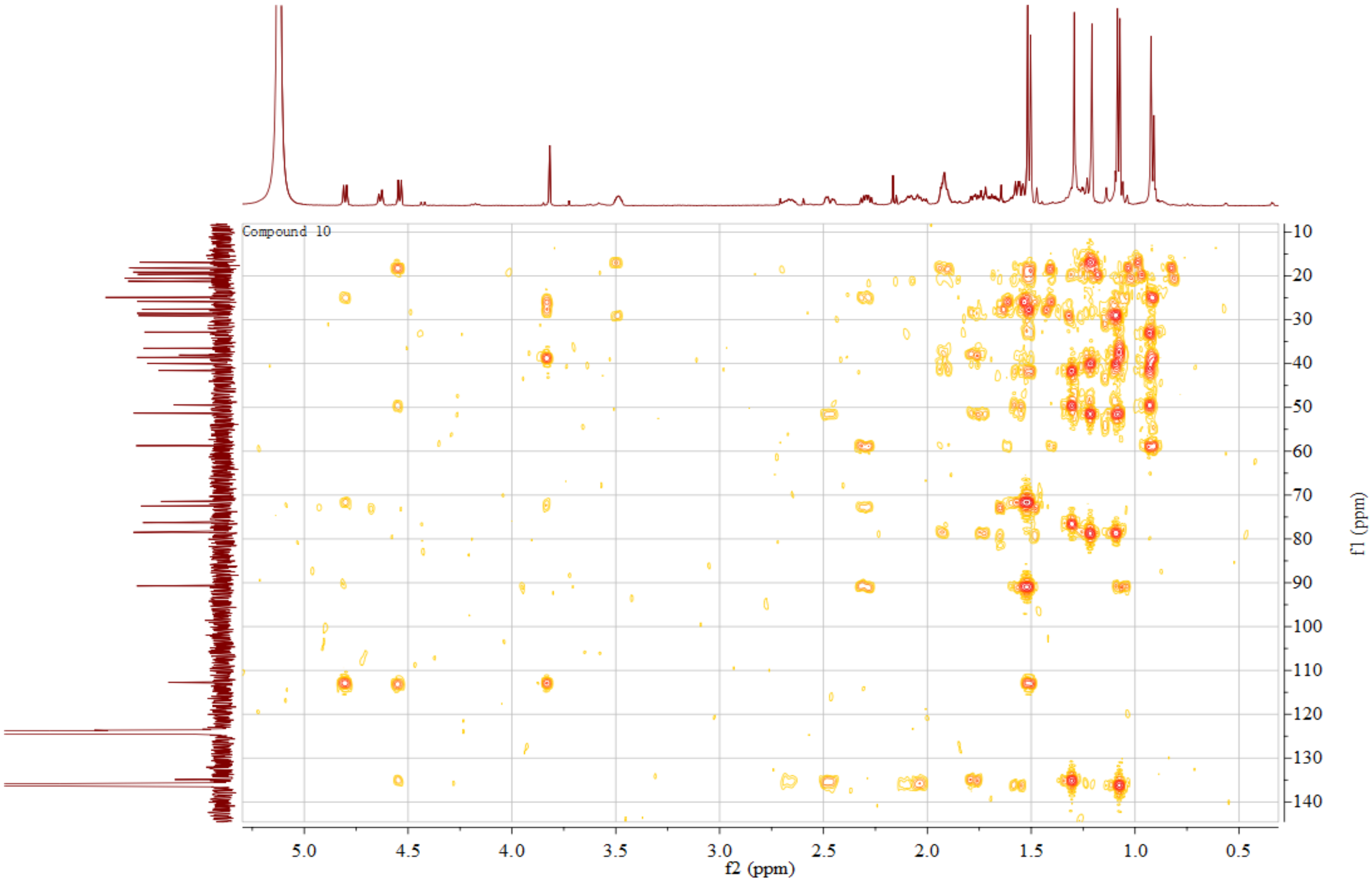

Figure S77. ^{1}H-^{1}H COSY Spectrum of **10** in Pyridine-d_5

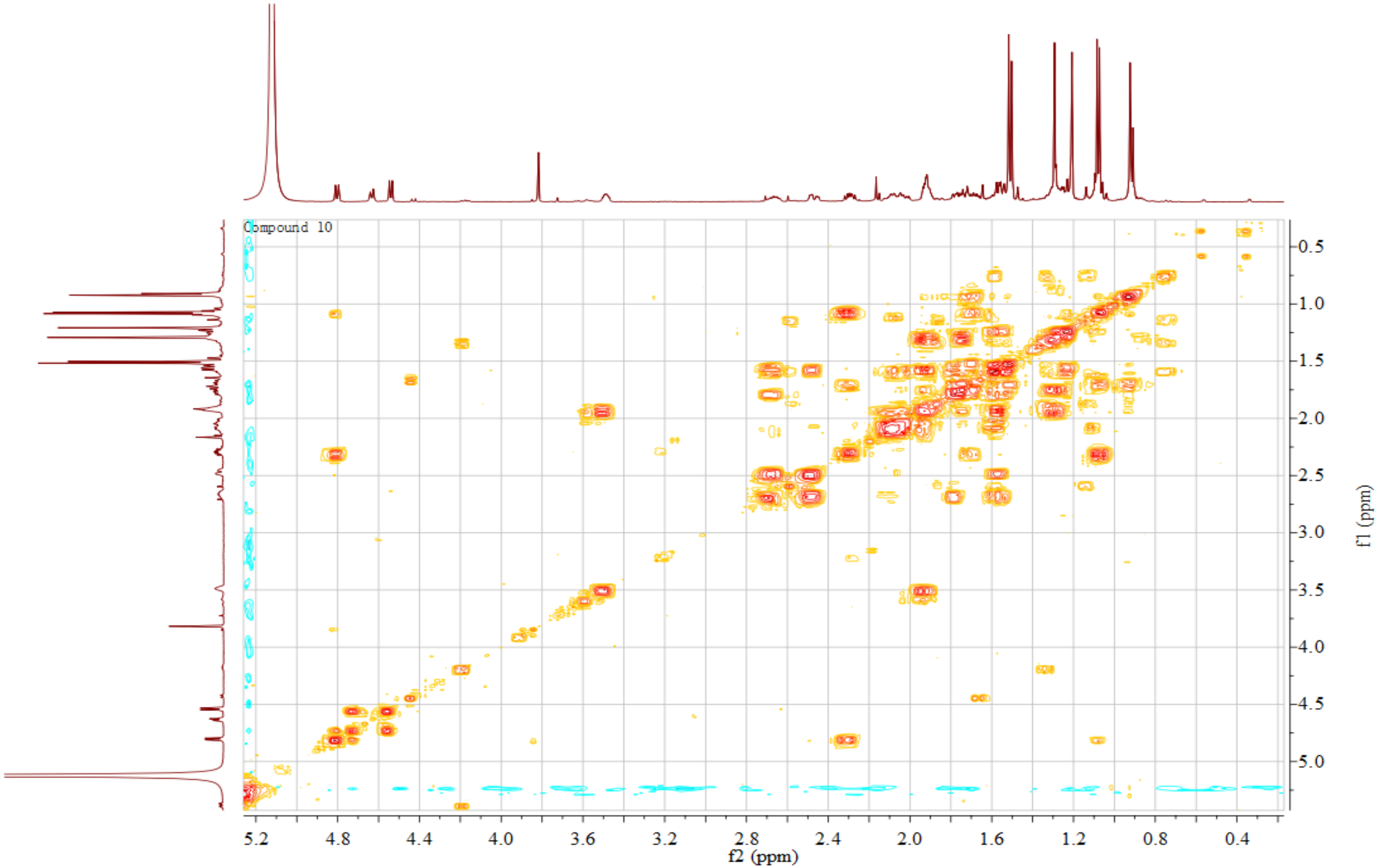

Figure S78. ROESY Spectrum of **10** in Pyridine-d_5

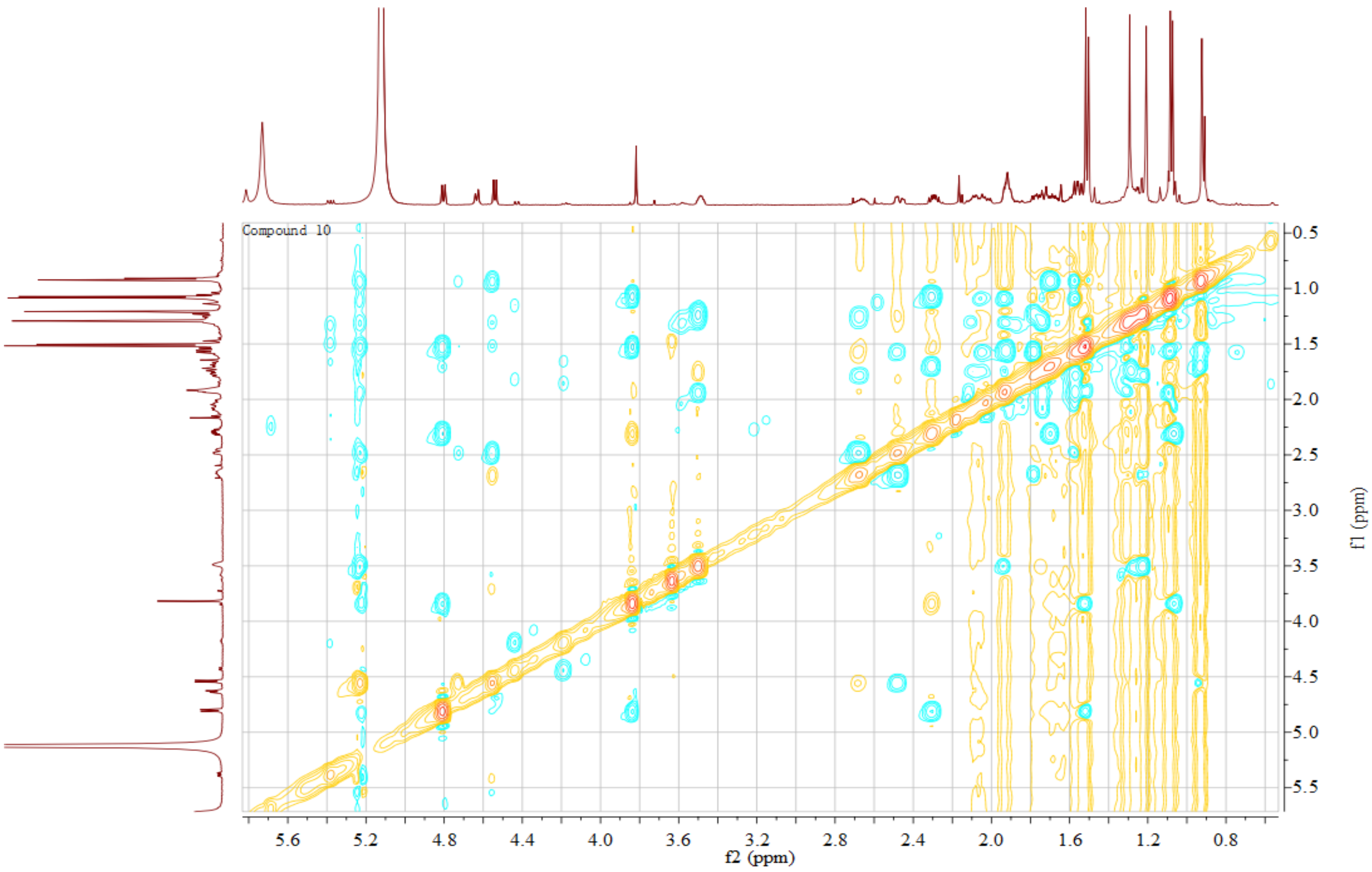

Figure S79. HRESIMS of **10**

Elemental Composition Report

Page 1

Single Mass Analysis

Tolerance = 10.0 PPM / DBE: min = -10.0, max = 120.0
Selected filters: None

Monoisotopic Mass, Odd and Even Electron Ions
21 formula(e) evaluated with 1 results within limits (up to 51 closest results for each mass)
Elements Used:
C: 0-200 H: 0-400 O: 4-6

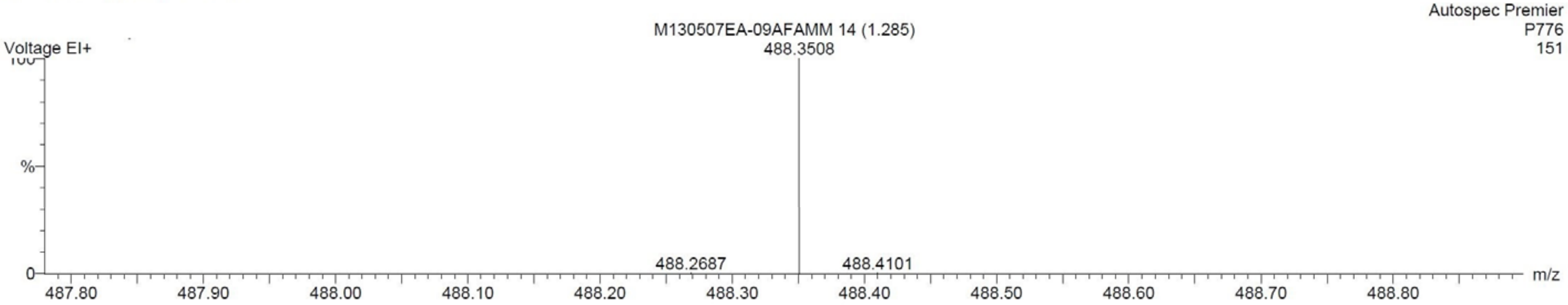

Minimum:				-10.0		
Maximum:		200.0	10.0	120.0		
Mass	Calc. Mass	mDa	PPM	DBE	i-FIT	Formula
488.3508	488.3502	0.6	1.2	7.0	5546094.0	C30 H48 O5

Figure S80. IR Spectrum of **10**

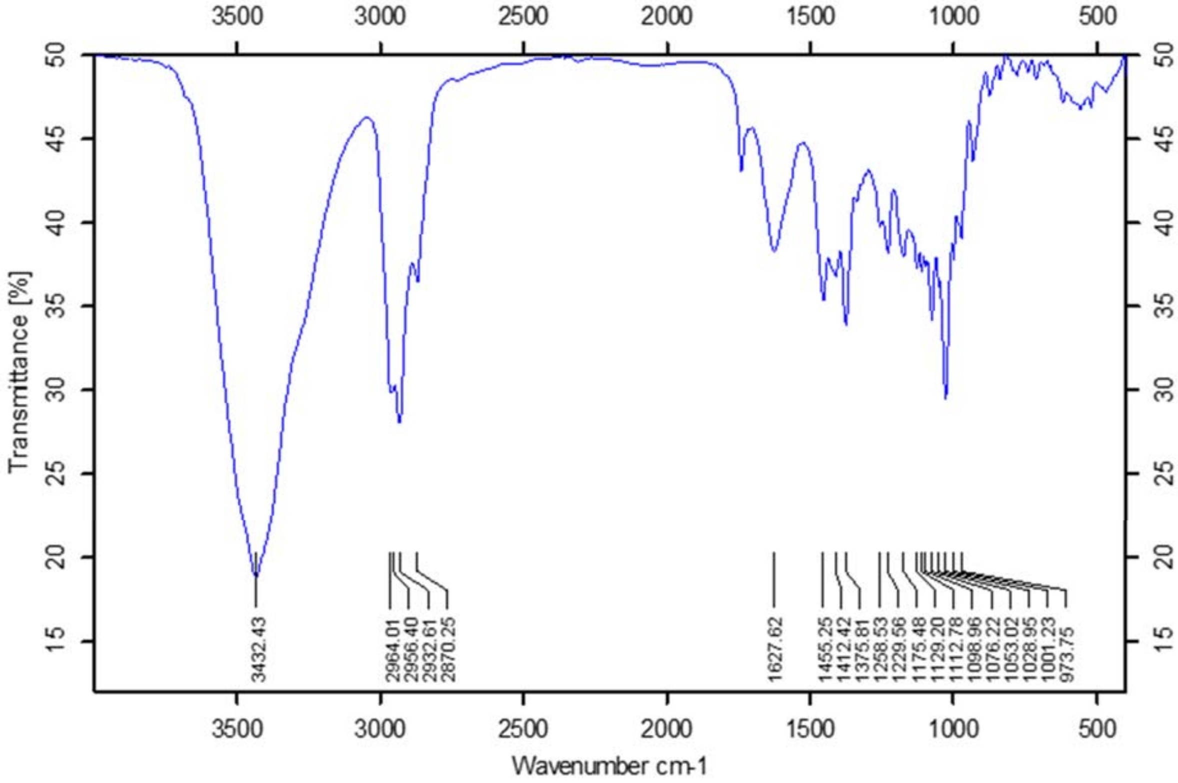

Figure S81. ^{1}H NMR Spectrum of **11** in Pyridine-d_5

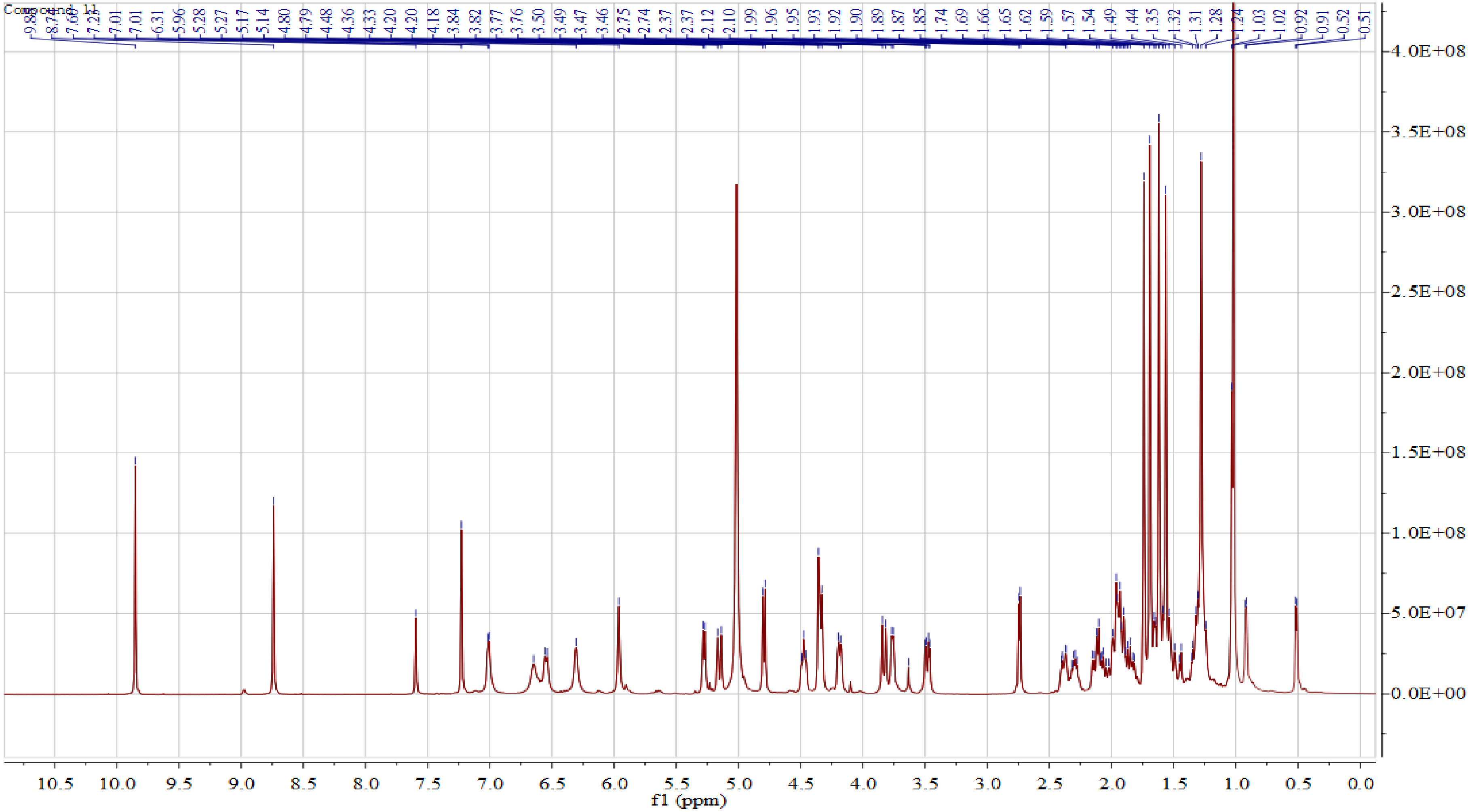

Figure S82 ^{13}C NMR Spectrum of **11** in Pyridine-d_5

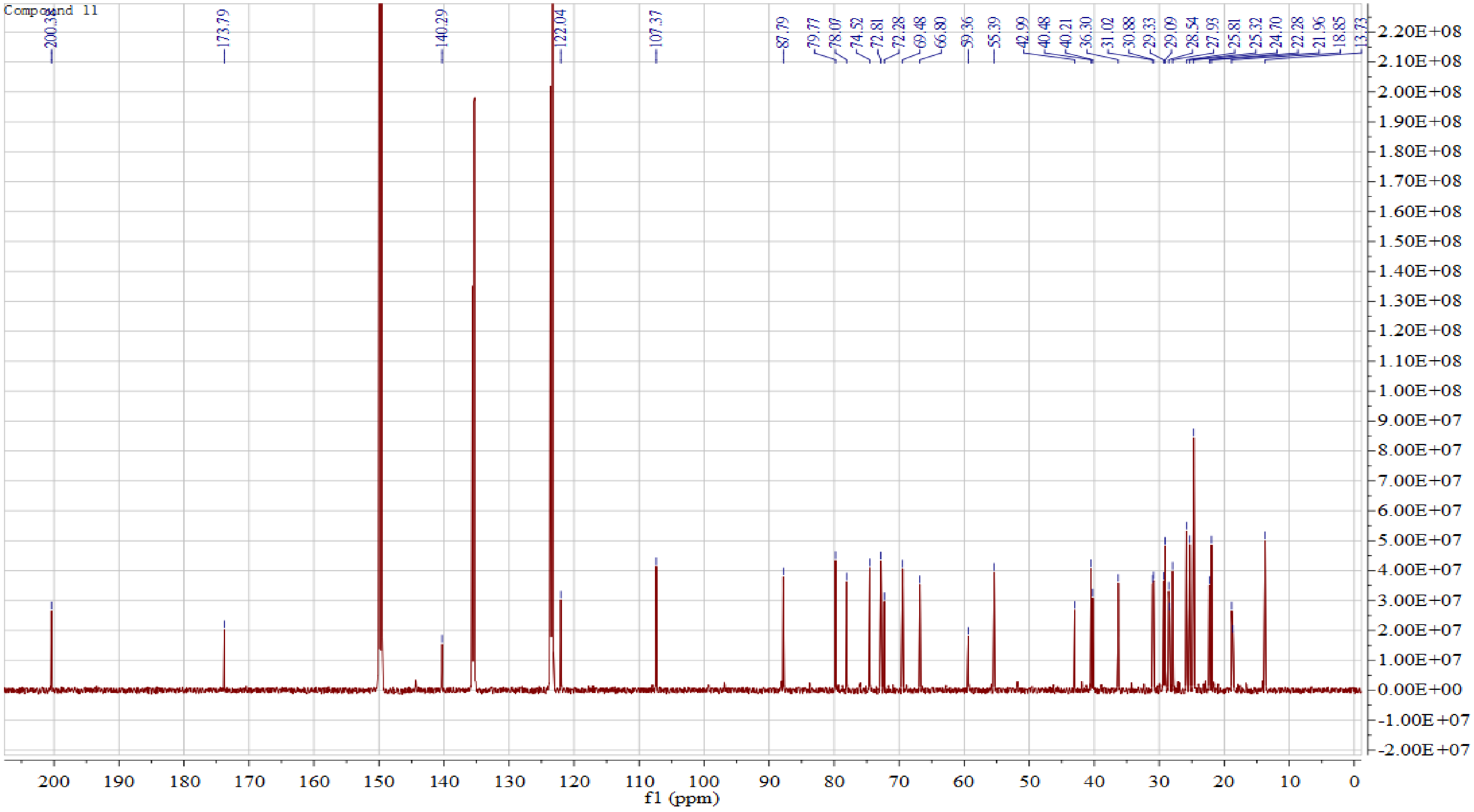

Figure S83. HSQC Spectrum of **11** in Pyridine-d_5

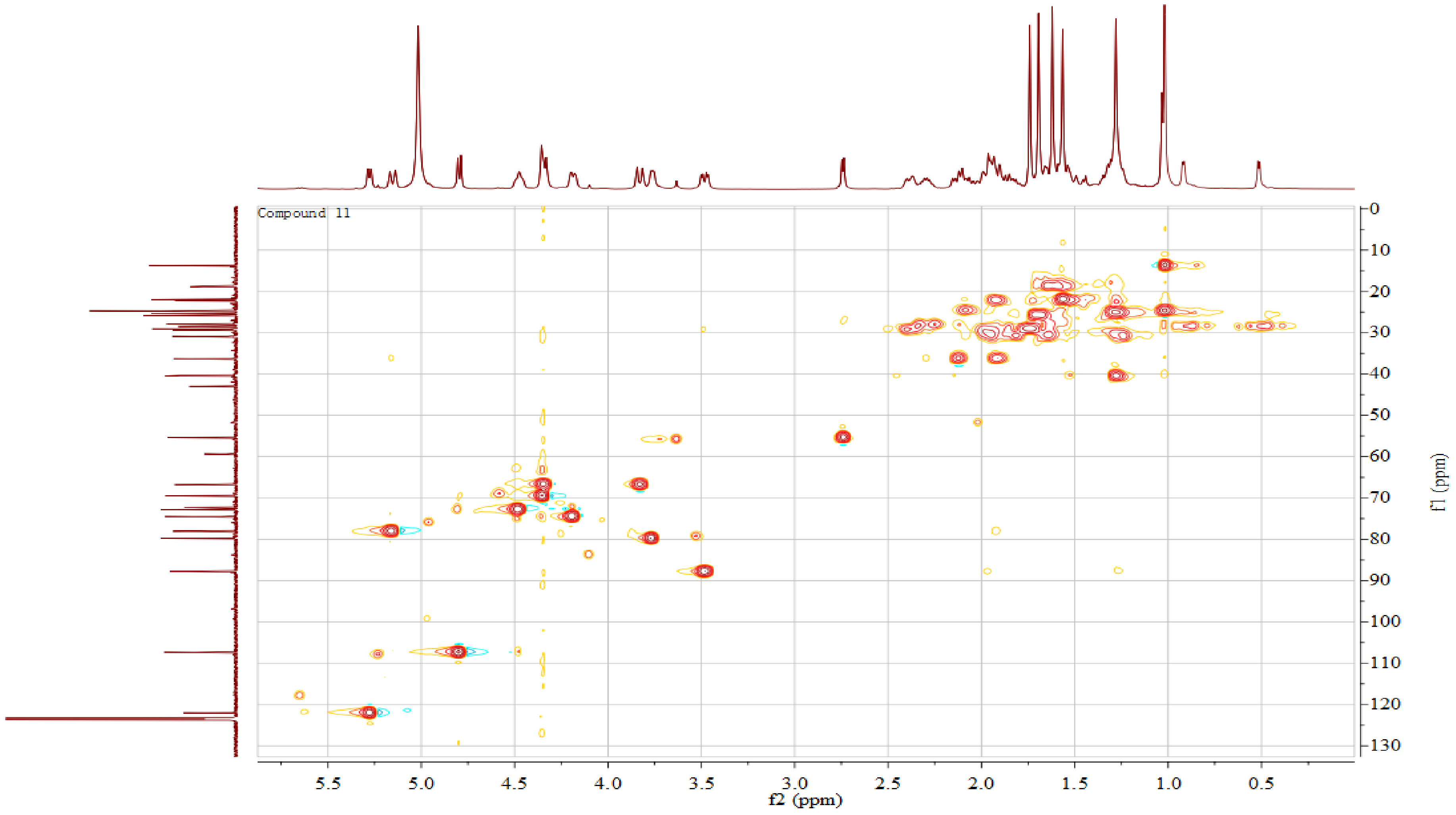

Figure S84. HMBC Spectrum of **11** in Pyridine-d_5

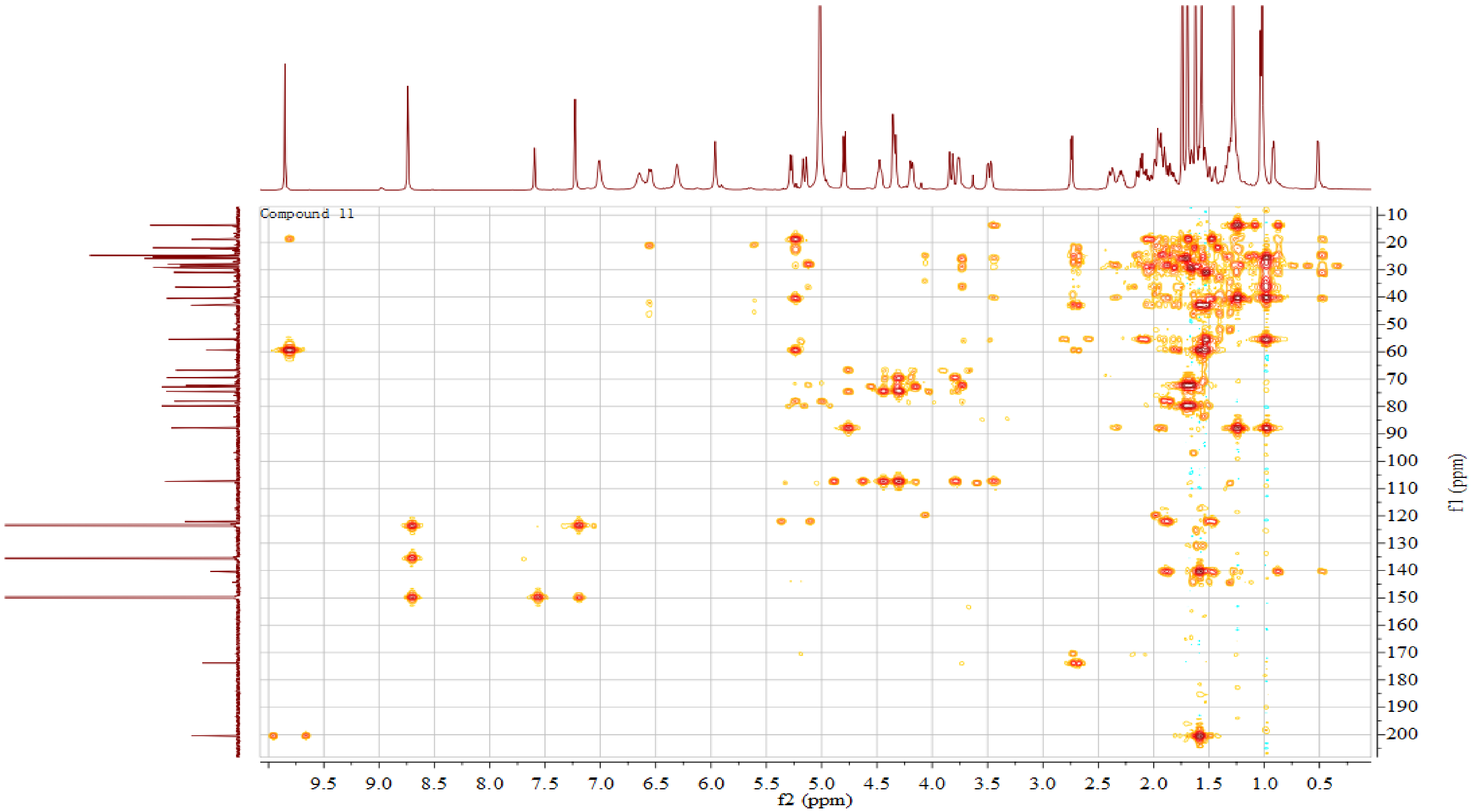

Figure S85. ^{1}H-^{1}H COSY Spectrum of **11** in Pyridine-d_5

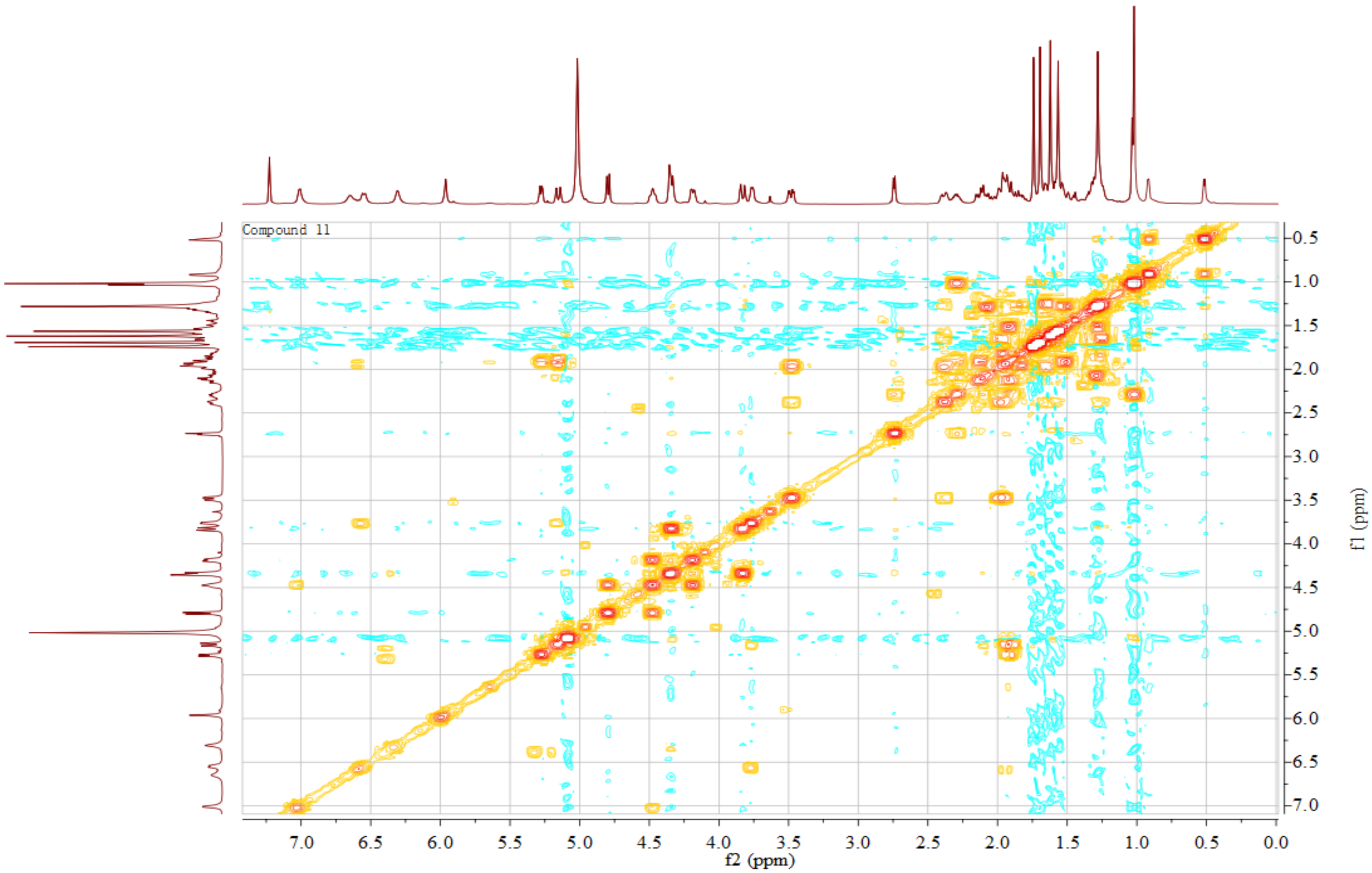

Figure S86. ROESY Spectrum of **11** in Pyridine-d_5

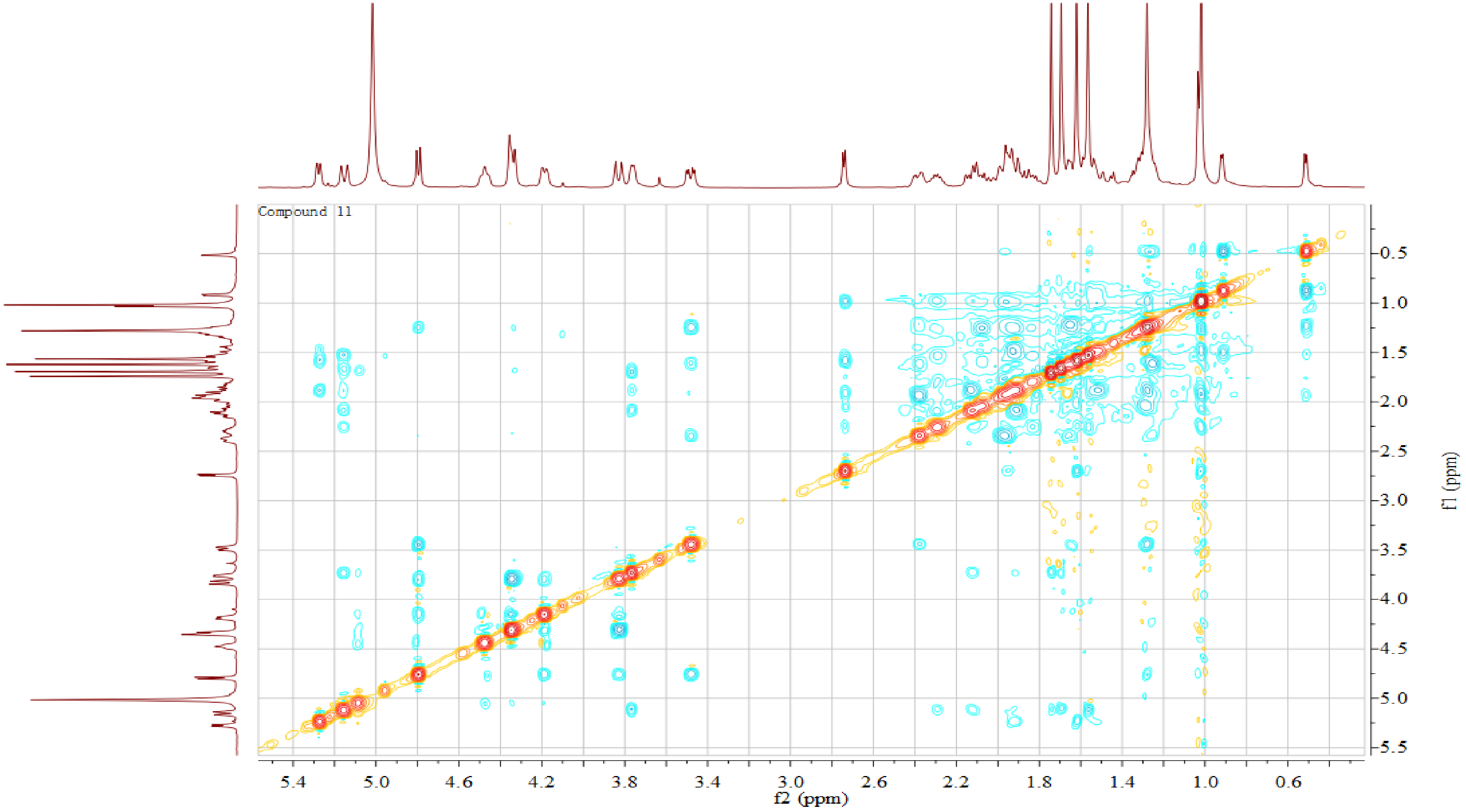

Figure S87. HRESIMS of **11**

Qualitative Analysis Report

**Data Filename**		**Sample Name**	
**Sample Type**	Sample	**Position**	
**Instrument Name**	Agilent G6230 TOF MS	**User Name**	
**Acq Method**	ESI.m	**Acquired Time**	
**IRM Calibration Status**	Success	**DA Method**	ESI.m
**Comment**			

**Sample Group**		**Info.**
**Acquisition SW Version**	6200 series TOF/6500 series Q-TOF B.05.01 (B5125.2)	

User Spectra

Fragmentor Voltage	Collision Energy	Ionization Mode
200	0	ESI

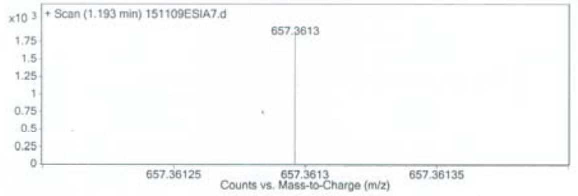

Peak List

m/z	z	Abund
102.1282		9265.63
105.0426		10944.39
121.0509	1	73197.59
153.0544	1	18663.97
232.1118	1	14996.71
241.1696	1	20455.48
253.1405		8963.17
413.2659	1	10650.73
922.0098	1	99200.98
923.0126	1	15305.5

Formula Calculator Element Limits

Element	Min	Max
C	0	200
H	0	400
O	7	12
Na	1	1

Formula Calculator Results

Formula	CalculatedMass	CalculatedMz	Mz	Diff. (mDa)	Diff. (ppm)	DBE
C35 H54 Na O10	657.3615	657.3609	657.3613	-0.4	-0.6	8.5000

--- End Of Report ---

Figure S88. IR Spectrum of **11**

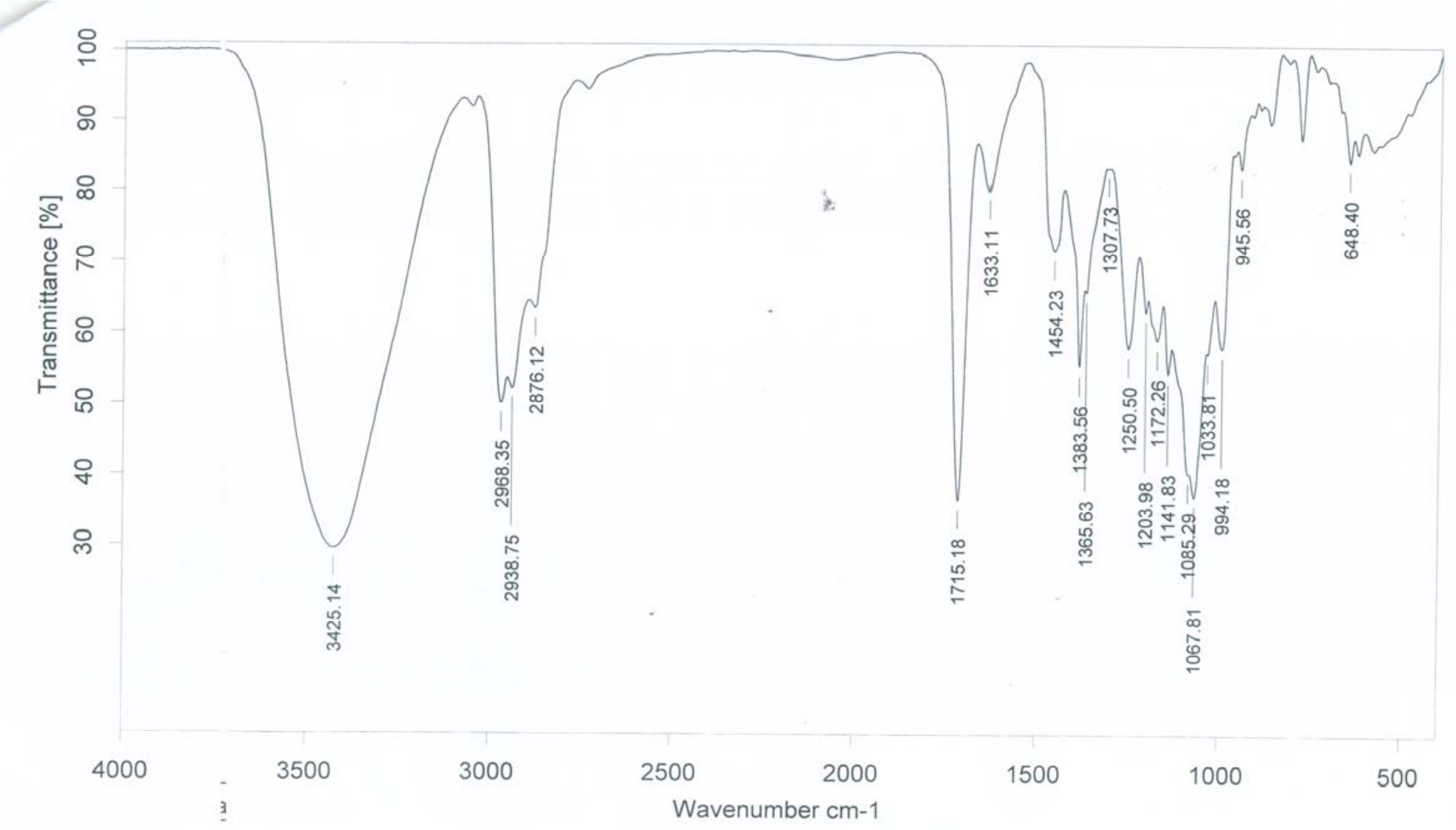

Figure S89. ^{1}H NMR Spectrum of **12** in Pyridine-d_5

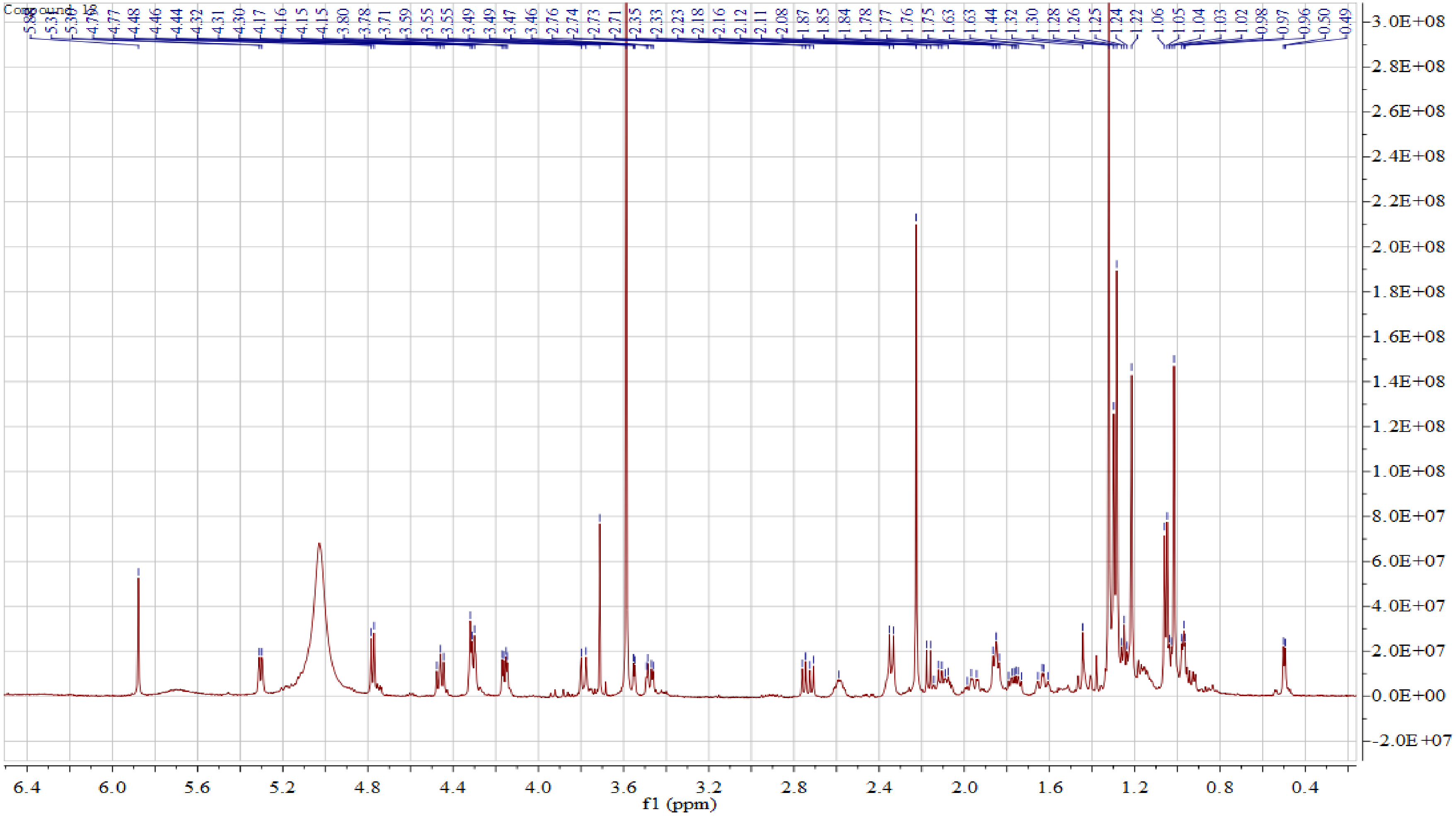

Figure S90 ^{13}C NMR Spectrum of **12** in Pyridine-d_5

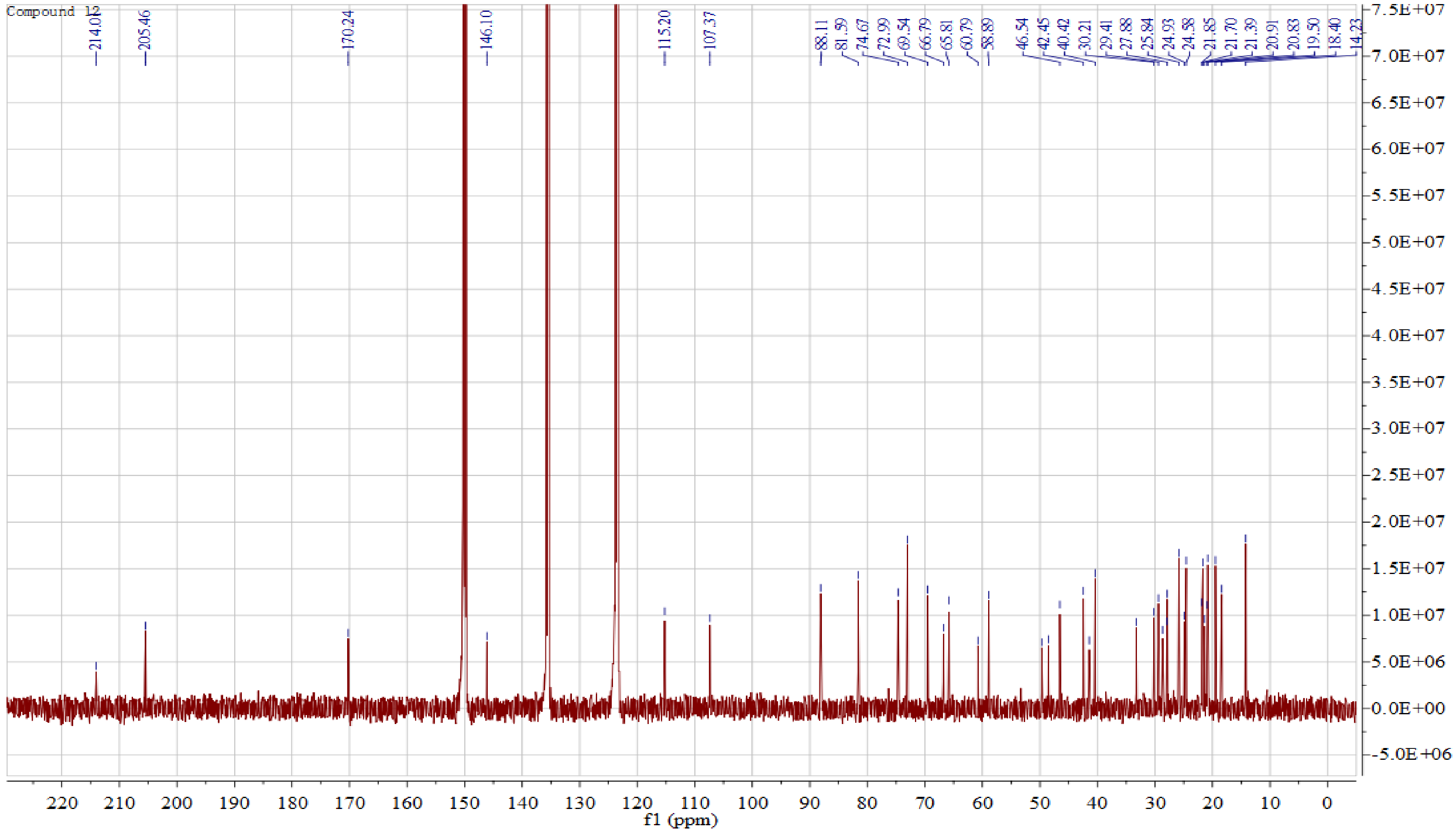

Figure S91. HSQC Spectrum of **12** in Pyridine-*d*$_5$

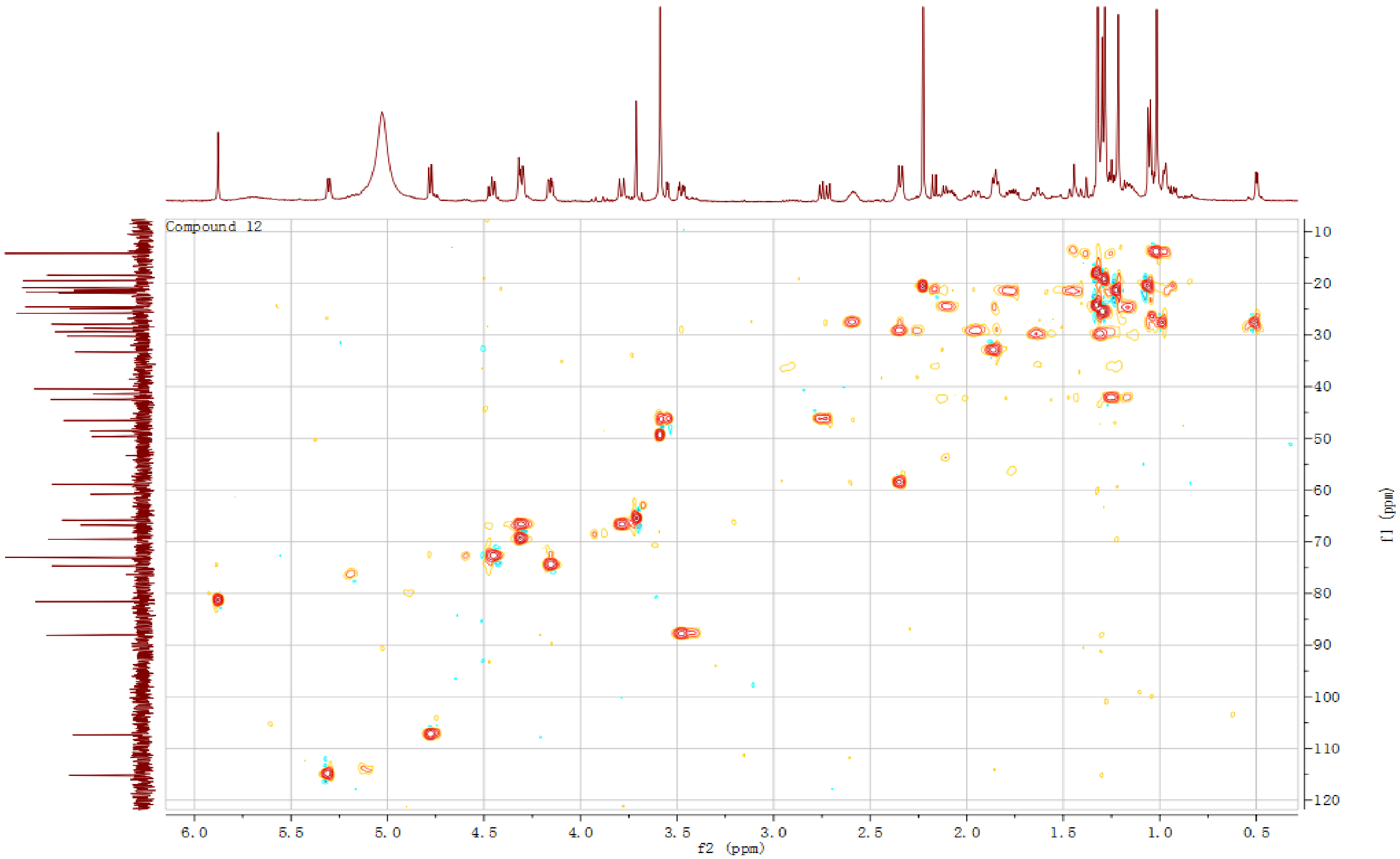

Figure S92. HMBC Spectrum of **12** in Pyridine-*d*5

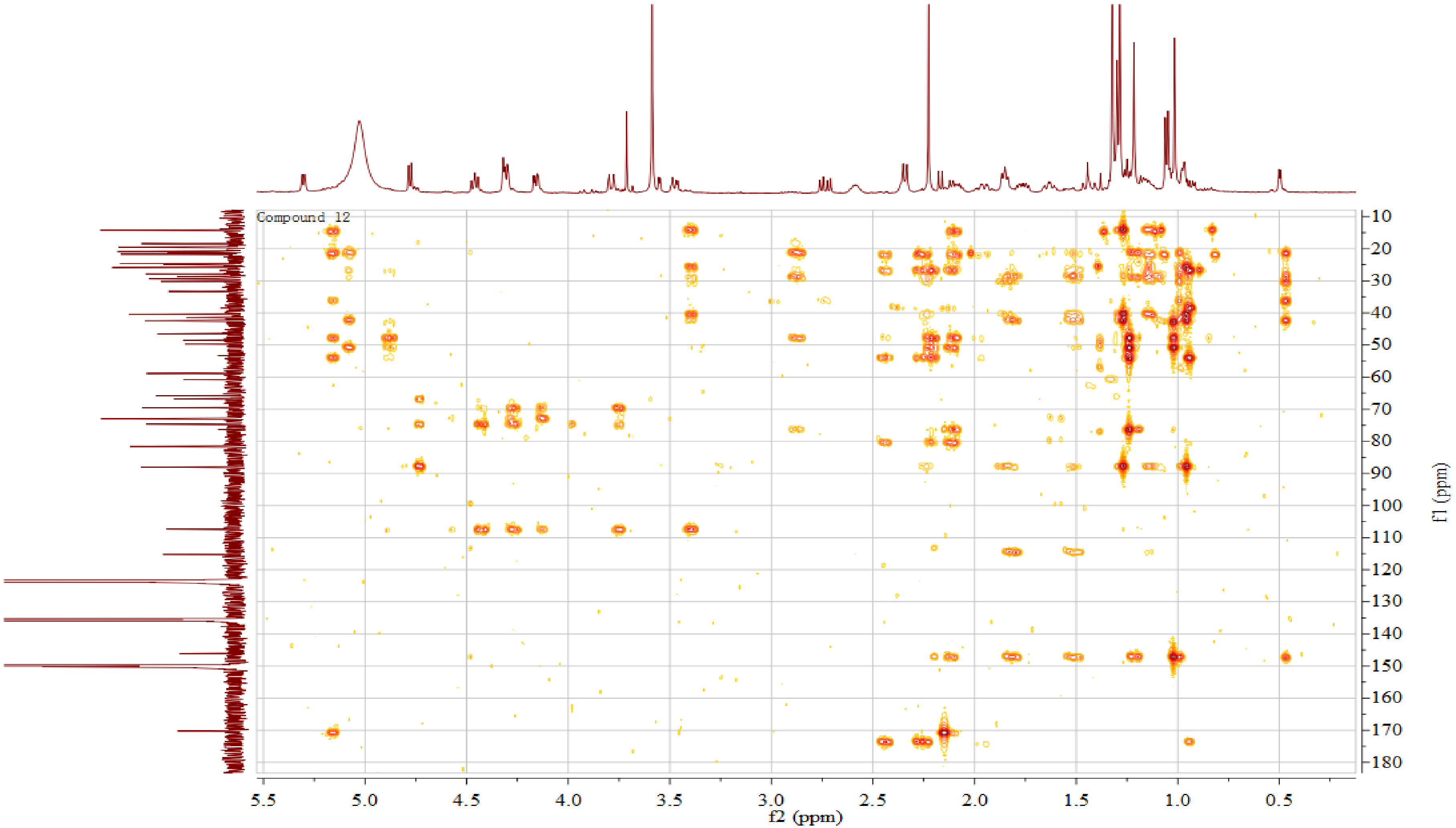

Figure S93. ^{1}H-^{1}H COSY Spectrum of **12** in Pyridine-d_5

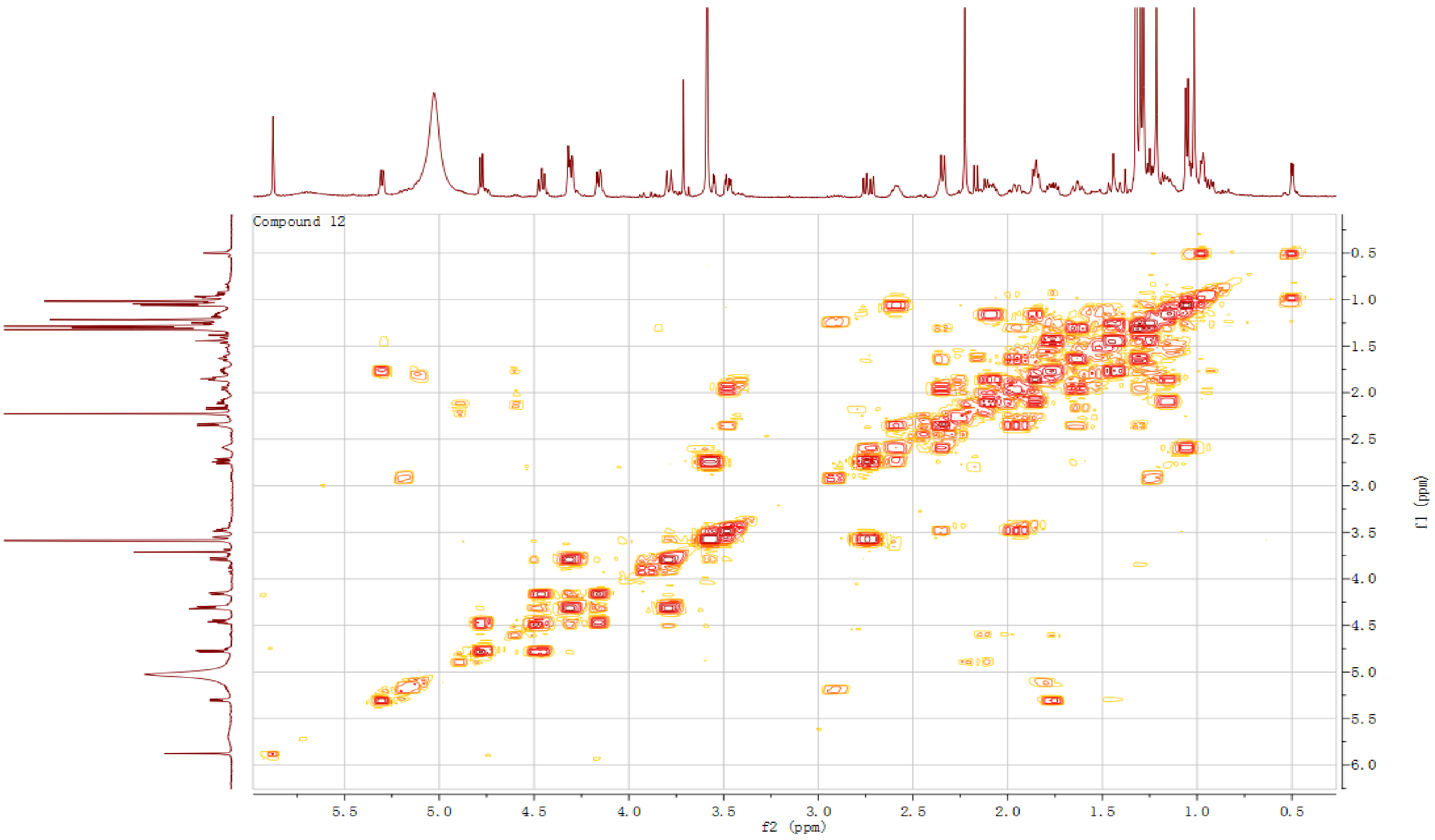

Figure S94. ROESY Spectrum of **12** in Pyridine-d_5

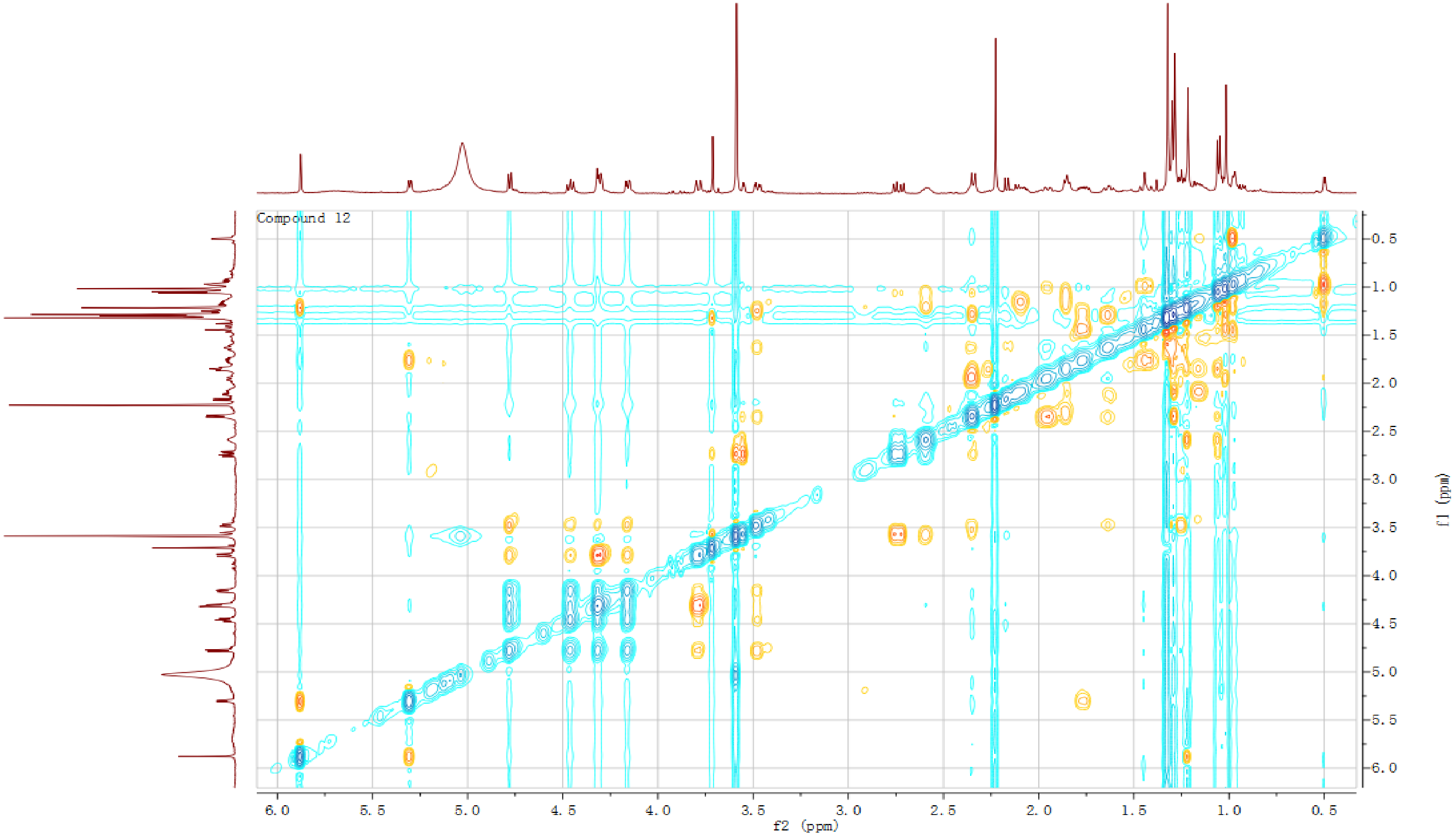

Figure S95. HRESIMS of **12**

Elemental Composition Report

Page 1

Single Mass Analysis

Tolerance = 10.0 PPM / DBE: min = -10.0, max = 120.0
Selected filters: None

Monoisotopic Mass, Odd and Even Electron Ions
24 formula(e) evaluated with 1 results within limits (up to 51 closest results for each mass)
Elements Used:
C: 0-200 H: 0-400 O: 9-11

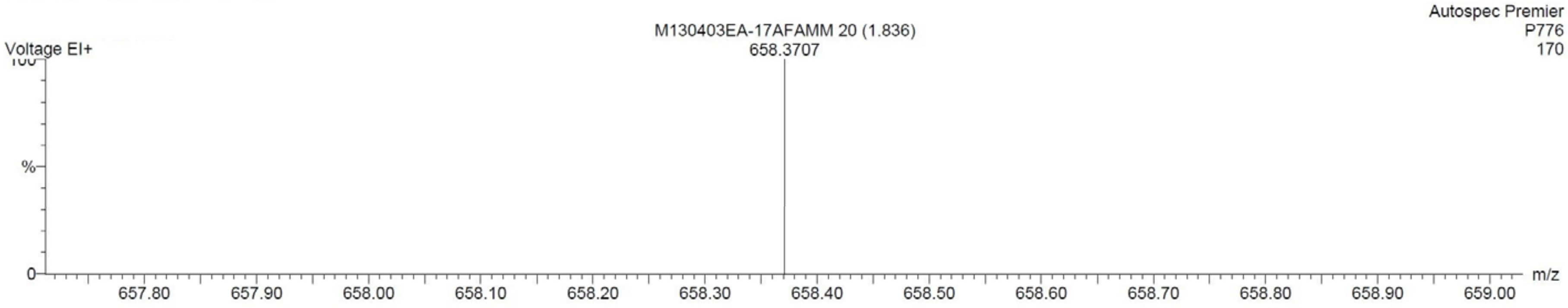

Minimum:				-10.0		
Maximum:		200.0	10.0	120.0		
Mass	Calc. Mass	mDa	PPM	DBE	i-FIT	Formula
658.3707	658.3717	-1.0	-1.5	11.0	5546105.0	C37 H54 O10

Figure S96. IR Spectrum of **12**

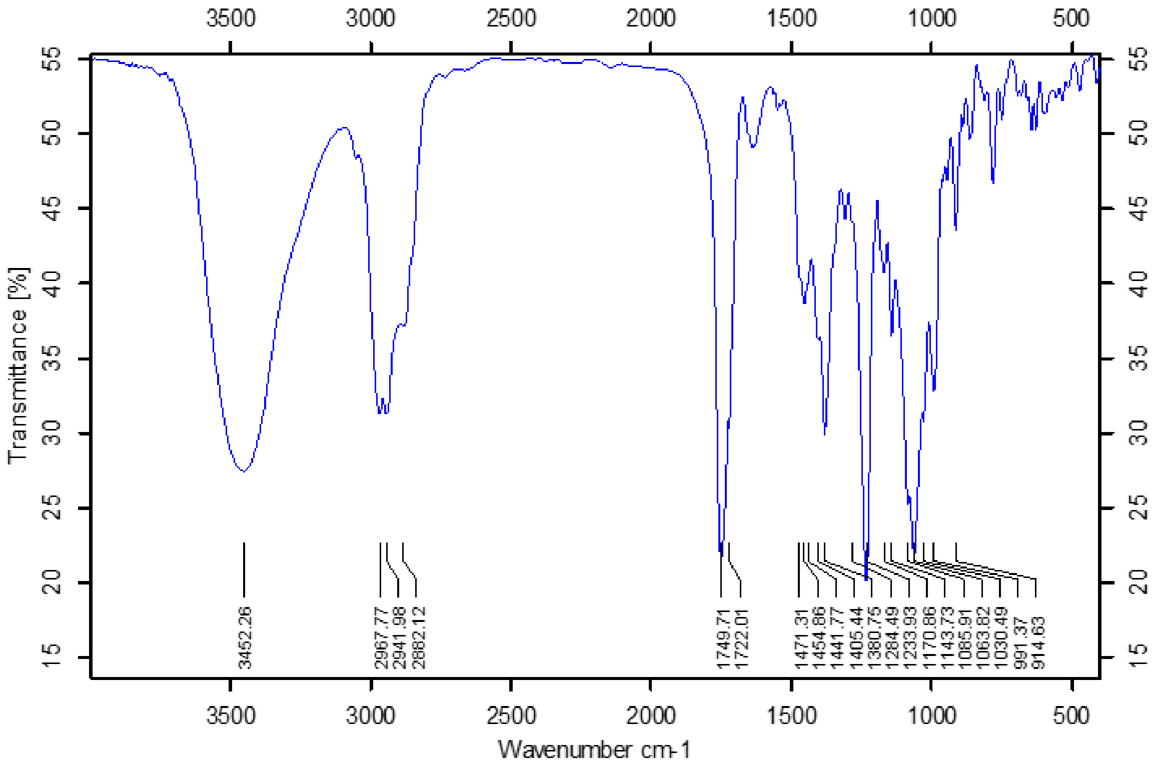

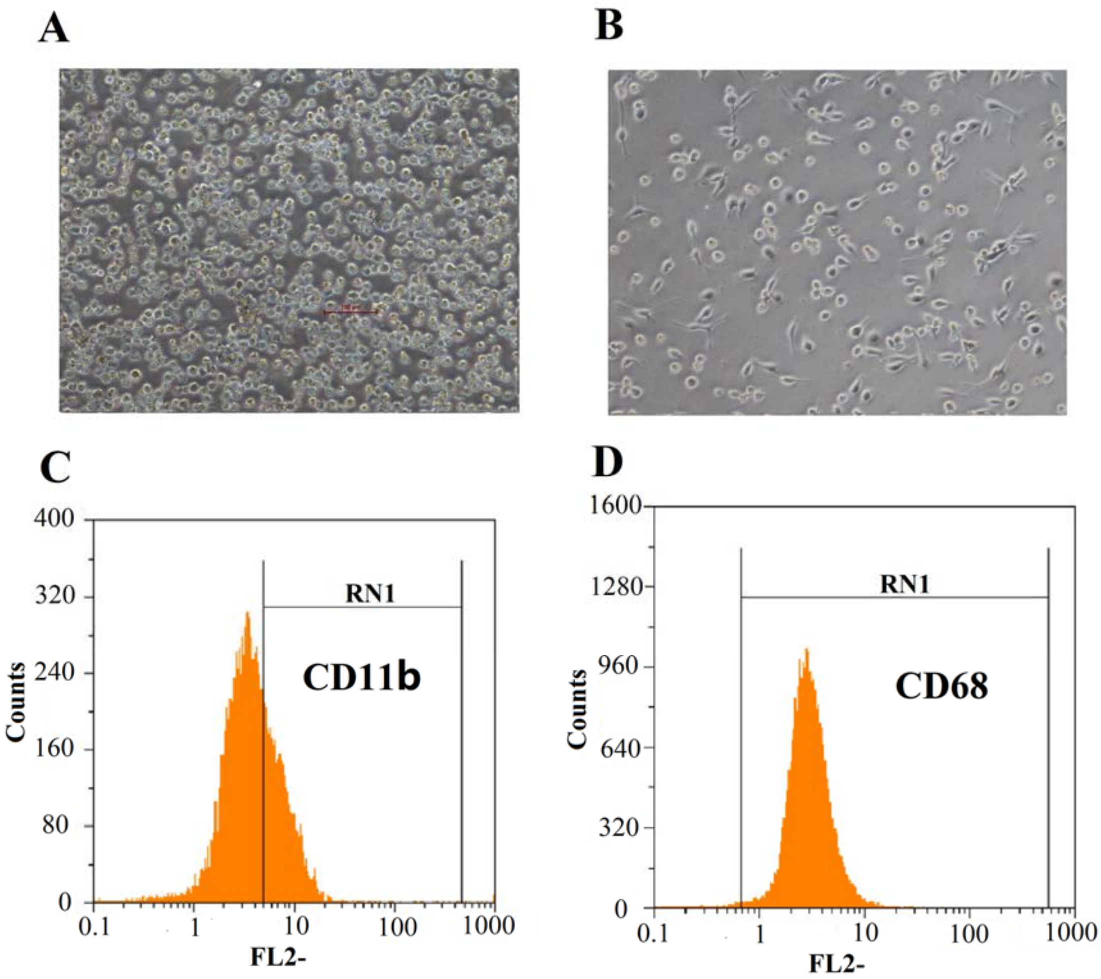

Figure S97. Cell morphology and expression of CD11b and CD68 of differentiated and undifferentiated THP-1 cells. **A.** Undifferentiated THP-1 cells; **B.** Induction of monocyte-macrophage differentiation by 100 nM PMA for 24 h; **C.** CD11b expression of the differentiated cells. **D.** CD68 expression of the differentiated cells; Expressions of CD11b and CD68 were measured by flow cytometry using FITC-labeled anti-CD11b mAb and anti-CD68 mAb. Analyses were conducted on a PARTEC brand flow cytometer.

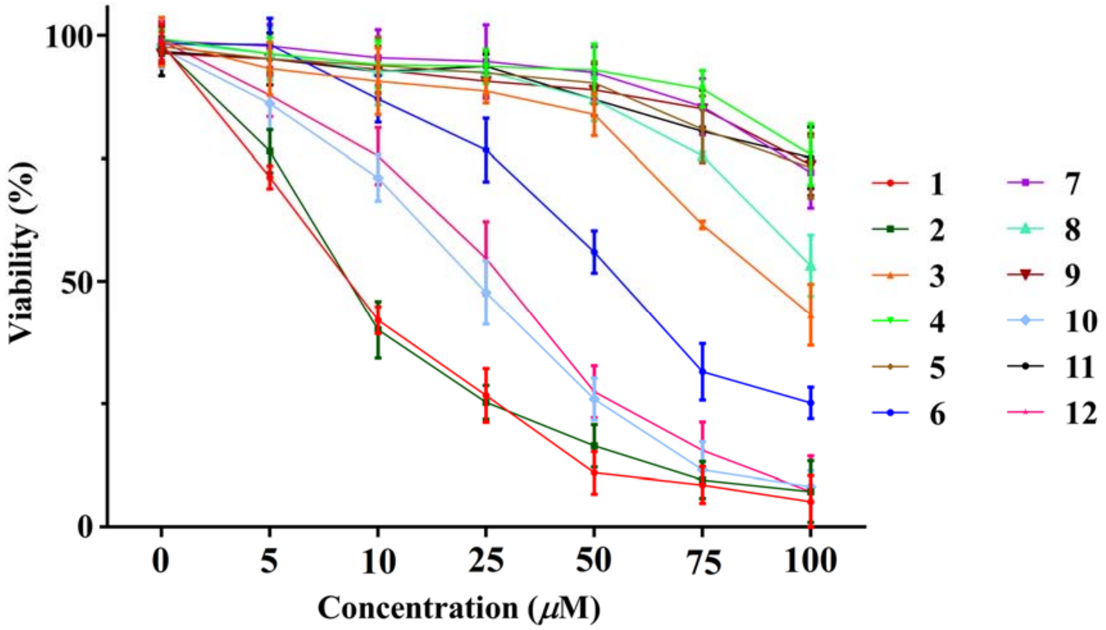

Figure S98. Effects of compounds **1-12** on the viability of PMA-induced THP-1 cells. PMA-induced THP-1 cells were treated with indicated concentrations of compounds **1-12** (0-100 μM) and cell viability was assessed after 48 h using the MTT assay. Data points represent mean ± SD of three measurements.

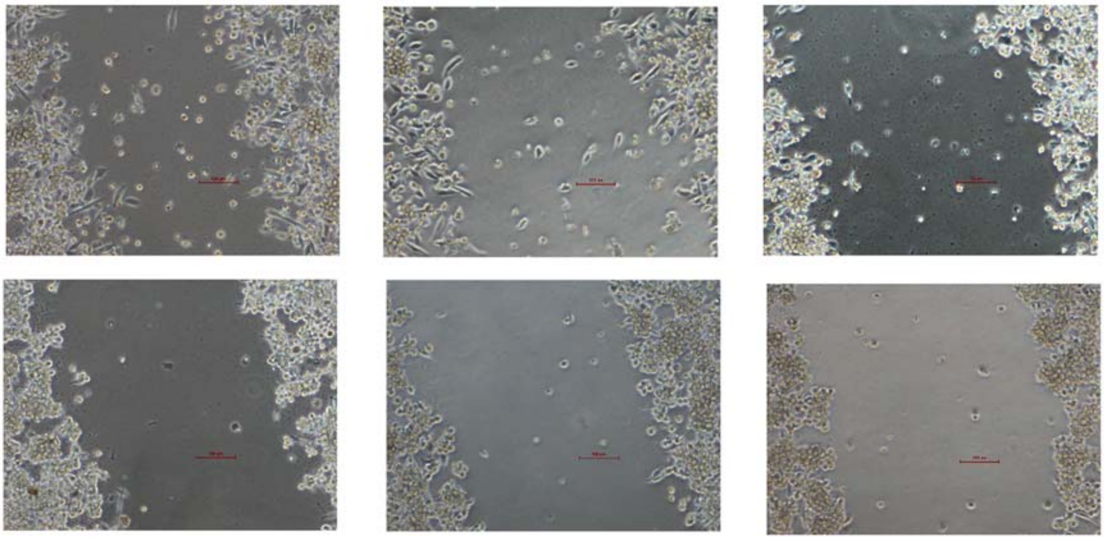

Figure S99. Representative pictures of the migration of PMA-induced THP-1 cells. Up panel: The migration of PMA-induced THP cells without compound **7** (Three parallel experiments); Down panel**:** The migration of PMA-induced THP cells with 50 μM compound **7** (Three parallel experiments).

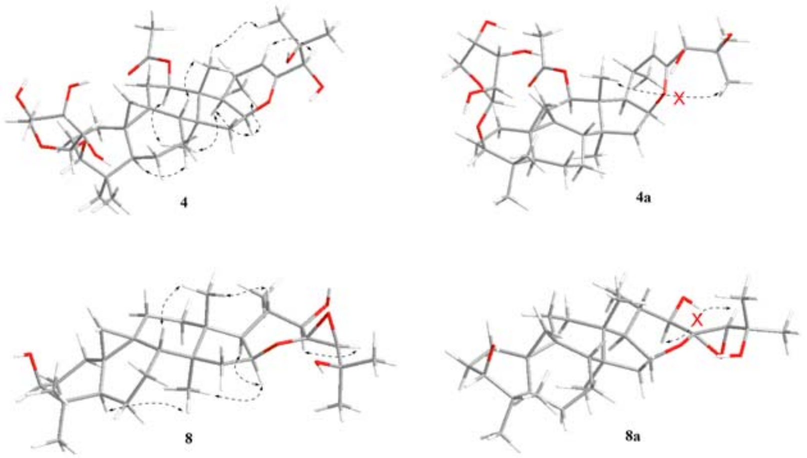

Figure S100. Key ROESY correlations of compounds **4** (**4a**) and **8** (**8a**).

Table S1. Primers' information for CD147 and MMPs in the present study

CTGGTACAAGATCACTGAC	EMMPRIN(H)-F
GAGGAACTCACGAAGAAC	EMMPRIN(H)-R
CTGAAGGACACACTAAAGA	MMP2(H)-F
CGATGGTATTCTGGTCAA	MMP2(H)-R
GGCAGATTCCAAACCTTT	MMP9(H)-F
GCAAGTCTTCCGAGTAGT	MMP9(H)-R
AAAGGGTCATCATCTCTG	GAPDH-F
GCTGTTGTCATACTTCTC	GAPDH-R
